# Gossypetin ameliorates 5xFAD spatial learning and memory through enhanced phagocytosis against Aβ

**DOI:** 10.1186/s13195-022-01096-3

**Published:** 2022-10-21

**Authors:** Kyung Won Jo, Dohyun Lee, Dong Gon Cha, Eunji Oh, Yoon Ha Choi, Somi Kim, Eun Seo Park, Jong Kyoung Kim, Kyong-Tai Kim

**Affiliations:** 1 Department of Life Sciences, Pohang University of Science and Technology (POSTECH), Pohang, Gyeongbuk 37673 Republic of Korea; 2 R&D Center, NovMetaPharma Co., Ltd, Pohang, Gyeongbuk 37668 Republic of Korea; 3 Department of New Biology, DGIST, Daegu, 42988 Republic of Korea

**Keywords:** Gossypetin, Alzheimer’s disease, Beta-amyloid (Aβ), Phagocytosis, Disease-associated microglia, Single-cell RNA sequencing

## Abstract

**Background:**

Microglia are the resident immune cells found in our brain. They have a critical role in brain maintenance. Microglia constantly scavenge various waste materials in the brain including damaged or apoptotic neurons and Aβ. Through phagocytosis of Aβ, microglia prevent the accumulation of Aβ plaque in the brain. However, in Alzheimer’s disease (AD) patients, chronic exposure to Aβ makes microglia to become exhausted, which reduces their phagocytic activity against Aβ. Since microglia play an important role in Aβ clearance, enhancing microglial phagocytic activity against Aβ is a promising target for AD treatment. Therefore, there is a great need for therapeutic candidate that enhances microglial Aβ clearance while inhibiting microglia’s pathogenic properties.

**Methods:**

In vivo studies were conducted with 5xFAD AD model mice by treating gossypetin for 13 weeks through intragastric administration. Their spatial learning and memory were evaluated through behavior tests such as Y-maze and Morris Water Maze test. Hippocampus and cortex were acquired from the sacrificed mice, and they were used for histological and biochemical analysis. Also, mouse tissues were dissociated into single cells for single-cell RNA sequencing (scRNA-seq) analysis. Transcriptome of microglial population was analyzed. Mouse primary microglia and BV2 mouse microglial cell line were cultured and treated with fluorescent recombinant Aβ to evaluate whether their phagocytic activity is affected by gossypetin.

**Results:**

Gossypetin treatment improved the spatial learning and memory of 5xFAD by decreasing Aβ deposition in the hippocampus and cortex of 5xFAD. Gossypetin induced transcriptomic modulations in various microglial subpopulations, including disease-associated microglia. Gossypetin enhanced phagocytic activity of microglia while decreasing their gliosis. Gossypetin also increased MHC II^+^ microglial population.

**Conclusions:**

Gossypetin showed protective effects against AD by enhancing microglial Aβ phagocytosis. Gossypetin appears to be a novel promising therapeutic candidate against AD.

**Supplementary Information:**

The online version contains supplementary material available at 10.1186/s13195-022-01096-3.

## Background

Alzheimer’s disease (AD) is the most prevalent neurodegenerative disease which its cure has not been developed for decades. Numerous drugs have been developed to decrease the production of Aβ, which is the most well-known hallmark of AD. However, previous drugs have all failed in clinical trials due to their adverse side effects or lack of efficacy [1]. Therefore, the strategy for AD drug developments have started to shift from preventing Aβ to decreasing the aggregation and increasing the clearance of Aβ [2, 3]. Microglia are the resident immune cells found in the CNS [4]. Microglial cells play central role in decreasing Aβ level in our brain through various ways. Microglia directly recognize, uptake and degrade Aβ through phagocytosis. They also release various Aβ degrading enzymes such as neprilysin or insulin degrading enzyme (IDE) [5]. Therefore, enhancing the efficiency of Aβ clearance by microglia is a promising target to treat AD.

Flavonoids are natural phenolic compounds derived from plants, especially in fruits and vegetables, as secondary metabolites. Numerous flavonoids are already known to have various beneficial effects to our health. Therefore, flavonoid compounds are widely used in pharmaceutical, nutraceutical, cosmeceutical, and medical fields [6]. Reports suggest that various flavonoids may also have therapeutic potential against AD [6–9]. However, there are still numerous flavonoid compounds which its efficacy against AD has not been evaluated thoroughly.

Gossypetin is a flavonoid that is found in the calyx of *Hibiscus sabdariffa*, which has been reported to have antioxidant, anti-atherosclerotic, and anticancer activity through inhibition of LDL oxidation, MKK3, and MKK6 activity [10, 11]. Gossypetin has a very similar chemical structure to quercetin and morin which were previously reported to be effective against AD [6, 8, 9]. There was also a report that showed inhibitory effect of gossypetin against Aβ and tau aggregation in vitro, which suggested the therapeutic potential of gossypetin against AD [12]*.* However, no research has validated the therapeutic potential of gossypetin against AD through in vivo models yet. Therefore, in this study we evaluated the effect and mechanism of gossypetin on spatial learning and memory of 5xFAD mouse, a mouse model of AD.

## Results

### Gossypetin improves spatial learning and memory of 5xFAD mice

**Fig. 1:**
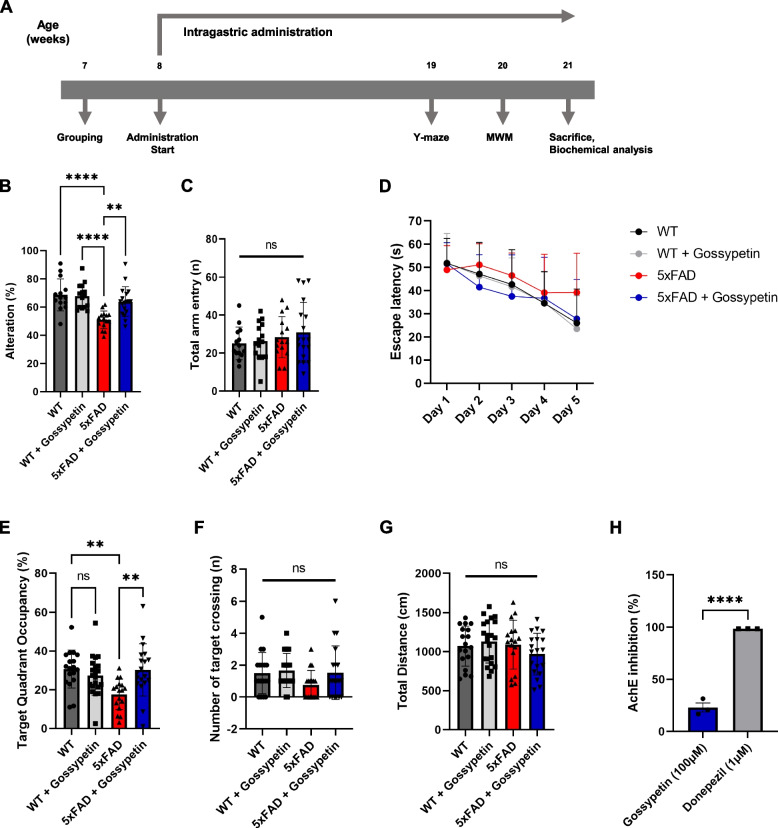
Gossypetin ameliorates spatial learning and memory of 5xFAD mice. **A** Timeline of gossypetin administration for 5xFAD mice (*n* = 17 ~ 21 per group). **B**, **C** 5xFAD mice were tested with Y-maze alteration test measuring percentage of alteration (**B**) and number of total arm entry (**C**). **D**–**G** 5xFAD mice were trained for 5 days for Morris Water Maze test, and escape latency was recorded each day of training. Probe test was conducted on the 6th day (**D**). In probe test, percentage of target (platform) quadrant occupancy (**E**), number of target crossing (**F**), and total traveled distance were recorded (**G**). **H** Bar graph represents AChE activity inhibition percentage of gossypetin (100μM) with donepezil (1μM) as positive control. The error bars represent the mean ± SD (B-G) or mean ± SEM (H), **** *p* < 0.0001, **p < 0.01, *p < 0.05, ns = not significant, two-way ANOVA followed by Tukey’s multiple comparisons test (**B**, **C**, **E**, **F**, **G**), and Student’s *t*-test (**H**)

To evaluate the therapeutic potential of gossypetin in AD, 5xFAD mice were treated with gossypetin through intragastric administration (Fig. 1A). 5xFAD is an AD model mouse that expresses human APP and PSEN1 transgenes with familial AD-linked mutations. These mutant APP and PSEN1 drive rigorous accumulation of Aβ in the brain and lead to progression of AD pathology [13]. We used only female mice for our experiments because they were reported to show more profound AD pathology [14]. After the drug treatment, spatial learning and memory were evaluated through a Y-maze alteration test and a Morris Water Maze (MWM) test. Y-maze measures short term, spatial working memory [15]. In the Y-maze test, 5xFAD mice treated with gossypetin showed a remarkable improvement in alteration percentage compared to 5xFAD mice treated with vehicle (Fig. 1B). There was no difference in total arm entry between each group (Fig. 1C). We also conducted MWM test to evaluate hippocampus dependent spatial learning and memory. The mice were trained for 5 days prior to a probe test for MWM (Fig. 1D). In the probe test, we observed a significant increase in target quadrant occupancy in the 5xFAD mice treated with gossypetin (Fig. 1E). Although there was no statistical significance, gossypetin-treated 5xFAD mice showed an increased trend in the number of target crossings compared to vehicle treated 5xFAD mice (Fig. 1F). There was no difference in traveled distance during the probe test (Fig. 1G). Various flavonoid compounds show an inhibitory effect against acetylcholinesterase (AChE) [16]. To confirm whether gossypetin had induced the improvement in behavior through AChE inhibition, we performed in vitro AChE inhibition assay. Gossypetin showed a very weak AChE inhibitory effect even at a very high concentration (100 μM) when compared to donepezil (1 μM), which was used as a positive control (Fig. 1H). Taken together, these results suggest that gossypetin ameliorates the impaired spatial learning and memory found in 5xFAD mice.

### Gossypetin decreases Aβ level in the hippocampus and cortex of 5xFAD mice

**Fig. 2:**
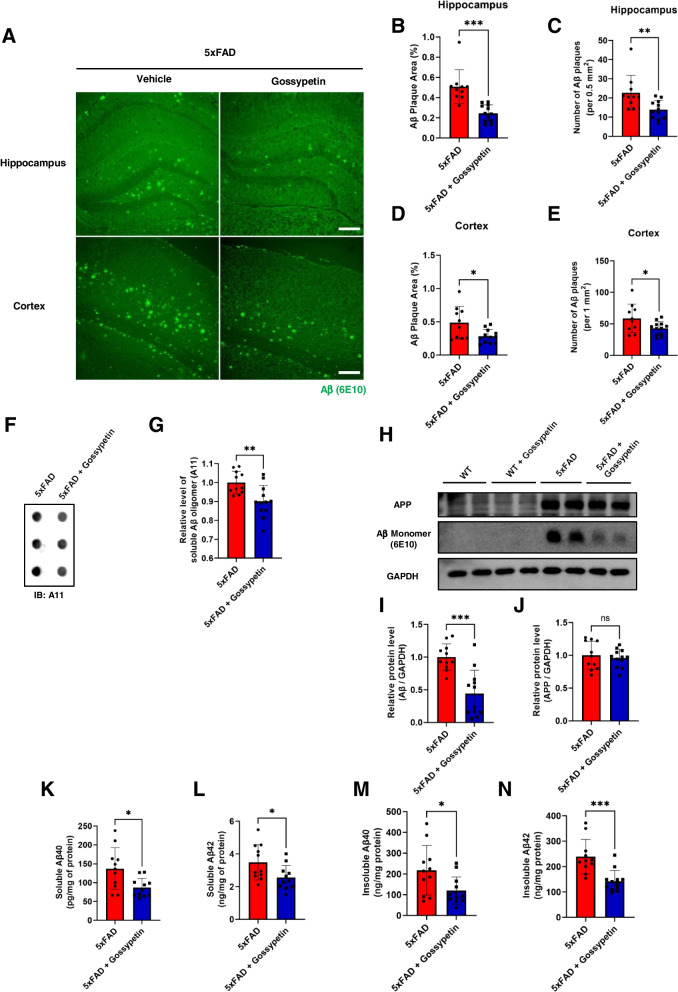
Gossypetin decreases Aβ level in 5xFAD mice. **A**–**E** Representative image of Aβ plaques (6E10) in hippocampus and cortex of 5xFAD and gossypetin treated 5xFAD mice. Scale bar corresponds to 200μm (**A**). Bar graph represents quantification of Aβ plaque area (**B**) and number of Aβ plaque (**C**) in dentate gyrus of hippocampus (*n* = 10~12 mice per group, 3~11 slices per brain). Percentage of Aβ plaque area (**D**) and number of Aβ plaque (**E**) were measured in cortex as well (*n* = 10~12 mice per group, 3~10 slices per brain). **F**, **G** Level of Aβ oligomers (A11) were compared through dot blot assay (**F**). Bar graph represents quantification of relative level of Aβ oligomers (**G**). **H**–**J** Expression level of APP, Aβ and GAPDH in hippocampus were examined through Western blot analysis (**H**). Bar graph represents quantification of protein level of Aβ monomer (**I**) and APP (**J**) normalized by GAPDH. **K**–**N** enzyme-linked immunosorbent assay (ELISA) was conducted to quantify the level of soluble Aβ40 (**K**), Aβ42 (**L**), insoluble Aβ40 (**M**), and Aβ42 (**N**) in the hippocampus (*n* = 11~12 mice per group). The error bars represent the mean ± SD (G, I-N) or mean ± SEM (B-E). ***p < 0.001, **p < 0.01, *p < 0.05, ns = not significant, Student’s *t* test

Gossypetin was reported to have an inhibitory effect against Aβ aggregation in vitro [12]. Therefore, we conducted immunohistochemistry to determine whether the gossypetin improves the behaviors of 5xFAD mice by decreasing Aβ plaques in the brains of 5xFAD mice. We found that gossypetin significantly decreased the number and size of Aβ plaques in the dentate gyrus of the hippocampus and in the cortex of 5xFAD mice (Fig. 2A–E). To validate whether gossypetin was able to decrease other forms of Aβ, we measured the level of Aβ oligomer through dot blot assay. We observed slight decrease of Aβ oligomer in hippocampal lysate of 5xFAD treated with gossypetin (Fig. 2F, G). Then, we conducted Western blot to see whether the level of Aβ monomer was affected (Fig. 2H). Surprisingly, gossypetin significantly decreased Aβ monomer level in the hippocampus as well (Fig. 2I), while the level of amyloid precursor protein (APP) was unaffected (Fig. 2J). To further confirm whether gossypetin decreased the overall level of Aβ, we quantified the level of Aβ in the hippocampus through enzyme-linked immunosorbent assay. We were able to observe that the soluble and insoluble Aβ40 and Aβ42 were all significantly decreased in the hippocampus of gossypetin-treated 5xFAD mice (Fig. 2K–N). Therefore, gossypetin ameliorated behavioral deficits in 5xFAD mice by reducing not only the Aβ plaques but the overall Aβ level in the brain as well.

### Gossypetin does not affect the Aβ production pathway

Since results suggested that gossypetin reduces both Aβ plaques and Aβ monomers, we wanted to elucidate how gossypetin was able to decrease Aβ in 5xFAD mice. We hypothesized that gossypetin would affect either the Aβ production or the clearance pathway. Therefore, we first investigated whether gossypetin modulates the Aβ production pathway. Many flavonoid compounds were previously reported to decrease the activity of β-secretase, which is the rate limiting enzyme of Aβ production [6, 17]. Thus, we measured the activity of β-secretase in hippocampal lysates. Although there was increased β-secretase activity in 5xFAD mice, there was no difference in β-secretase activity between vehicle- and gossypetin-treated 5xFAD hippocampal lysates (Fig. S1 A-G). Next, we measured the mRNA and protein levels of the β-secretase and γ-secretase subunits. We confirmed that gossypetin produced no difference in protein levels of β-secretase and γ-secretase (Fig. S1, H–L) and the mRNA level of BACE1, nicastrin, APH-1, or PEN-2 (Fig. S1, M–P). Based on these results, gossypetin does not seem to affect the production of Aβ.

### scRNA-seq reveals that gossypetin boosts phagocytic activity and antigen presentation in microglia

**Fig. 3:**
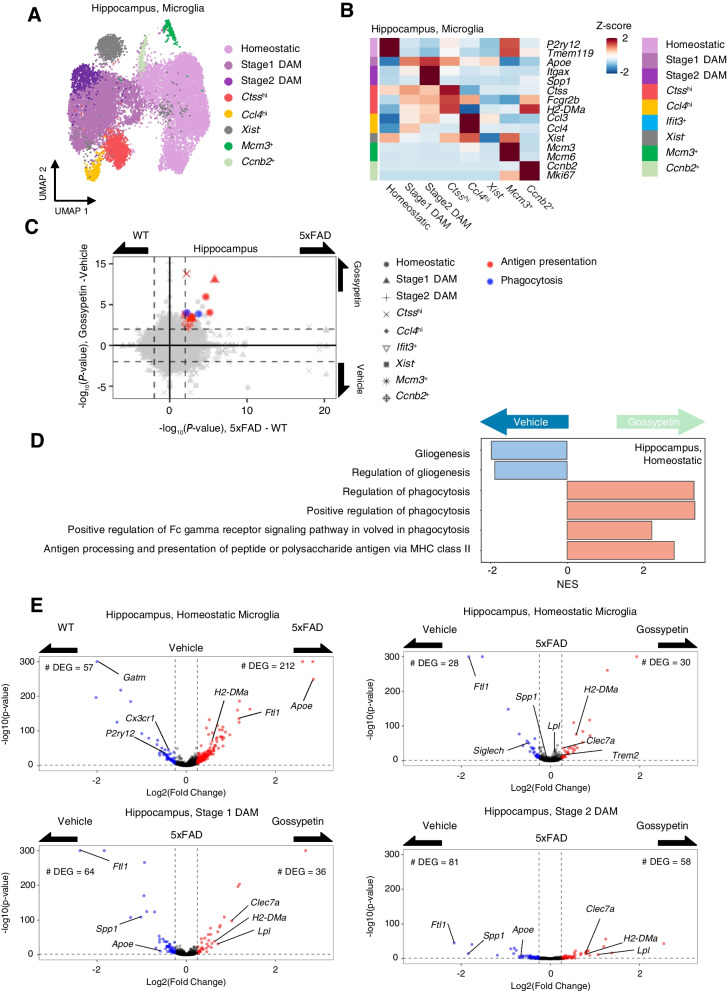
Single-cell characterization of altered transcriptome profiles of 5xFAD microglia by gossypetin administration. (**A**) Uniform manifold approximation and projection (UMAP) plots showing all microglial cells from hippocampus region. The cells are colored by their cell types. (**B**) Heatmap showing the *Z*-scores of average normalized expressions of representative DEGs for each cell type from hippocampus region. (**C**) Scatter plot showing biological processes of Gene Ontology (GOBP) terms that are upregulated or downregulated by 5xFAD or gossypetin administration for each microglial subtype from hippocampus. Among the significant (Fisher’s exact test, *p* < 0.01) terms, terms associated with antigen presentation and phagocytosis are colored by their biological keywords. (**D**) Bar plot displaying gene set enrichment analysis (GSEA)-calculated normalized enrichment score (NES) of GOBP terms for gossypetin administration condition against vehicle treatment within 5xFAD homeostatic microglia from hippocampus. (**E**) Volcano plot illustrating the differentially expressed genes selected by the comparison between wild type and 5xFAD (left-top) or vehicle and gossypetin (left-bottom, and right) cells from homeostatic microglia and disease-associated microglial population of hippocampus region. Significant (*p* < 0.05) and DAM-related genes are labeled

Because gossypetin did not have any effect on the Aβ production pathway, we thought that gossypetin might stimulate the clearance of Aβ. Glial cells, especially the microglia and the astrocytes, were reported to play a central role in modulating the Aβ clearance and degradation [5]. Therefore, we performed single-cell RNA sequencing (scRNA-seq) from both cortex and hippocampus of 5xFAD mice to see whether gossypetin modulates transcriptome of glial cells. We used the brain of one wild type (WT) mouse and two 5xFAD mice, each from the vehicle- or gossypetin-treated group for analysis. We were able to obtain 17,629 cells from the WT mice and 31,363 cells from the 5xFAD mice (Fig. S2). Graph-based unsupervised clustering after correcting the batch effects in scRNA-seq data identified *Aif1-*, *Cd68-*, *Fcgr1-*, *Itgam-*, and *Trem2*-positive clusters as microglia (Fig. S2, A and B; and Fig. S3 A). We observed that the majority of identified cells were microglia while neurons, which is the major cell type in brain tissues, were found to be depleted. This depletion is likely due to the cell dissociation process. Enzymatic dissociation at warm temperature causes relatively selective deaths of neurons and astrocytes compared to mechanical dissociation at low temperatures, establishing a cell population enriched with microglia [18–20]. We decided to focus on microglia and further subclustered the microglial population into eight subtypes in both the cortex and hippocampus microglia according to their differentially expressed genes (DEGs) and inferred cell cycle (Fig. 3A, B; and Fig. S3, A-C). Microglia from the WT mice were mostly homeostatic, while disease-associated microglia (DAM) and other microglial subpopulations were found in the 5xFAD mice (Fig. S3, B and D) [21]. We were unable to find any changes in the number or the proportion of microglial subpopulations driven by treatment of gossypetin.

We next performed gene ontology biological processes (GOBP) enrichment analysis for each microglial subtype. The treatment of gossypetin upregulated largest number of GOBP in hippocampal homeostatic microglia of 5xFAD mice. Notably, terms related to phagocytosis and antigen presentation via MHC II were significantly upregulated in gossypetin-treated 5xFAD mice. An increased trend of antigen presentation via MHC II was found in most of the hippocampal microglial subtypes, while a similar trend was found only in the homeostatic microglia and stage 1 DAM in the cortex (Fig. 3C; Fig. S4 A; and Additional file 1). We further calculated the normalized enrichment score (NES) by gene set enrichment analysis (GSEA) for hippocampal homeostatic microglia. Gossypetin-treated 5xFAD showed a decreased NES score in gliosis related gene sets (Fig. 3D; and Fig. S4 B). As we have previously mentioned, the upregulated genes in gossypetin-treated hippocampal homeostatic microglia showed high enrichment of gene sets related to phagocytosis and antigen presentation via MHC II (Fig. 3D). We also examined the individual DEGs of 5xFAD microglia affected by gossypetin treatment. Among those DEGs, *H2-DMa*, *Lpl*, and *Clec7a* were upregulated, while *Ftl1*, *Apoe*, and *Spp1* were downregulated in homeostatic and stage 1 and 2 DAM subtypes (Fig. 3E; Fig. S5 A; and Additional files 2 and 3). This result was very interesting because although *Apoe*, *Spp1*, *Lpl*, and *Clec7a* are all well-known markers for DAM, *Apoe* and *Spp1* showed completely opposite response to gossypetin treatment when compared to *Lpl* and *Clec7a* with gossypetin treatment. We calculated the DAM signature score and found a significant decrease in DAM signature from the stage 1 DAM population in the hippocampus and cortex (Fig. S5 B). Previous studies report that *Apoe* and *Spp1* promote inflammation while *Lpl* and *Clec7a* sustain phagocytic activity of microglia [22–25]. Since DAM was already reported to have high gene expression associated with phagocytosis, it was very interesting to see gossypetin-mediated increase in phagocytic genes even in DAM subtypes [21]. Taken together, our scRNA-seq results suggest that gossypetin reduced overall transcriptomic signature of DAM in various microglial subpopulations. However, gossypetin upregulated genes associated with phagocytosis, which presumably led to the reduction of Aβ by enhancing clearance through microglia and ameliorated microgliosis in 5xFAD mice.

### Gossypetin ameliorates gliosis and enhances Aβ phagocytosis of microglia

Based on the results from scRNA-seq, we investigated whether the changes in GOBP terms by gossypetin could be reproduced in 5xFAD mice. First, we investigated whether gliosis was ameliorated in 5xFAD mice through immunohistochemistry since gliosis is a major hallmark of AD. Since gliosis occurs in both microglia and astrocyte in 5xFAD, we stained microglial marker Iba-1 and astrocyte marker GFAP to measure gliosis. We observed a dramatic decrease of gliosis in both the microglia and the astrocytes in the hippocampus and cortex of 5xFAD mice treated with gossypetin (Fig. S6).

**Fig. 4:**
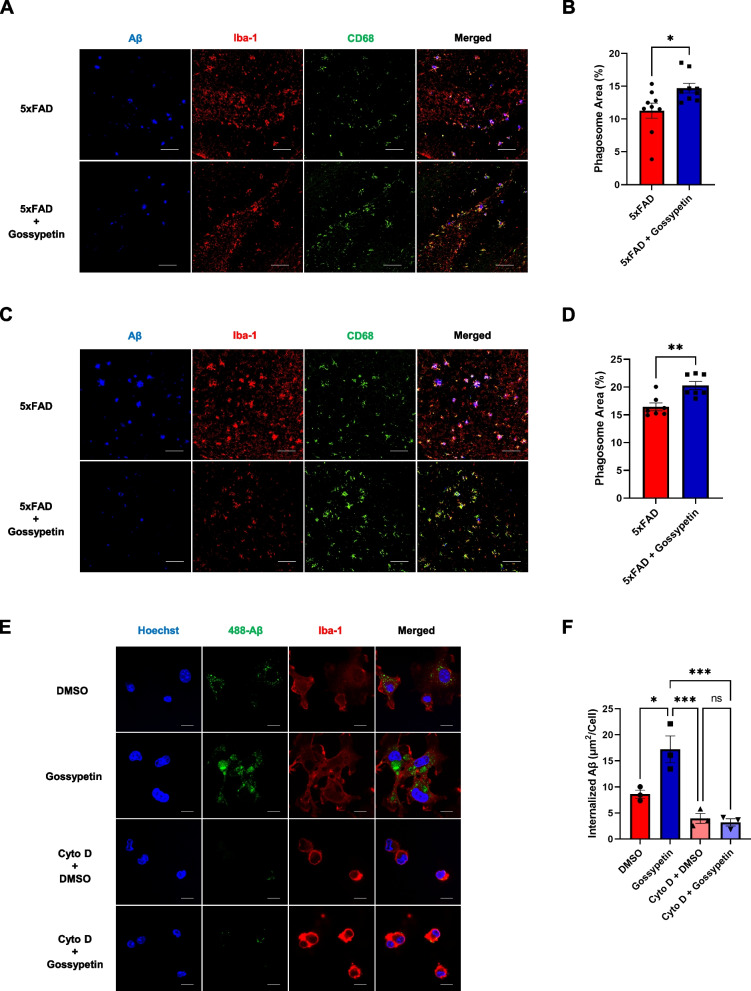
Gossypetin enhances microglial phagocytosis against Aβ. **A**–**D** Representative images of Aβ (6E10), Iba-1, and CD68 staining in hippocampus (**A**) and cortex of 5xFAD and gossypetin treated 5xFAD mice (**C**). Scale bar corresponds to 100μm. Bar graph represents quantification of phagosome formation (colocalization of Iba-1 and CD68) in hippocampus of 5xFAD treated with vehicle or gossypetin (*n* = 9 per group, 3~9 slices per brain slice) (**B**) and cortex (*n* = 7 per group, 3 slices per brain) (**D**). **E**, **F** Representative images of primary microglia treated with 488-Aβ and stained with Hoechst and Iba-1. Gossypetin (25μM) was pretreated for 24 h before 488-Aβ (250nM) treatment. Scale bar corresponds to 10 μm. **E** Bar graph represents quantification of area of internalized 488-Aβ in microglia (*n* = 3 per group, 50~104 cells per sample). Cytochalasin D (Cyto D), which is a phagocytosis inhibitor, was treated as a negative control (**F**). The error bars represent the mean ± SEM. ****p < 0.0001, ***p < 0.001, **p < 0.01, *p < 0.05, ns = not significant, two-way ANOVA followed by Tukey’s multiple comparisons test (**F**), Student’s *t* test (**B**, **D**)

Then, we evaluated whether phagocytic activity of microglia was increased as well, like the result from scRNA-seq suggested. We co-stained hippocampus and cortex with Iba-1 and CD68, which are microglial and lysosomal markers respectively. We observed increased phagosome formation in microglia at the hippocampus and cortex of 5xFAD mice treated with gossypetin (Fig. 4A–D). This indicates that gossypetin increased the phagocytic activity of microglia in 5xFAD mice. To further confirm whether gossypetin was able to enhance the phagocytic activity of microglia, we cultured primary microglia and BV2 mouse microglial cell line. We treated these cells with recombinant 488-tagged fluorescent Aβ (488-Aβ) that emits fluorescence only when it is accumulated inside a cell. Primary microglia and BV2 pretreated with gossypetin showed increased uptake of 488-Aβ as they showed enhanced fluorescence in the cell (Fig. 4E, F; Fig. S7 A and B). To confirm that the increased uptake of 488-Aβ in primary microglia was caused by phagocytosis, we applied cytochalasin D (Cyto D), which is a phagocytosis inhibitor. When Cyto D inhibited phagocytosis, only minimal fluorescence was detected in primary microglia, indicating that gossypetin could increase the phagocytosis of microglia (Fig. 4E, F). Since we have confirmed that gossypetin increases uptake of Aβ through phagocytosis, we wanted to know whether phagocytic dynamics and overall phagocytic capacity of microglia were affected by gossypetin treatment. We conducted live cell imaging to observe the phagocytic activity of BV2 cell during 488-Aβ uptake. We found that gossypetin treatment induced microglia to uptake 488-Aβ more quickly. Also, overall capacity for Aβ uptake was increased in BV2 cells when treated with gossypetin (Fig. S7 C; Additional files 4, and 5). Therefore, we confirmed that gossypetin mediates Aβ clearance by increasing the phagocytic dynamics and capacity of microglia.

### Gossypetin increases expression of MHC II in microglia

**Fig. 5:**
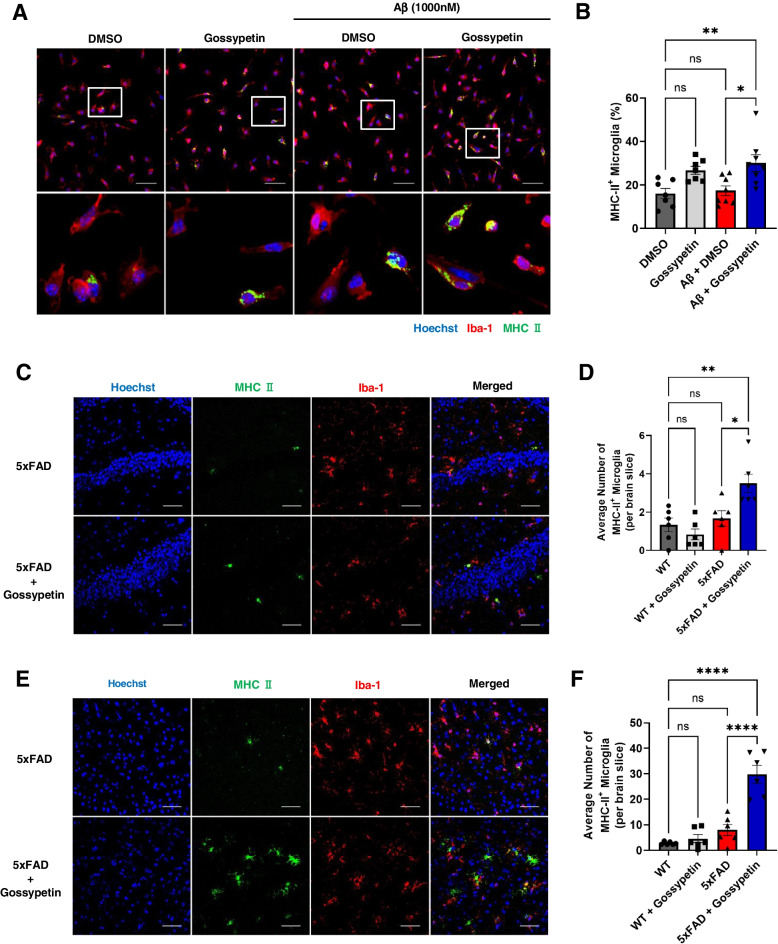
Gossypetin increases MHC II^+^ microglia. **A**, **B** Representative images of primary microglia stained with Hoechst, Iba-1, and MHC II. Primary microglia were pretreated with gossypetin (25 μM) for 24 h and treated with recombinant Aβ (1 μM). Scale bar corresponds to 50μm (**A**). Bar graph represents quantification of MHC II^+^ microglia percentage (*n* = 6~7, 88~203 cells per set) (**B**). **C**, **D** Representative images of hippocampus stained with Hoechst, Iba-1, and MHC II in 5xFAD and gossypetin treated 5xFAD mice. Scale bar corresponds to 50μm (**C**). Average number of MHC II^+^ microglia were counted (*n* = 6 per group, 3 slices per brain) (**D**). **E**, **F** Representative images of cortex stained with Hoechst, Iba-1, and MHC II in 5xFAD treated with vehicle or gossypetin. Scale bar corresponds to 50μm (**E**). Bar graph represents average number of MHC II^+^ microglia that were counted (*n* = 6 per group, 3 slices per brain) (**F**). The error bars represent the mean ± SEM. ****p < 0.0001, **p < 0.01, *p < 0.05, ns = not significant, two-way ANOVA followed by Tukey’s multiple comparisons test

Because our scRNA-seq results also showed increased antigen presentation via MHC II in microglia due to gossypetin treatment, we wanted to determine whether we could observe such trends in primary microglia and brain tissues. In primary microglia culture, we observed a further increase in the number of MHC II^+^ microglia in gossypetin-treated primary microglia after Aβ treatment (Fig. 5A, B). Also, we found increased number of MHC II^+^ microglia in the cortex and hippocampus of gossypetin-treated 5xFAD mice (Fig. 5C–F). This confirmed the scRNA-seq data that gossypetin increases MHC II expression in microglia.

## Discussion

In this study, we confirmed the therapeutic efficacy of gossypetin against AD in 5xFAD mice based on the recovery of spatial learning and memory in Y-maze and MWM test. Gossypetin ameliorated AD progression by decreasing various species of Aβ. We confirmed that the decrease in Aβ level by gossypetin was not caused by the modulation of the Aβ production pathway but by enhancing the phagocytosis of microglia. It was previously reported that gossypetin inhibits the aggregation of Aβ and tau in vitro [12]. Since gossypetin and other flavonoid compounds possess aromatic ring in its chemical structure, it is possible that gossypetin may hinder aggregation between Aβ by competitive π-π stacking interaction with hydrophobic residue of Aβ (e.g. -FF- motif) [26]. Therefore, not only does it induce the clearance of Aβ by microglia, gossypetin may also contribute to the inhibition of Aβ aggregation, which may help the process of Aβ turnover through various clearance pathway and improve Aβ pathology.

Since previous report suggested that gossypetin can inhibit aggregation of Aβ and tau in vitro [12], there is a possibility that gossypetin may have a role in tau pathology as well. Tau is one of the most well-known hallmarks of AD along with Aβ. Hyperphosphorylation of tau causes conformational change that allows tau to aggregate. Aggregation of tau leads to the formation of paired helical filaments and neurofibrillary tangles. These neurofibrillary tangles further cause the dysfunction of axonal stability and impairment of neurotransmission resulting in death of neuronal cells that leads to functional deficits of the brain [27]. If gossypetin can prevent the formation of neurofibrillary tangles by inhibiting aggregation of tau, there is a high chance that gossypetin may ameliorate tau mediated pathology of AD. Additionally, our study showed that various forms of Aβ were reduced with gossypetin treatment in 5xFAD mice. Since Aβ can directly induce hyperphosphorylation of tau [28], we can hypothesize that gossypetin can effectively prevent aggregation of tau in both direct and indirect ways.

scRNA-seq allowed us to identify various subclusters of microglia apart from well-recognized homeostatic microglia and DAMs. We have labeled other subclusters of microglia with one of the DEGs which is uniquely expressed in each subpopulation. We named these identified microglial subclusters as *Ctss*^hi^, *Ccl4*^hi^, *Ifit3*^+^, *Xist*^-^, *Mcm3*^+^, and *Ccnb2*^+^ microglia. Although we have not addressed specific role of gossypetin in these subpopulations due to lack of evidence and small number of each population, we were able to get some hints from analyzing their GOBP terms or comparing microglial scRNA-seq result from other studies, which allowed us to predict their functions. *Ctss*^hi^ microglia showed highest expression of genes associated with phagocytosis and MHC II demonstrating a subpopulation with very strong phagocytic activity. Recent literatures suggested protective role of *Xist* downregulation in microglia. It reported that *Xist* knockdown led to microglial autophagy-mediated NLRP3 inactivation in LPS-induced BV2 cells [29], which implicates a protective role of *Xist*^-^ microglial subpopulation in 5xFAD mice. For *Ccl4*^hi^, *Ifit3*^+^, *Mcm3*^+^ and *Ccnb2*^+^ microglia, we have found that these microglial subpopulations have already been introduced in a previous report. Hammond et al. have conducted scRNA-seq with mice brains at different ages and injury state. By comparing our scRNA-seq data with their results, we have found that *Ccl4*^hi^ microglia showed resemblance to cluster 8. Cluster 8 was reported to be found in two different conditions: aging and injury. Hammond et al. discussed that *Ccl4*^+^ population could be a specific subpopulation that is primed to produce an inflammatory response. On the other hand, *Mcm3*^+^ and *Ccnb2*^+^ microglia were found to be corresponding to cluster 2a and 2c, respectively. These microglia were reported to be proliferative and most populated at E14.5. Therefore, they are predicted to be participating in developmental pathways. Lastly *Ifit3*^+^ microglia corresponded to cluster 9. This subpopulation of microglia was predominantly found to be expressed in the focal white matter injury [30]. However, their role in the injured site was not discussed. Therefore, further study is needed to understand the roles played by these subclusters of microglia.

Through scRNA-seq, existence of a microglial subpopulation called disease-associated microglia (DAM) or neurodegenerative microglia (MGnD) has been reported in AD models [21, 22]. DAM were initially reported to be beneficial in AD because of their high expression of genes related to phagocytosis and their localization close to Aβ plaques [21]. However, accumulating reports have rather suggested that MGnD is detrimental in AD by increasing TREM2-APOE signaling, which leads to neuroinflammation [22]. DAM or MGnD is well characterized by downregulated genes associated with homeostatic microglia while having high expression of genes such as *Apoe*, *Trem2*, *Spp1*, *Clec7a*, *Lpl*, *Gpnmb*, *Itgax*, and *Axl* [21, 22]. However, gossypetin treatment produced different changes in the expression of these DAM marker genes. Stage 1 and 2 DAM of gossypetin treated 5xFAD showed significantly downregulated expression of *Apoe* and *Spp1*, while upregulated expression of *Clec7a* and *Lpl*. We also found that gossypetin decreased the DAM signature in stage 1 DAM, although there was no difference in actual cell population. *Apoe*, along with *Trem2*, is considered a “switch” that turns homeostatic microglia to MGnD [22]. It was reported that *Apoe* suppresses TGFβ signaling and mediates neuroinflammation [22]. Knockout of *Apoe* restores microglia from MGnD to homeostatic microglia and decreases Tau pathology [22, 31]. Similarly, *Spp1* is a proinflammatory cytokine that is involved in various neurodegenerative diseases including AD [23]. These findings suggest a possibility that *Apoe* and *Spp1* may drive inflammatory and neurodegenerative phenotypes in microglia, and it could be suppressed by gossypetin treatment.

Lipid droplet accumulation in microglia was one of the key features observed initially by Alois Alzheimer about 100 years ago. These “lipid droplet accumulated microglia” (LDAM) were recently found to have decreased phagocytic activity [32, 33]. According to previous reports, *Lpl* decreases lipid droplet accumulation in microglia [34]. Moreover, *Lpl*-deficient microglia showed decreased phagocytic capacity, which corresponds to results about LDAM [24]. We found that *Clec7a* was also upregulated by gossypetin treatment. Since *Clec7a* was also reported to support Aβ phagocytosis, it is possible that increased expression of *Lpl*, and *Clec7a* in microglial subpopulations by gossypetin treatment may have contributed to increasing their phagocytic activity [25]. Additionally, other well-known hallmark of AD is increased ROS in the brain. Elevated ROS induces the formation of lipids in neurons and these lipids are transferred to glial cells and form lipid droplets [35, 36]. Also ROS is reported to increase through various pathways such as Aβ accumulation, mitochondrial dysfunction, and gliosis in AD [37–39]. However, we have shown that gossypetin could efficiently ameliorate the gliosis of both microglia and astrocytes. Also gossypetin is well known for its strong antioxidant capacity [10]. These results all suggest that gossypetin could inhibit harmful phenotypes of microglia in AD while enhancing their protective role. Further research is necessary to thoroughly investigate how gossypetin induces these changes in microglial transcriptome. Confirmation of these findings could establish a novel therapy by achieving precise and optimal modulation of microglia for AD treatment.

ScRNA-seq revealed that gossypetin modulates the transcriptomic profiles of microglia. Microglia showed increased expression of MHC II upon the treatment with gossypetin. MHC II is expressed in antigen-presenting cells and controls the activation of CD4 T cells. Although the role of MHC II in microglia, especially in neurodegenerative diseases, is not well known, a recent report revealed that 5xFAD/MHC II^−/−^ mice showed aggravated Aβ pathology and decreased phagocytic activity of microglia. Moreover, 5xFAD/MHC II^−/−^ mice were irresponsive to Aβ-specific CD4 T cell injection, which improved AD pathology in normal 5xFAD mice [40]. These results correspond to another report, which showed that the induction of MHC II^+^ microglia increased phagocytic activity by the treatment with IL-33 [41]. Taken together, these reports suggest that MHC II has a positive role in microglial phagocytosis and protection against AD. Although further research is needed, the protective role of microglia as antigen-presenting cells and their interactions with T cells are being investigated in neurodegenerative diseases [42]. Additionally, recent studies have highlighted the role of the meningeal lymphatic system in the clearance of Aβ in AD [43, 44]. Since lymphatic system is deeply related to interaction between antigen presenting cells and T cells, it is possible that the increase in the expression of MHC II in microglia by gossypetin suggest more than what we currently understand about microglia and immune system in CNS. Therefore, it will be interesting to elucidate how the adaptive immune system is involved in the maintenance of brain homeostasis and response against neurodegenerative diseases.

This study had few limitations. As we have mentioned previously, we have enzymatically dissociated the brain tissues at warm temperature. Although this protocol allowed us to acquire sufficient number of microglia for scRNA-seq, we had to suffer depletion of neurons and astrocytes during the dissociation process. Because of that, we did not have sufficient number of neurons and astrocytes to analyze the effect of gossypetin in these cell types in 5xFAD mice. Therefore, in our future study, we need to dissociate the brain tissues through mechanical dissociation at cold temperature in order to confirm the role of gossypetin in neurons and astrocytes. In our study, we have mentioned that the genes associated with phagocytosis were upregulated through gossypetin in various microglial subclusters including stage 1 DAM. However, despite the upregulation of phagocytosis genes, overall DAM signature score was decreased in stage 1 DAM signature by gossypetin treatment. We hoped to confirm this finding through histological analysis. However, according to previous report, all the markers of stage 1 DAM were also upregulated in stage 2 DAM [21]. Therefore, due to the lack of understanding about stage 1 DAM, we were unable to find a way to specifically target the stage 1 DAM. Further study needs to be followed after we gain more insights about the stage 1 DAM.

## Conclusion

Collectively, we demonstrated that gossypetin rescued spatial learning and memory of 5xFAD mice by reducing various forms of Aβ. With scRNA-seq, we have observed novel pattern of microglial transcriptomic modulation through gossypetin. Gossypetin treatment reduced various AD hallmarks such as microgliosis and astrogliosis. Lastly, gossypetin increasing phagocytic dynamics and capacity of microglia against Aβ. Our work presents gossypetin as novel therapeutic candidate against AD.

## Methods

### Mouse

5xFAD (Tg6799, Jackson Laboratory) male mice were mated with B6/SJL (#100012, Jackson Laboratory) female mice and the genotypes of the offspring were determined by PCR analysis of tail cut samples. The non-transgenic female littermates were used as wild-type control animals and only female 5xFAD mice were used in this study.

### Intragastric administration

Eight-week-old wild-type (WT) and 5xFAD (Tg) mice were administered with 10 mg/kg of Gossypetin (Boc Science) in 150 μl every day for 13 weeks through intragastric administration. Mice of control groups (WT-Vehicle, 5xFAD-Vehicle) were administered the same volume of vehicle (10mM Tris pH7.5, 200mM NaCl with 1% DMSO or 1% carboxymethyl cellulose with 1% DMSO).

### Y-maze test

The Y-maze apparatus has three V-shaped arms (40 × 3 × 12 cm) at 120° angles from each other. To test spontaneous alternation, a mouse was placed at the end of an arm and allowed to freely explore the Y-maze during 8 min under the dim light conditions (50 lux). Alternation was defined as consecutive entry in three different arms. The total number and the sequence of individual arm visits were recorded, and alternation ratio was calculated [45, 46].

### Morris water maze (MWM)

The water maze tank (120 cm diameter) was filled with water and white non-toxic tempera paint was diluted to make the water opaque. Four different visual cues were placed each cardinal points. The water temperature was set at 22 ± 1 °C before the everyday trial. Mice were habituated to the behavior test room for at least 15 min before starting the test. Three training trials (60 s each, 1 h intervals) were performed per day for five consecutive days. For each training trial, each mouse was allowed to remain on the platform for 15s after finding the platform and was removed from the maze and returned to its home cage. If the mouse did not find the platform within 60s, it was guided to the platform and returned to its home cage after 15s. A probe trial was performed on the sixth day. The trajectories of swim were recorded and analyzed with a video tracking system (SMART v2.5 and SMART v3.0, Panlab, RRID:SCR_002852) [45, 46].

### Tissue collection

Mice were treated with CO_2_ for them to lose consciousness. Then, mice were transcardially perfused with ice-cold phosphate buffer saline (PBS, pH 7.4). After decapitation, skull was removed to collect the brain. Brains were cut into two hemispheres. One hemisphere was fixed in 4% paraformaldehyde (PFA) for overnight at 4 °C and placed into 30% sucrose in PBS at 4 °C until it settles down. Then it was frozen in O.C.T compound (Sakura, 4583) and kept at – 80 °C for further histological analysis. For the other brain hemisphere, it was further dissected to collect hippocampus and cortex. Collected tissues were quickly frozen in liquid nitrogen and kept at – 80 °C for further biochemical analysis. Frozen hippocampus and cortex were mechanically crushed to make them into powder. Powdered hippocampus and cortex were aliquoted for them to be used in protein and RNA extraction [45].

### Protein extraction

To divide soluble and insoluble protein extraction in hippocampus and cortex, two steps of serial extraction protocol was used. To prepare the soluble extract, hippocampal and cortical samples were weighed and homogenized at 100 mg/ml in cold TBS (50 mM Tris pH 7.5, 150 mM NaCl) with protease inhibitor cocktail (Thermo, A32953). The TBS homogenates were centrifuged at 15,000 rpm for 1 h at 4 °C. The supernatants were stored at − 80 °C until further use. The pellets were reserved for the extraction of insoluble proteins. To prepare the insoluble extract, the previously obtained pellets were dissolved in 5M guanidine buffer (5M guanidine HCl, 150mM NaCl, pH 7.5) and vortexed until all pellets got dissolved completely. After centrifugation at 15,000 rpm for 1 hrat 4 °C, the supernatants were stored at − 80 °C until further use [45].

### Western blot analysis

Soluble fraction of brain lysates was used for western blot. Fifteen to thirty micrograms of brain lysates were separated by SDS-PAGE on SDS gels (5% stacking, 10~15% resolving) and transferred to nitrocellulose membranes (110V constant for 90min, wet transfer). Some of the protein samples were separated on Bolt^TM^ 4–12% Bis-Tris Plus gels with MES running buffer (200 V constant, for 25 min) and transferred to nitrocellulose membranes using Bio-Rad wet transfer system (100 V constant for 1 h). The membranes were blocked with 5% skim milk in TBST (200mM Tris, 3M NaCl, 1% TWEEN-20, pH 7.6). The membranes were labeled with primary antibodies (anti-GAPDH, 1:1000, Bethyl #A300-641A, RRID:AB_513619; anti-PEN2, 1:500 ,Cell Signaling #8598, RRID:AB_11127393; anti-APH-1, 1:100, Thermo Fisher #PA1-2010, RRID:AB_2227105; anti-Nicastrin, 1:1000, Cell Signaling #5665, RRID:AB_10694688; anti-BACE1, 1:1000, Cell Signaling #5606, RRID:AB_1903900; anti-β-amyloid, 1:100, BioLegend #803002, RRID:AB_2564654), for overnight at 4°C and then labeled with the HRP-conjugated secondary antibodies (anti-Rabbit IgG HRP-conjugate, 1:5,000, Promega #W4011, RRID:AB_430833; anti-mouse IgG Secondary antibody HRP, 1:5000, Thermo Fisher #31430, RRID:AB_228307). Chemiluminescence images were captured by ImageQuant LAS 4000 (Fuji, RRID:SCR_014246).

### Dot blot assay

Soluble hippocampal lysates (4 μg) were diluted in 100μl of TBS. The diluents were loaded on nitrocellulose membrane using the Bio-Dot® microfiltration apparatus (BIO-RAD) and filtered under vacuum. The membrane was washed with TBST and followed by blocking with 10% skim milk. Primary antibody, anti-oligomer A11 (1:2000, Thermo Fisher #AHB0052, RRID:AB_2536236), and HRP-conjugated secondary antibody (anti-Rabbit IgG HRP-conjugate, 1:5,000, Promega #W4011, RRID:AB_430833) were used for detecting soluble oligomers. Chemiluminescence image was captured by ImageQuant LAS 4000 (Fuji, RRID:SCR_014246).

### ELISA

Aβ40 and Aβ42 sandwich ELISA kits (Invitrogen, KHB3481, KHB3442) were used to analyze the soluble and insoluble Aβ levels. The soluble extracts were diluted in the ratio of 1:50 and 1:200 for Aβ40 and Aβ42, respectively, and the insoluble extracts were diluted in the ratio of 1:5,000 and 1:20,000 for Aβ40 and Aβ42, respectively. The extracts were diluted with ELISA diluent buffer. The assay was performed as the manufacturer’s protocol. Synthetic Aβ40 and Aβ42 peptides in the assay kit were used for creating standard curve.

### Acetylcholinesterase inhibition assay

Acetylcholinesterase inhibition assay was performed with acetylcholinesterase inhibitor screening kit (colorimetric, Biovision, Milpitas, CA, USA) according to manufacturer’s protocol. One hundred micrometers of gossypetin dissolved in DMSO was used for acetylcholinesterase inhibition assay.

### Beta-secretase activity assay

Beta-secretase Activity Assay Kit (Fluorometric) (ab65357) was used to analyze activity of beta secretases in mouse hippocampal lysate. Assay was performed as manufacturer’s protocol.

### Immunohistochemistry

Coronal sections (10μm) of mouse brains were cut using a cryostat (Leica CM1850) and stored at – 20 °C until use. Upon use, brain sections were washed three times with PBS and blocked with blocking buffer (5% Fetal Bovine Serum (FBS) + 3% Bovine Serum Albumin (BSA) + 0.3% Triton X-100 in PBS) for 1 h at room temperature (RT). Brain sections were then incubated with primary antibodies (anti-β-amyloid, 1:100, BioLegend #803002, RRID:AB_2564654; anti-Iba-1, 1:200, Wako #019-19741, RRID:AB_839504; anti-GFAP, 1:200, Abcam #ab7260, RRID:AB_305808; anti-CD68, 1:200, Abcam #ab53444, RRID:AB_869007; anti-MHC class 2, 1:100, Thermo Fisher #14-5321-82, RRID:AB_467561) in blocking buffer overnight at 4 °C. Sections were washed three times with PBS and treated with secondary antibody (Anti-mouse IgG Alexa Fluor 405, 1:100, Thermo Fisher #A-31553, RRID:AB_221604; Anti-mouse IgG Alexa Fluor 488, 1:500, Thermo Fisher #A-11001, RRID:AB_2534069; Anti-Rabbit IgG Alexa Fluor 488, 1:200, Thermo Fisher #A-11008, RRID:AB_143165; Anti-Rabbit IgG Alexa Fluor 594, 1:200, Thermo Fisher #A-11012, RRID:AB_2534079; Anti-Rat IgG Alexa Fluor 594, 1:500, Thermo Fisher #A-11007, RRID:AB_10561522; Anti-Rat IgG Alexa Fluor 488, 1:200, Thermo Fisher #A-11006, RRID:AB_2534074) antibody in blocking buffer for 1h protected from light. Brain sections were then washed three times again, and Hoechst 33342 (10μg/ml) diluted in PBS (1:2500) was stained for 10 min protected from light. Lastly, sections were washed again with PBS three times and mounted with fluorescence mounting medium (Dako, S3023). Images were captured with ZEISS Axioplan 2 fluorescent microscope (RRID:SCR_020918) and Olympus confocal microscope (FV3000, RRID:SCR_017015). The GFAP or Iba-1 positive cell regions, number of Aβ (6E10) plaques and area, and phagosomes in prefrontal cortex and dentate gyrus of hippocampus were analyzed by Image J (Fiji) software (RRID:SCR_002285). Stained images were opened with Image J. Dentate gyrus and cortical regions were specifically selected in each image with “freehand selection” tool. After selecting the area for analysis, images were made into 8-bit. Then, fluorescence positive area was visualized through “threshold” tool and quantified with “Analyze Particle” tool [45].

### Primary microglia culture

Three-day-old ICR mice were used for primary microglial culture. Mice were anesthetized in cold ice and decapitated. After removal of skull and meninges, cortex and hippocampus were extracted and dipped in HBSS in 15 ml conical tube. Tissue was mechanically dissociated through pipetting. Then, trypsin was added to tissue in 1:1 ratio (4 ml each) with HBSS and incubated in 37 °C water bath for 15 min. During incubation, conical tube was inverted every 3 min. Then, 2 ml of FBS was added and centrifugated at 700 rpm for 15 min. Supernatants were removed, and tissue was dissociated and cultured with primary culture medium (FBS 5 ml, Horse serum 5 ml, penicillin-streptomycin 500 μl, glutaMAX 500 μl, 25 ng m-CSF, DMEM F12 in 50 ml) in cell culture flask (SPL life science) with 16 ml of media in each flask. Media was changed 2~3 days after the incubation. Then, half of the media was changed every 2 days until harvest. Whole media was changed a day before harvest and primary microglia was harvested by shaking culture flasks in 260 rpm for 3h. Then, media was collected and centrifuged at 700 rpm for 15 min. Then, cell pellets were harvested and cultured on glass chip on 12~24 well plate and used for analysis [46].

### Phagocytosis assay and immunocytochemistry

Primary microglia and BV2 cells grown on glass chips in 12 or 24-well plate were used for phagocytosis assay. For live cell imaging, BV2 cells were seeded on confocal dish. Upon seeding microglial cells on glass chips, gossypetin (25 μM) or equal volume of DMSO was treated to each well and incubated for 24 h. Before the treatment of 488-Aβ, some wells were treated with cytochalasin D (20 μM) for 30 min before 488-Aβ treatment. After all the media were removed, cells were treated with media containing 24 h preincubated 488-Aβ (250 nM) for 1 h. For wells that were pre-treated with cytochalasin D, media with 488-Aβ were added with cytochalasin D. Cells were washed once with warm PBS and were fixed with 4% PFA for 40min at RT. After washing three times, cells were treated with 0.5% NP-40 in PBS for 30 min and washed once. Cells were incubated with blocking solution (5% FBS, 2.5% BSA in PBS) for 2 h at RT. Then, primary antibody (anti-Iba-1, 1:200, Wako #019-19741, RRID:AB_839504; anti-MHC class 2, 1:100, Thermo Fisher #14-5321-82, RRID:AB_467561) was treated and incubated at 4 °C for overnight or 4h at room temperature. Cells were washed three times and treated with secondary antibody at RT for 2h. After washing three times, cells were stained with Hoechst 33342 (10 μg/ml) diluted in PBS (1:2500) for 10 min and washed twice additionally. Cells were finally mounted with fluorescence mounting medium (Dako, S3023). For live cell imaging, after the media was exchanged with media containing preincubated 488-Aβ, live cell images were taken every 2 min for 1 h. Images were captured using Olympus confocal microscope (FV3000, RRID:SCR_017015) and analyzed by Image J (Fiji) software (RRID:SCR_002285). After selecting Iba-1 positive area with “create selection” tool, selected area was applied to 488-Aβ positive area to measure 488-Aβ that had been phagocytosed by microglia. After measuring the 488-Aβ positive area, it was divided by number of microglial cells. Number of microglial cells were measured by counting the nucleus stained by Hoechst. “Multi-point” tool was used to count the number of nuclei.

### RNA isolation and quantitative real-time polymerase chain reaction

RNA was extracted from hippocampus and cortex with TRI-solution (Bio Science Technology, TS200-001). Seven hundred microliters of TRI solution was added to each tissue and homogenized with pestle. Then, each sample was vortexed, and 140 μl of chloroform was added and vortexed. The mixture was incubated at RT for 10 min and centrifuged at 15,000 rpm in 4 °C for 10min. Three hundred thirty microliters of supernatants were transferred to new tube and mixed with equal volume of isopropanol and vortexed and incubated for 10 min in ice. Samples were centrifuged at 15,000 rpm in 4 °C for 10 min. Then, RNA pellets were washed with 100 μl of 75% ethanol. Then, samples were centrifuged in 15,000 rpm at 4 °C for 2 min. Supernatants were carefully discarded, and RNA pellets are dissolved in 20~60 μl of DEPC water. Concentration of RNA was calculated with NanoDrop 2000 spectrophotometer (Thermo Scientific, RRID:SCR_018042) and processed into cDNA through RT-PCR using ImProm-II reverse transcription system (Promega). qRT-PCR was performed using FastStart Universal SYBR Green Master (Roche, 04913914001) using following primers: *BACE1* forward: AGAGGCAGCTTTGTGGAGAT; *BACE1* reverse: CTGGTAGTAGCGATGCAGGA; *NCSTN* forward: GACTACATTGGCAGCTCACG; *NCSTN* reverse: AGACATGGGATCTGTGTGCA; *aph1* forward: TGACAGACCGATCAGATGCA; *aph1* reverse: AAGCCCTCATCTGCCTTCTT; *PSENEN* forward: GAGAAGTTGAACCTGTGCCG; *PSENEN* reverse: ATCACCCAGAAGAGGAAGCC; *GAPDH* forward: AAATGGTGAAGGTCGGTGTG; GAPDH reverse: TGAAGGGGTCGTTGATGG. Relative expression was quantified using the ΔC_t_ method.

### Sample preparation from mouse brain for single-cell RNA sequencing

Hippocampus and cortex were dissected from mouse brain as mentioned above. Tissues were chopped on ice and washed with cold HABG medium (Hibernate A medium supplemented with B27 and glutamine) [47]. Tissues were incubated in papain dissociation buffer (Hibernate without calcium medium with 20U/ml papain, 1 mM L-cysteine, 0.5mM EDTA) at 30 °C for 30 min with mild shaking at 300 rpm. Tissues were washed with HABG medium and triturated using fire-polished Pasteur pipets. The cell suspension was filtered by 70 μm strainer. Debris were removed using Debris Removal Solution (130-109-398, Miltenyi Biotec), and red blood cells were lysed using lysis buffer (555899, BD Biosciences). To minimize the artificial transcription changes during tissue dissociation, actinomycin D was added during trituration (3 μM) and incubation with papain (45 μM) [48]. After staining with propidium iodide, live cells were sorted using FACS Aria III (BD biosciences, RRID:SCR_018091).

### scRNA-seq

Libraries for Single cell RNA seq were generated using Chromium single cell 3′ library & Gel Bead Kit v3 (PN-1000092, 10X Genomics), Chromium Single Cell B Chip kit (PN-1000074, 10X Genomics). Briefly, cells were mixed with reverse transcription (RT) reaction reagent and loaded onto the B chip aiming for 6000 captured cells per a channel. After RT reaction, gel bead-in-emulsions were transferred to tubes and performed RT reaction using thermal cycler (C1000 Touch, Bio-Rad, RRID:SCR_019688). cDNA was purified using Dynabead MyOne SILANE (Thermofisher) and further amplified. Subsequent steps for library construction were performed by the manufacturer’s instruction provided. Quality of the amplified cDNA and final libraries were monitored by Bioanalyzer (Agilent, RRID:SCR_019715). Libraries were sequenced with a 2 × 100 bp paired-end protocol on a Novaseq S4 platform from Illumina to generate minimum 20,000 read pairs per cell.

### scRNA-seq data preprocessing

Raw fastq files were processed using the Cell Ranger pipeline (v3.1.0, RRID:SCR_017344). The cDNA sequences were mapped to the mouse genome (GRCm38) using the STAR (v2.5.1b, RRID:SCR_004463) aligner with the GRCm38.97 GTF file [49]. A gene-by-cell count matrix was generated with default parameters. To remove the putative empty droplets during the single-cell capturing, we used EmptyDrops function of DropletUtils (v1.8.0) R package with FDR < 0.01 [50]. Low-quality cells with less than 3.0 total log10-scaled UMI count and with more than 25% of UMIs assigned to mitochondrial genes were excluded, where the thresholds were determined by visually inspecting outliers in the PCA plot on the quality control metrics using the calculateQCMetrics function of the scater (v1.16.1, RRID:SCR_015954) R package [51]. To remove cell-specific biases, cells were clustered using the quickCluster function of the scran (v1.16.0, RRID:SCR_016944) R package with default parameters and cell-specific size factors were computed using the computeSumFactors function of the same package [52]. The aggregated gene-by-cell count matrix across samples was normalized by dividing the raw UMI counts by cell-specific size factors. The normalized counts were then log2-transformed by adding a pseudo-count of 1. We defined highly variable genes (HVGs) with respect to biological variability using the decomposeVar and the getTopHVGs function of the scran package (RRID:SCR_016944), with the parameter of fdr.threshold < 0.05.

To remove undesired technical variables among the samples, we first split the aggregated scRNA-seq data by the sample ID. Then, each sample were normalized again using NormalizeData function of Seurat (v3.2.0, RRID:SCR_016341) R package, and 2000 HVGs for it were selected using FindVariableGenes function of the same package. The anchors for integrating the samples were identified on first 15 PCs of 2000 HVGs using FindIntegrationAnchors function of the same package; then, the samples were combined with these anchors using IntegrateData function. The *k*-nearest neighbor graph was computed with FindNeighbors function on first 15 PCs and used for computing clusters using FindClusters function with resolution = 0.1. The 15 PCs were used for the calculation of UMAP using RunUMAP function of Seurat R package. For the 13 identified clusters, cell types were annotated manually based on known marker genes.

Microglial populations were separated from the scRNA-seq data; then, we divided it by sampling region (cortex and hippocampus) of the data. After the removal of outlier cells, the single-cell transcriptome for each region were batch-corrected then re-grouped into N and M clusters respectively, on the 15 PCs of 2000 HVGs using the same method as above. The microglia subtypes were annotated based on their major markers, DEGs and cell cycle. Three cell types among them were further re-named as homeostatic microglia, stage 1 DAM, and stage 2 DAM as they were previously identified.

We identified differentially expressed genes (DEGs) between the 5xFAD and WT, and gossypetin and vehicle condition, respectively for each microglia type using the limma (v3.44.3, RRID:SCR_010943) R package with *P* < 0.05 and absolute value of log_2_FC > 0.25 [53]. To specify enriched biological processes in DEGs between each condition for each cell type, significantly upregulated or downregulated GO biological process (GOBP) terms (*P* < 0.01) were selected using the topGO (v2.40.0, RRID:SCR_014798) R package with the org.Mm.eg.db (v3.11.4) annotation data package. Further GSEA were done for the calculation of normalized enrichment score using fgsea (v1.14.0) R package. DAM signature scores were calculated using AddModuleScore function of Seurat R package; then, the difference between 5xFAD/Vehicle and 5xFAD/Gossypetin condition within stage 1 DAM was tested with Mann-Whitney *U* test.

### Statistical analysis

All statistical analyses were performed using GraphPad Prism version 9.0 (RRID:SCR_002798). The significance of differences was assessed by the unpaired *t*-test, one- or two-way ANOVA, followed by the Tukey’s multiple comparison tests. A *p*-value of *p* < 0.05 was considered to represent a significance. All data are presented as mean ± SD or SEM.

## Supplementary Information

**Additional file 1. **GOBP terms affected by gossypetin in 5xFAD. List of GOBP terms affected by gossypetin treatment in each 5xFAD microglial subpopulation. 

**Additional file 2. **DEGs between vehicle treated 5xFAD and WT. List of DEGs between WT and 5xFAD homeostatic microglia, stage 1, and stage 2 DAM subpopulations in hippocampus and cortex. 

**Additional file 3. **DEGs between 5xFAD vehicle and gossypetin treated group. Lists of DEGs between vehicle and gossypetin treated 5xFAD homeostatic microglia, stage 1, and stage 2 DAM subpopulation in hippocampus and cortex.  

**Additional file 4. **Phagocytosis assay of BV2 treated with DMSO. Video showing slideshow of images taken from DMSO treated BV2 cell during phagocytic process under live cell imaging. Each image was taken with interval of 2 min. 

**Additional file 5. **Phagocytosis assay of BV2 treated with gossypetin. Video showing slideshow of images taken from gossypetin treated BV2 cell during phagocytic process under live cell imaging. Each image was taken with interval of 2 min. 

**Additional file 6: Fig.S1 **Gossypetin does not affect expression of β-, and γ-secretases and activity of β-secretase. (A to G) Time dependent β-secretase activity of mouse hippocampal lysate was measured with Relative Fluorescence Unit (RFU). Fluorescence excitation and emission wavelength was 335 nm and 495 nm respectively (A). Bar graph of RFU at each time point of 10 min (B), 20 min (C), 30 min (D), 40 min (E), 50 min (F), 60 min (G). (*n* = 10~12 mice per group) (H to L) Representative images of Western blot analysis for β-, γ-secretase subunits, and GAPDH (H). Bar graphs represent relative protein expression levels of BACE1 (I), Nicastrin (J), APH-1 (K), and PEN2 (L). (*n* = 12~15 mice per group) (M to P) Bar graphs represent relative mRNA expression level of β-, and γ-secretase subunits bace1 (M), ncstn (N), aph1 (O), pen2 (P). (*n* = 9~10 mice per group) Error bars represent the mean ± SD, *p < 0.05, ns = not significant, two-way ANOVA followed by Tukey’s multiple comparisons test. **Fig. S2** Cell type classification of brain samples. (A) UMAP plot showing all cells from the brain samples, colored by their cell types. (B) Heatmap illustrating the Z-scores of average normalized expressions of cell type markers. (C) Violin plots displaying the log-scaled number of detected genes (top), Unique Molecular Identifiers (UMIs) (middle), and the percentage of mitochondrial gene expressions (bottom) per cell for each cell type. (D) UMAP plots showing all cells from the brain samples, colored by their sampled region (left), mouse strain (middle), or drug administration (right) condition. **Fig. S3** Detailed subtyping of the microglial population. (A) UMAP plots showing all microglial cells from cortex region. The cells are colored by their celltypes (left). Heatmap showing the Z-scores of average normalized expressions of representative DEGs for each cell type from cortex region (right). (B) UMAP plots showing microglial cells from cortex (left) or hippocampus (right), colored by combination of mouse strain and drug administration condition. (C) UMAP plots illustrating microglial cells from cortex (left) or hippocampus (right), colored by their inferred cell cycle. (D) Bar plots for the fraction of cortex (left) or hippocampus (right) microglial cells by sample conditions, which are the combination of mouse strain and drug administration, for each microglial subtype. **Fig. S4** Differential gene expressions between vehicle- and gossypetin-treated microglia. (A) Scatter plot showing GOBP terms that are upregulated or downregulated by5xFAD construction or gossypetin administration for each microglial subtype from cortex. Significant (Fisher’s exact test, *P* < 0.01) terms associated with antigen presentation are colored by their biological keywords. (B) GSEA plots showing significant (*P*< 0.05) GOBP terms for gossypetin administration condition against vehicle treatment within 5xFAD homeostatic microglia from hippocampus region. Related to Fig. 3D. (C) Volcano plot illustrating the DEGs selected by the comparison between wild type and 5xFAD(left), or vehicle and gossypetin treated 5xFAD (right) from homeostatic microglial population of cortex region. **Fig. S5** Transcriptomic transition in cortex microglia and measurement of DAM signature score. (A) Volcano plot showing significant (*p* < 0.05) DEGs selected by the comparison between cortex homeostatic microglia in vehicle treated wild type and 5xFAD (top left), or vehicle and gossypetin treated 5xFAD (top right). Volcano plots illustrating comparison between gossypetin administration condition against vehicle treatment within 5xFAD stage 1 DAM (bottom left) or stage 2 DAM (bottom right) from cortex are also presented. (B) Violin plot illustrating module scores for the DAM-related genes from previous studies. Cells are grouped by the combination of their mouse strain and treatment condition. (***P < 0.001) **Fig. S6** Gossypetin ameliorates gliosis in microglia and astrocytes. (A to D) Representative images of hippocampus (A) and cortex (C) stained with Hoechst and Iba-1. Scale bar corresponds to 200μm. Bar graph represents quantification of Iba-1 positive area in dentate gyrus of hippocampus (*n* = 9~12 mice per group, 3~6 slices per brain) (B) and cortex (*n* = 9~12 mice per group, 3~6 slices per brain) (D). (E to H) Representative images of hippocampus (E) and cortex (G) stained with Hoechst and GFAP. Scale bar corresponds to 200μm. Bar graph represents quantification of GFAP positive area in dentate gyrus of hippocampus (*n* = 9~12 mice per group, 3~6 slices per brain) (F) and cortex (*n* = 9~12 mice per group, 3~5 slices per brain) (H). The error bars represent the mean ± SEM.****p <0.0001, ***p < 0.001, **p < 0.01, ns = not significant, two-way ANOVA followed by Tukey’s multiple comparisons test (B, D, F and H). **Fig. S7** Gossypetin increases Aβ phagocytic capacity and dynamics of BV2 microglial cell line. (A) Representative images of BV2 cells treated with 488-Aβ and stained with Hoechst and Iba-1. Gossypetin (25μM) was pretreated for 24 h before 488-Aβ treatment. Scale bar corresponds to 100μm. (B). Bar graph represents quantification of area of internalized 488-Aβ in BV2 (*n*= 3 per group, 253~656 cells per sample). (C) Line graph represents quantification of fluorescent area generated by internalized 488-Aβ in BV2 in a time dependent manner (*n* = 3 per group, 107~347 cells per sample). The error bars represent the mean ± SEM. ****p <0.0001, *p < 0.05, two-way ANOVA followed by Tukey’s multiple comparisons test (C), Student’s t test (B). 

## Data Availability

The scRNA-seq datasets generated and analyzed during the current study are available in the National Center for Biotechnology Information under accession number PRJNA766517.

## Abbreviations

Aβ: Beta amyloid
AD: Alzheimer’s disease
CNS: Central nervous system
DAM: Disease-associated microglia
MGnD: Neurodegenerative microglia
MWM: Morris Water Maze
AchE: Acetylcholinesterase
APP: Amyloid precursor protein
scRNA-seq: Single cell RNA sequencing
WT: Wild type
DEGs: Differentially expressed genes
GOBP: Gene ontology biological process
NES: Normalized enrichment score
GSEA: Gene set enrichment analysis
488 - Aβ: 488-tagged fluorescent Abeta
Cyto D: Cytochalasin D
LDAM: Lipid droplet accumulated microglia
