# Supplementary material for: Gossypetin ameliorates 5xFAD spatial learning and memory through enhanced phagocytosis against Aβ
Source: Alzheimers Res Ther. 2022 Oct 21;14:158. doi: 10.1186/s13195-022-01096-3 (PMC9585741; doi:10.1186/s13195-022-01096-3)

Figure S4. Differential gene expressions between vehicle and gossypetin treated 5xFAD microglia

A

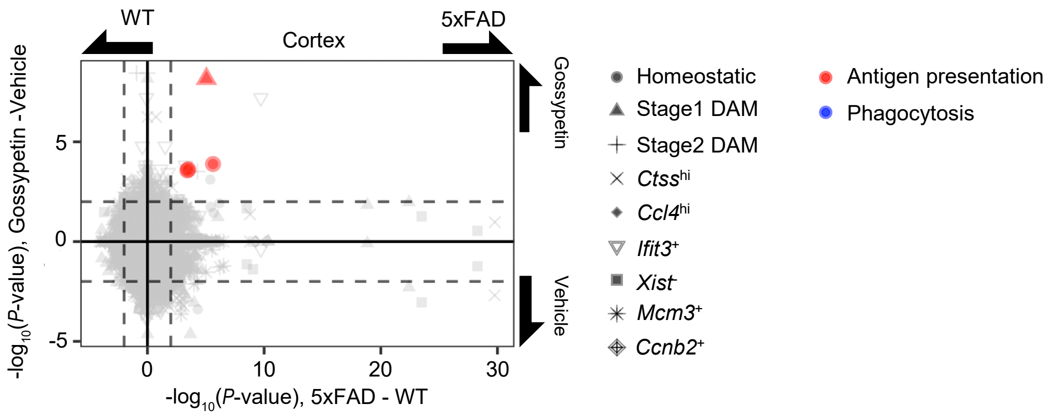

B

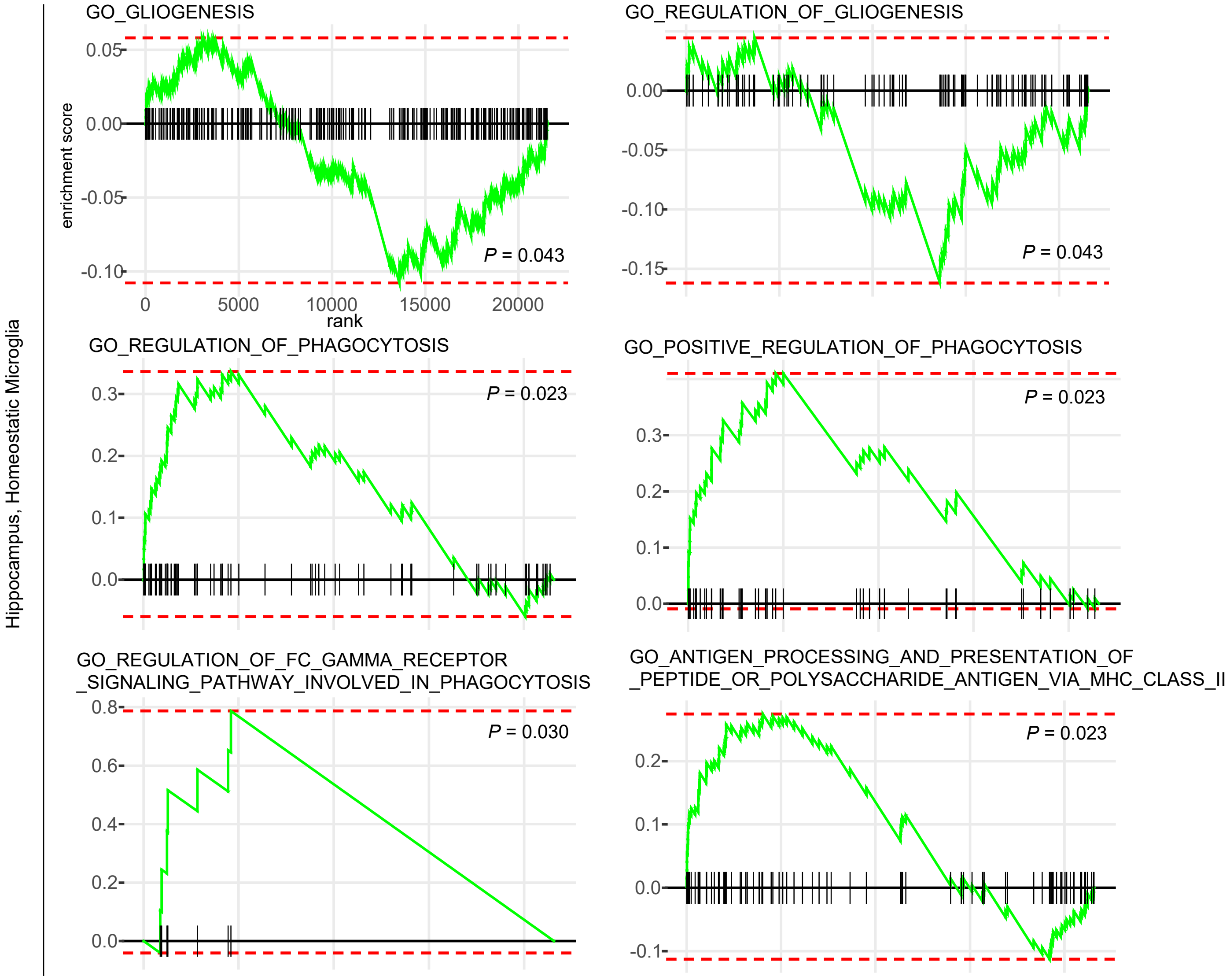

Supplement: Supplementary file 6 — Additional file 6: Fig.S1 Gossypetin does not affect expression of β-, and γ-secretases and activity of β-secretase. (A to G) Time dependent β-secretase activity of mouse hippocampal lysate was measured with Relative Fluorescence Unit (RFU). Fluorescence excitation and emission wavelength was 335 nm and 495 nm respectively (A). Bar graph of RFU at each time point of 10 min (B), 20 min (C), 30 min (D), 40 min (E), 50 min (F), 60 min (G). (n = 10~12 mice per group) (H to L) Representative images of Western blot analysis for β-, γ-secretase subunits, and GAPDH (H). Bar graphs represent relative protein expression levels of BACE1 (I), Nicastrin (J), APH-1 (K), and PEN2 (L). (n = 12~15 mice per group) (M to P) Bar graphs represent relative mRNA expression level of β-, and γ-secretase subunits bace1 (M), ncstn (N), aph1 (O), pen2 (P). (n = 9~10 mice per group) Error bars represent the mean ± SD, *p < 0.05, ns = not significant, two-way ANOVA followed by Tukey’s multiple comparisons test. Fig. S2 Cell type classification of brain samples. (A) UMAP plot showing all cells from the brain samples, colored by their cell types. (B) Heatmap illustrating the Z-scores of average normalized expressions of cell type markers. (C) Violin plots displaying the log-scaled number of detected genes (top), Unique Molecular Identifiers (UMIs) (middle), and the percentage of mitochondrial gene expressions (bottom) per cell for each cell type. (D) UMAP plots showing all cells from the brain samples, colored by their sampled region (left), mouse strain (middle), or drug administration (right) condition. Fig. S3 Detailed subtyping of the microglial population. (A) UMAP plots showing all microglial cells from cortex region. The cells are colored by their celltypes (left). Heatmap showing the Z-scores of average normalized expressions of representative DEGs for each cell type from cortex region (right). (B) UMAP plots showing microglial cells from cortex (left) or hippocampus (right), colored by co [file 13195_2022_1096_MOESM6_ESM.zip › Figure S4 (Total).pdf]
