# Supplementary figures and images for: Gossypetin ameliorates 5xFAD spatial learning and memory through enhanced phagocytosis against Aβ
Source: Alzheimers Res Ther. 2022 Oct 21;14:158. doi: 10.1186/s13195-022-01096-3 (PMC9585741; doi:10.1186/s13195-022-01096-3)

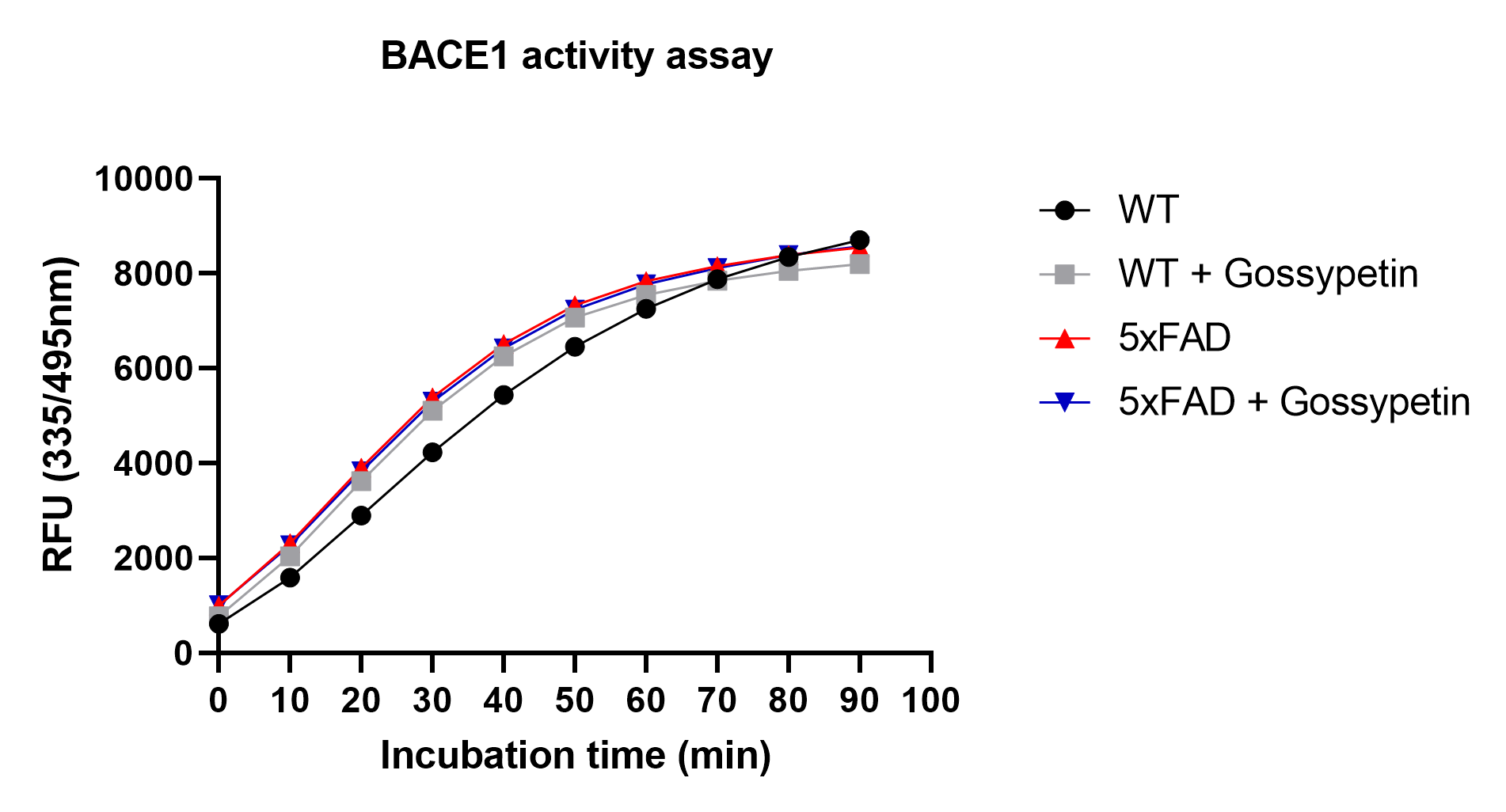

Supplement: Supplementary file 6 — Additional file 6: Fig.S1 Gossypetin does not affect expression of β-, and γ-secretases and activity of β-secretase. (A to G) Time dependent β-secretase activity of mouse hippocampal lysate was measured with Relative Fluorescence Unit (RFU). Fluorescence excitation and emission wavelength was 335 nm and 495 nm respectively (A). Bar graph of RFU at each time point of 10 min (B), 20 min (C), 30 min (D), 40 min (E), 50 min (F), 60 min (G). (n = 10~12 mice per group) (H to L) Representative images of Western blot analysis for β-, γ-secretase subunits, and GAPDH (H). Bar graphs represent relative protein expression levels of BACE1 (I), Nicastrin (J), APH-1 (K), and PEN2 (L). (n = 12~15 mice per group) (M to P) Bar graphs represent relative mRNA expression level of β-, and γ-secretase subunits bace1 (M), ncstn (N), aph1 (O), pen2 (P). (n = 9~10 mice per group) Error bars represent the mean ± SD, *p < 0.05, ns = not significant, two-way ANOVA followed by Tukey’s multiple comparisons test. Fig. S2 Cell type classification of brain samples. (A) UMAP plot showing all cells from the brain samples, colored by their cell types. (B) Heatmap illustrating the Z-scores of average normalized expressions of cell type markers. (C) Violin plots displaying the log-scaled number of detected genes (top), Unique Molecular Identifiers (UMIs) (middle), and the percentage of mitochondrial gene expressions (bottom) per cell for each cell type. (D) UMAP plots showing all cells from the brain samples, colored by their sampled region (left), mouse strain (middle), or drug administration (right) condition. Fig. S3 Detailed subtyping of the microglial population. (A) UMAP plots showing all microglial cells from cortex region. The cells are colored by their celltypes (left). Heatmap showing the Z-scores of average normalized expressions of representative DEGs for each cell type from cortex region (right). (B) UMAP plots showing microglial cells from cortex (left) or hippocampus (right), colored by co [file 13195_2022_1096_MOESM6_ESM.zip › Figure S1A.png]

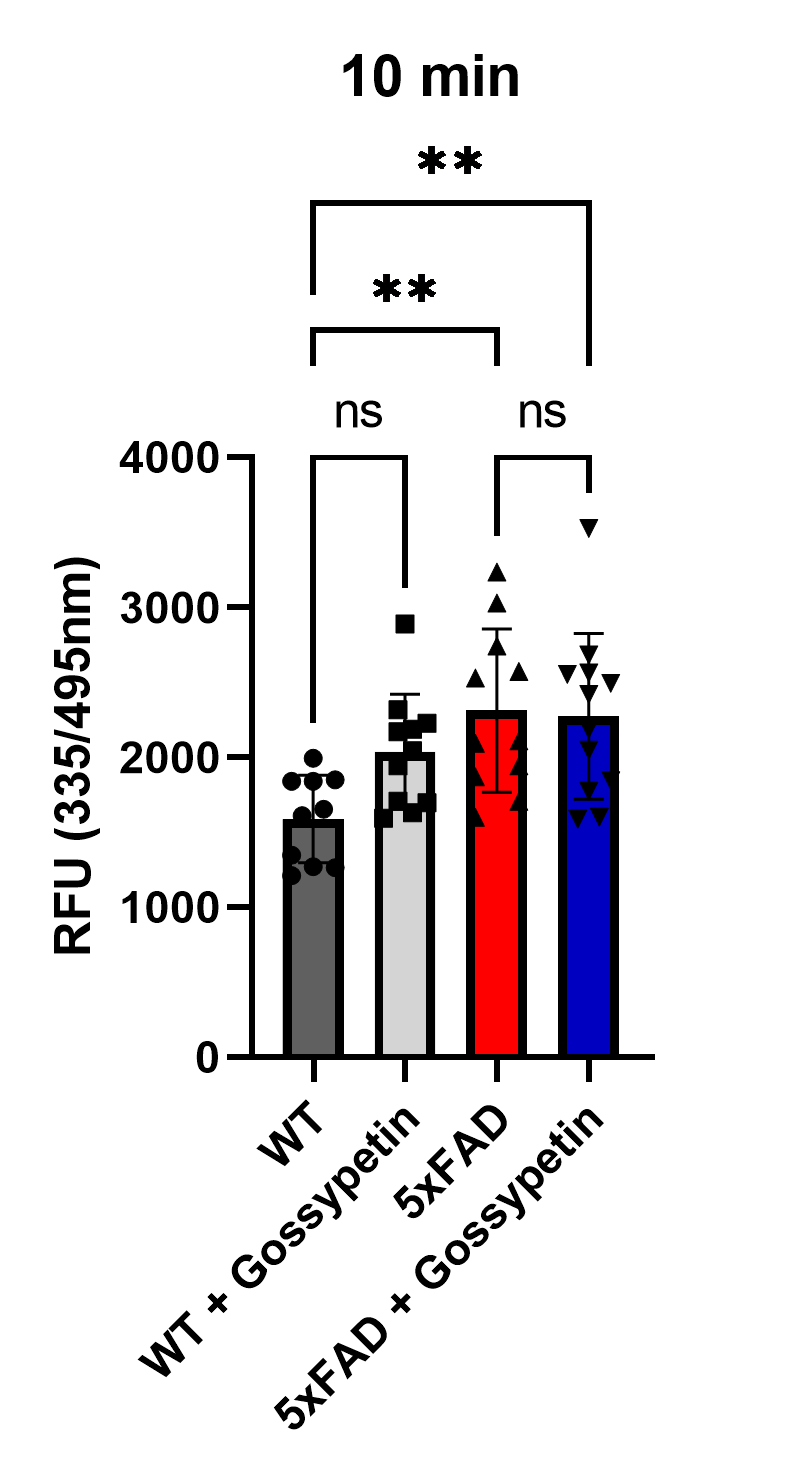

Supplement: Supplementary file 6 — Additional file 6: Fig.S1 Gossypetin does not affect expression of β-, and γ-secretases and activity of β-secretase. (A to G) Time dependent β-secretase activity of mouse hippocampal lysate was measured with Relative Fluorescence Unit (RFU). Fluorescence excitation and emission wavelength was 335 nm and 495 nm respectively (A). Bar graph of RFU at each time point of 10 min (B), 20 min (C), 30 min (D), 40 min (E), 50 min (F), 60 min (G). (n = 10~12 mice per group) (H to L) Representative images of Western blot analysis for β-, γ-secretase subunits, and GAPDH (H). Bar graphs represent relative protein expression levels of BACE1 (I), Nicastrin (J), APH-1 (K), and PEN2 (L). (n = 12~15 mice per group) (M to P) Bar graphs represent relative mRNA expression level of β-, and γ-secretase subunits bace1 (M), ncstn (N), aph1 (O), pen2 (P). (n = 9~10 mice per group) Error bars represent the mean ± SD, *p < 0.05, ns = not significant, two-way ANOVA followed by Tukey’s multiple comparisons test. Fig. S2 Cell type classification of brain samples. (A) UMAP plot showing all cells from the brain samples, colored by their cell types. (B) Heatmap illustrating the Z-scores of average normalized expressions of cell type markers. (C) Violin plots displaying the log-scaled number of detected genes (top), Unique Molecular Identifiers (UMIs) (middle), and the percentage of mitochondrial gene expressions (bottom) per cell for each cell type. (D) UMAP plots showing all cells from the brain samples, colored by their sampled region (left), mouse strain (middle), or drug administration (right) condition. Fig. S3 Detailed subtyping of the microglial population. (A) UMAP plots showing all microglial cells from cortex region. The cells are colored by their celltypes (left). Heatmap showing the Z-scores of average normalized expressions of representative DEGs for each cell type from cortex region (right). (B) UMAP plots showing microglial cells from cortex (left) or hippocampus (right), colored by co [file 13195_2022_1096_MOESM6_ESM.zip › Figure S1B.png]

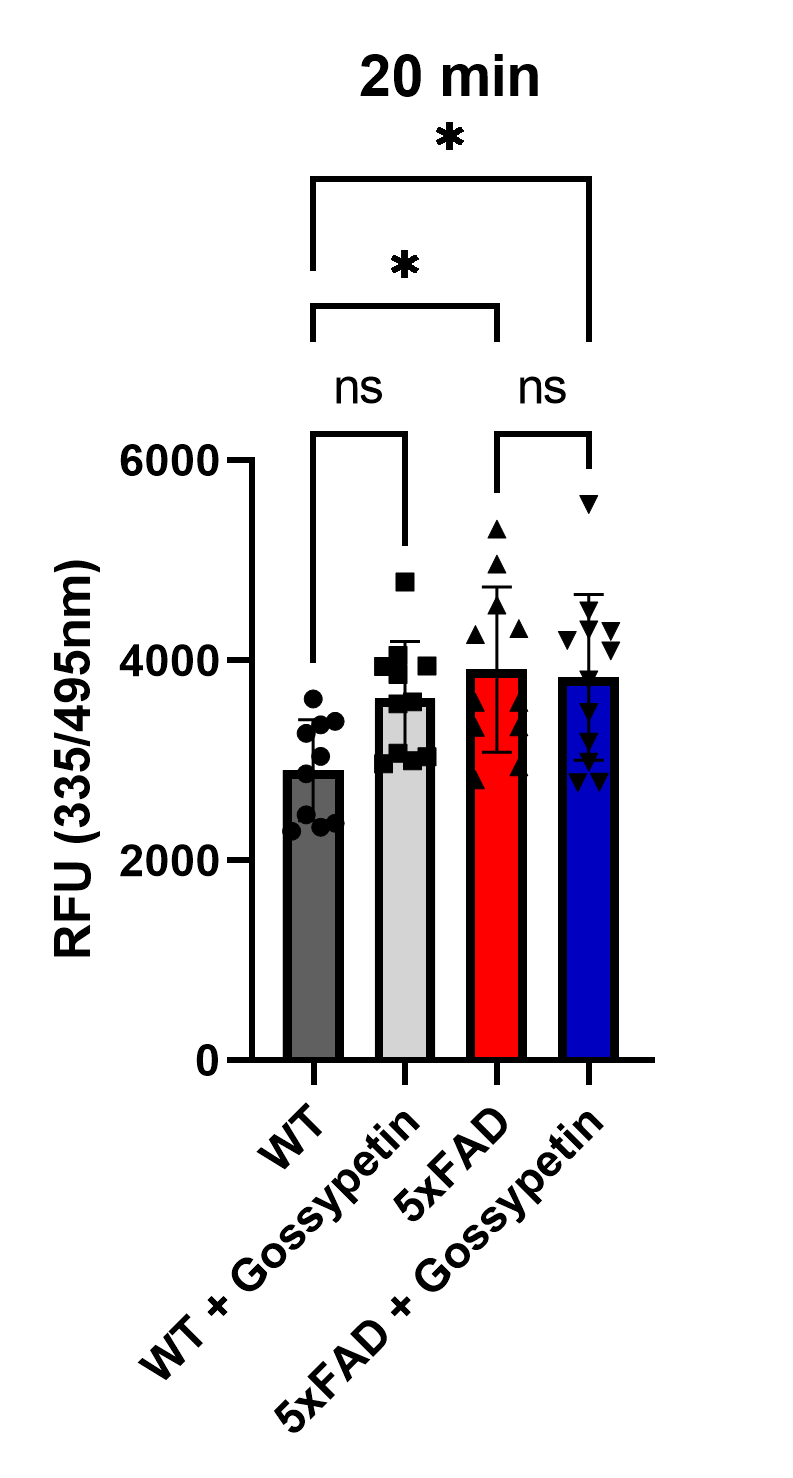

Supplement: Supplementary file 6 — Additional file 6: Fig.S1 Gossypetin does not affect expression of β-, and γ-secretases and activity of β-secretase. (A to G) Time dependent β-secretase activity of mouse hippocampal lysate was measured with Relative Fluorescence Unit (RFU). Fluorescence excitation and emission wavelength was 335 nm and 495 nm respectively (A). Bar graph of RFU at each time point of 10 min (B), 20 min (C), 30 min (D), 40 min (E), 50 min (F), 60 min (G). (n = 10~12 mice per group) (H to L) Representative images of Western blot analysis for β-, γ-secretase subunits, and GAPDH (H). Bar graphs represent relative protein expression levels of BACE1 (I), Nicastrin (J), APH-1 (K), and PEN2 (L). (n = 12~15 mice per group) (M to P) Bar graphs represent relative mRNA expression level of β-, and γ-secretase subunits bace1 (M), ncstn (N), aph1 (O), pen2 (P). (n = 9~10 mice per group) Error bars represent the mean ± SD, *p < 0.05, ns = not significant, two-way ANOVA followed by Tukey’s multiple comparisons test. Fig. S2 Cell type classification of brain samples. (A) UMAP plot showing all cells from the brain samples, colored by their cell types. (B) Heatmap illustrating the Z-scores of average normalized expressions of cell type markers. (C) Violin plots displaying the log-scaled number of detected genes (top), Unique Molecular Identifiers (UMIs) (middle), and the percentage of mitochondrial gene expressions (bottom) per cell for each cell type. (D) UMAP plots showing all cells from the brain samples, colored by their sampled region (left), mouse strain (middle), or drug administration (right) condition. Fig. S3 Detailed subtyping of the microglial population. (A) UMAP plots showing all microglial cells from cortex region. The cells are colored by their celltypes (left). Heatmap showing the Z-scores of average normalized expressions of representative DEGs for each cell type from cortex region (right). (B) UMAP plots showing microglial cells from cortex (left) or hippocampus (right), colored by co [file 13195_2022_1096_MOESM6_ESM.zip › Figure S1C.png]

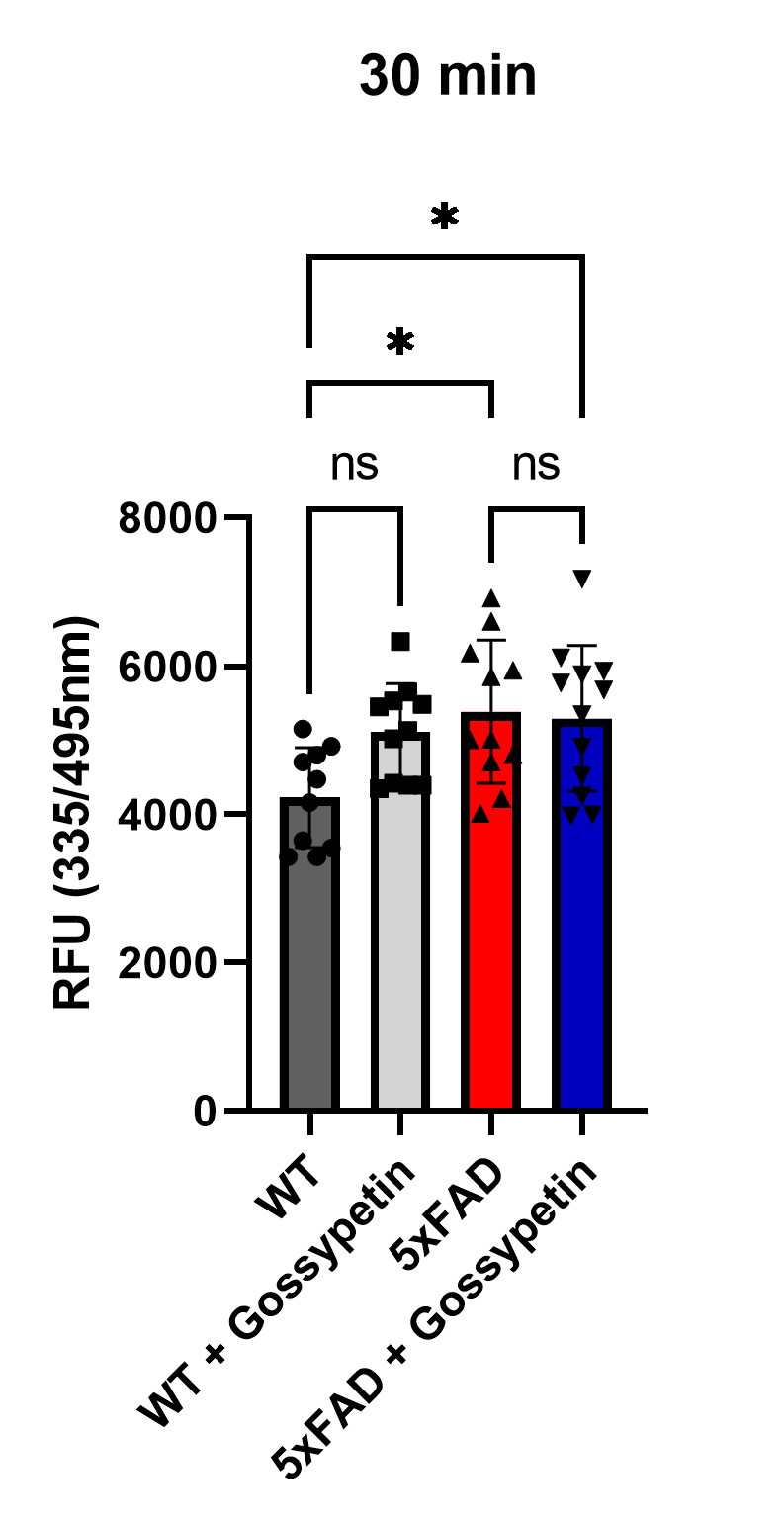

Supplement: Supplementary file 6 — Additional file 6: Fig.S1 Gossypetin does not affect expression of β-, and γ-secretases and activity of β-secretase. (A to G) Time dependent β-secretase activity of mouse hippocampal lysate was measured with Relative Fluorescence Unit (RFU). Fluorescence excitation and emission wavelength was 335 nm and 495 nm respectively (A). Bar graph of RFU at each time point of 10 min (B), 20 min (C), 30 min (D), 40 min (E), 50 min (F), 60 min (G). (n = 10~12 mice per group) (H to L) Representative images of Western blot analysis for β-, γ-secretase subunits, and GAPDH (H). Bar graphs represent relative protein expression levels of BACE1 (I), Nicastrin (J), APH-1 (K), and PEN2 (L). (n = 12~15 mice per group) (M to P) Bar graphs represent relative mRNA expression level of β-, and γ-secretase subunits bace1 (M), ncstn (N), aph1 (O), pen2 (P). (n = 9~10 mice per group) Error bars represent the mean ± SD, *p < 0.05, ns = not significant, two-way ANOVA followed by Tukey’s multiple comparisons test. Fig. S2 Cell type classification of brain samples. (A) UMAP plot showing all cells from the brain samples, colored by their cell types. (B) Heatmap illustrating the Z-scores of average normalized expressions of cell type markers. (C) Violin plots displaying the log-scaled number of detected genes (top), Unique Molecular Identifiers (UMIs) (middle), and the percentage of mitochondrial gene expressions (bottom) per cell for each cell type. (D) UMAP plots showing all cells from the brain samples, colored by their sampled region (left), mouse strain (middle), or drug administration (right) condition. Fig. S3 Detailed subtyping of the microglial population. (A) UMAP plots showing all microglial cells from cortex region. The cells are colored by their celltypes (left). Heatmap showing the Z-scores of average normalized expressions of representative DEGs for each cell type from cortex region (right). (B) UMAP plots showing microglial cells from cortex (left) or hippocampus (right), colored by co [file 13195_2022_1096_MOESM6_ESM.zip › Figure S1D.png]

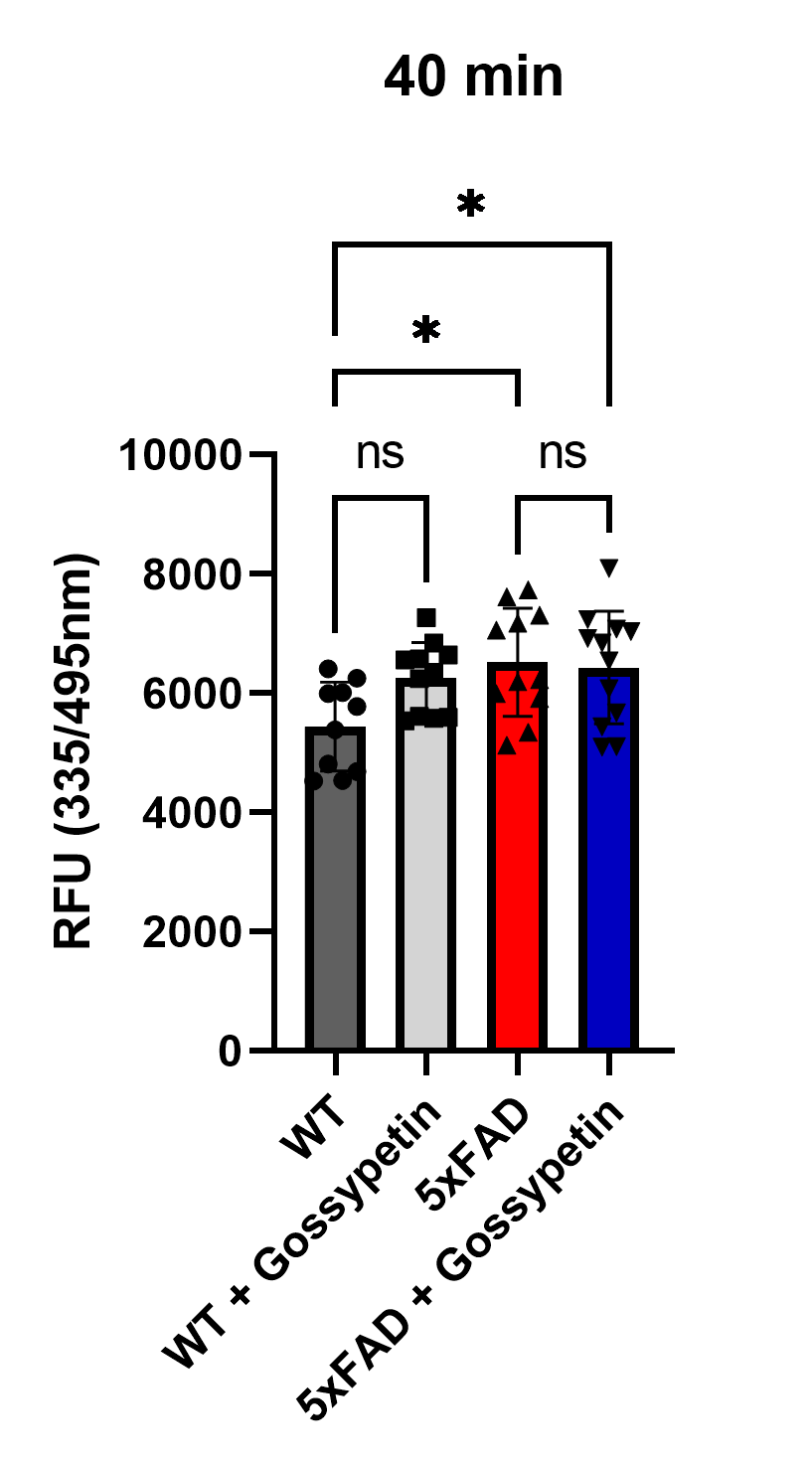

Supplement: Supplementary file 6 — Additional file 6: Fig.S1 Gossypetin does not affect expression of β-, and γ-secretases and activity of β-secretase. (A to G) Time dependent β-secretase activity of mouse hippocampal lysate was measured with Relative Fluorescence Unit (RFU). Fluorescence excitation and emission wavelength was 335 nm and 495 nm respectively (A). Bar graph of RFU at each time point of 10 min (B), 20 min (C), 30 min (D), 40 min (E), 50 min (F), 60 min (G). (n = 10~12 mice per group) (H to L) Representative images of Western blot analysis for β-, γ-secretase subunits, and GAPDH (H). Bar graphs represent relative protein expression levels of BACE1 (I), Nicastrin (J), APH-1 (K), and PEN2 (L). (n = 12~15 mice per group) (M to P) Bar graphs represent relative mRNA expression level of β-, and γ-secretase subunits bace1 (M), ncstn (N), aph1 (O), pen2 (P). (n = 9~10 mice per group) Error bars represent the mean ± SD, *p < 0.05, ns = not significant, two-way ANOVA followed by Tukey’s multiple comparisons test. Fig. S2 Cell type classification of brain samples. (A) UMAP plot showing all cells from the brain samples, colored by their cell types. (B) Heatmap illustrating the Z-scores of average normalized expressions of cell type markers. (C) Violin plots displaying the log-scaled number of detected genes (top), Unique Molecular Identifiers (UMIs) (middle), and the percentage of mitochondrial gene expressions (bottom) per cell for each cell type. (D) UMAP plots showing all cells from the brain samples, colored by their sampled region (left), mouse strain (middle), or drug administration (right) condition. Fig. S3 Detailed subtyping of the microglial population. (A) UMAP plots showing all microglial cells from cortex region. The cells are colored by their celltypes (left). Heatmap showing the Z-scores of average normalized expressions of representative DEGs for each cell type from cortex region (right). (B) UMAP plots showing microglial cells from cortex (left) or hippocampus (right), colored by co [file 13195_2022_1096_MOESM6_ESM.zip › Figure S1E.png]

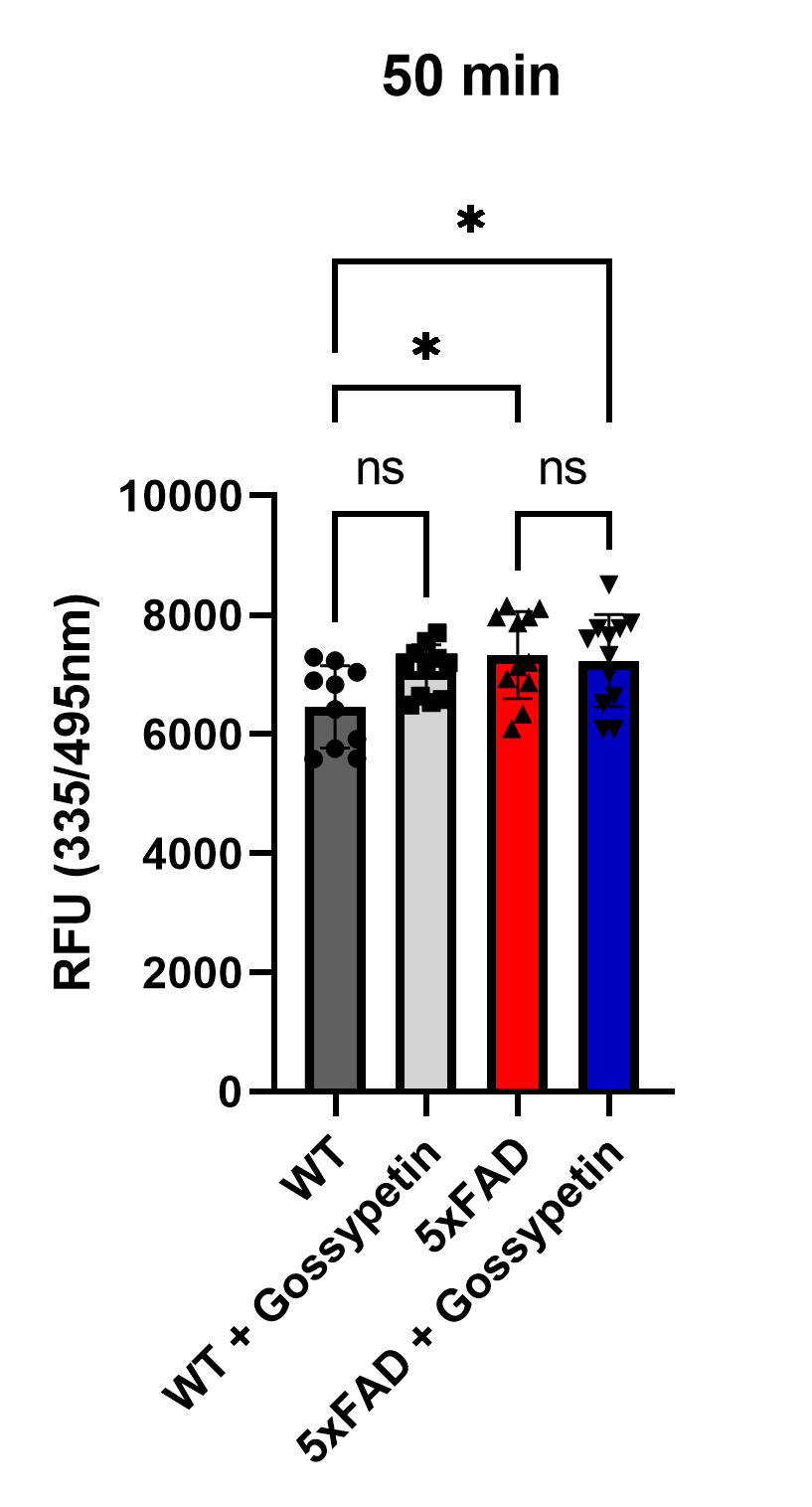

Supplement: Supplementary file 6 — Additional file 6: Fig.S1 Gossypetin does not affect expression of β-, and γ-secretases and activity of β-secretase. (A to G) Time dependent β-secretase activity of mouse hippocampal lysate was measured with Relative Fluorescence Unit (RFU). Fluorescence excitation and emission wavelength was 335 nm and 495 nm respectively (A). Bar graph of RFU at each time point of 10 min (B), 20 min (C), 30 min (D), 40 min (E), 50 min (F), 60 min (G). (n = 10~12 mice per group) (H to L) Representative images of Western blot analysis for β-, γ-secretase subunits, and GAPDH (H). Bar graphs represent relative protein expression levels of BACE1 (I), Nicastrin (J), APH-1 (K), and PEN2 (L). (n = 12~15 mice per group) (M to P) Bar graphs represent relative mRNA expression level of β-, and γ-secretase subunits bace1 (M), ncstn (N), aph1 (O), pen2 (P). (n = 9~10 mice per group) Error bars represent the mean ± SD, *p < 0.05, ns = not significant, two-way ANOVA followed by Tukey’s multiple comparisons test. Fig. S2 Cell type classification of brain samples. (A) UMAP plot showing all cells from the brain samples, colored by their cell types. (B) Heatmap illustrating the Z-scores of average normalized expressions of cell type markers. (C) Violin plots displaying the log-scaled number of detected genes (top), Unique Molecular Identifiers (UMIs) (middle), and the percentage of mitochondrial gene expressions (bottom) per cell for each cell type. (D) UMAP plots showing all cells from the brain samples, colored by their sampled region (left), mouse strain (middle), or drug administration (right) condition. Fig. S3 Detailed subtyping of the microglial population. (A) UMAP plots showing all microglial cells from cortex region. The cells are colored by their celltypes (left). Heatmap showing the Z-scores of average normalized expressions of representative DEGs for each cell type from cortex region (right). (B) UMAP plots showing microglial cells from cortex (left) or hippocampus (right), colored by co [file 13195_2022_1096_MOESM6_ESM.zip › Figure S1F.png]

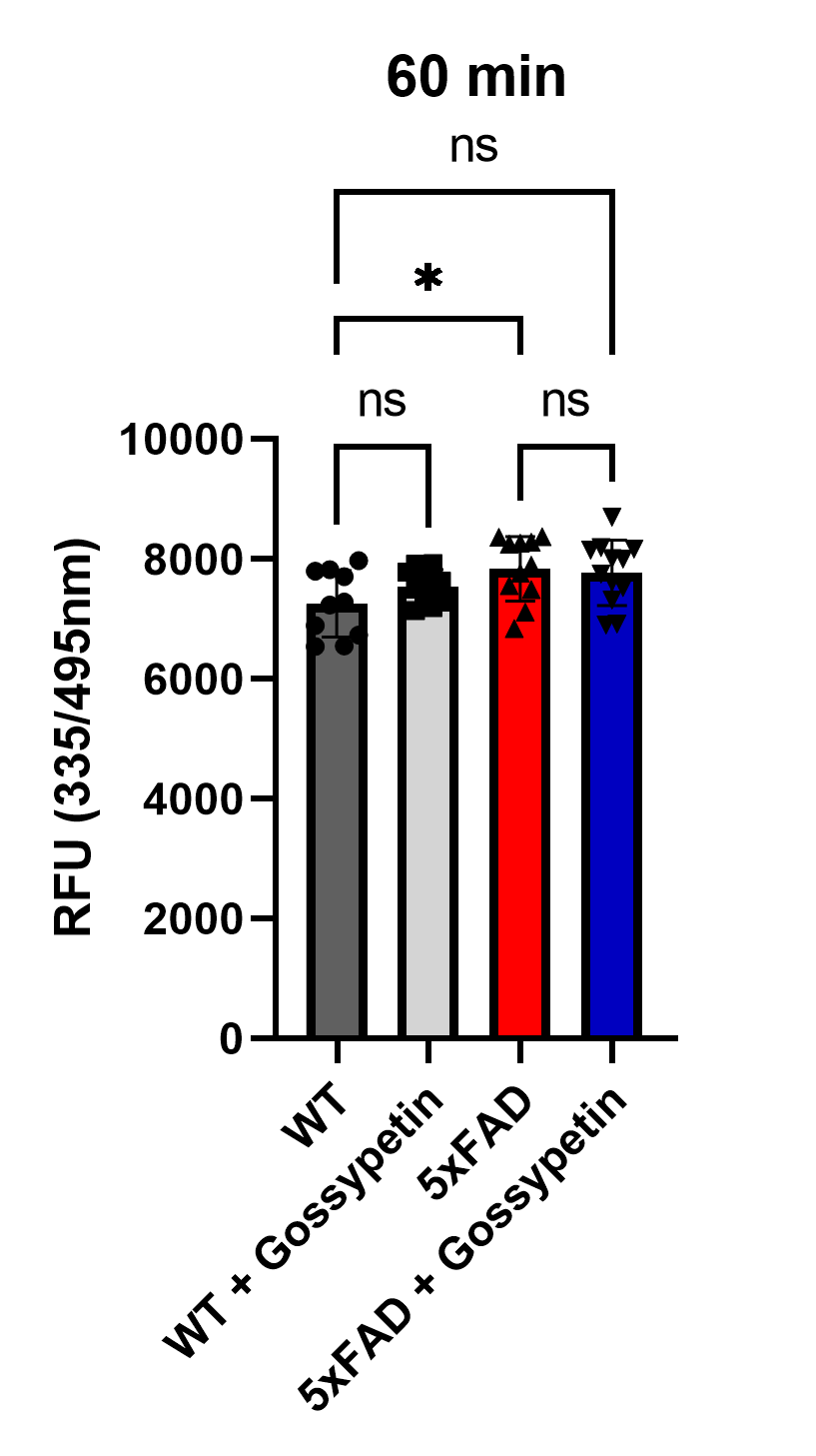

Supplement: Supplementary file 6 — Additional file 6: Fig.S1 Gossypetin does not affect expression of β-, and γ-secretases and activity of β-secretase. (A to G) Time dependent β-secretase activity of mouse hippocampal lysate was measured with Relative Fluorescence Unit (RFU). Fluorescence excitation and emission wavelength was 335 nm and 495 nm respectively (A). Bar graph of RFU at each time point of 10 min (B), 20 min (C), 30 min (D), 40 min (E), 50 min (F), 60 min (G). (n = 10~12 mice per group) (H to L) Representative images of Western blot analysis for β-, γ-secretase subunits, and GAPDH (H). Bar graphs represent relative protein expression levels of BACE1 (I), Nicastrin (J), APH-1 (K), and PEN2 (L). (n = 12~15 mice per group) (M to P) Bar graphs represent relative mRNA expression level of β-, and γ-secretase subunits bace1 (M), ncstn (N), aph1 (O), pen2 (P). (n = 9~10 mice per group) Error bars represent the mean ± SD, *p < 0.05, ns = not significant, two-way ANOVA followed by Tukey’s multiple comparisons test. Fig. S2 Cell type classification of brain samples. (A) UMAP plot showing all cells from the brain samples, colored by their cell types. (B) Heatmap illustrating the Z-scores of average normalized expressions of cell type markers. (C) Violin plots displaying the log-scaled number of detected genes (top), Unique Molecular Identifiers (UMIs) (middle), and the percentage of mitochondrial gene expressions (bottom) per cell for each cell type. (D) UMAP plots showing all cells from the brain samples, colored by their sampled region (left), mouse strain (middle), or drug administration (right) condition. Fig. S3 Detailed subtyping of the microglial population. (A) UMAP plots showing all microglial cells from cortex region. The cells are colored by their celltypes (left). Heatmap showing the Z-scores of average normalized expressions of representative DEGs for each cell type from cortex region (right). (B) UMAP plots showing microglial cells from cortex (left) or hippocampus (right), colored by co [file 13195_2022_1096_MOESM6_ESM.zip › Figure S1G.png]

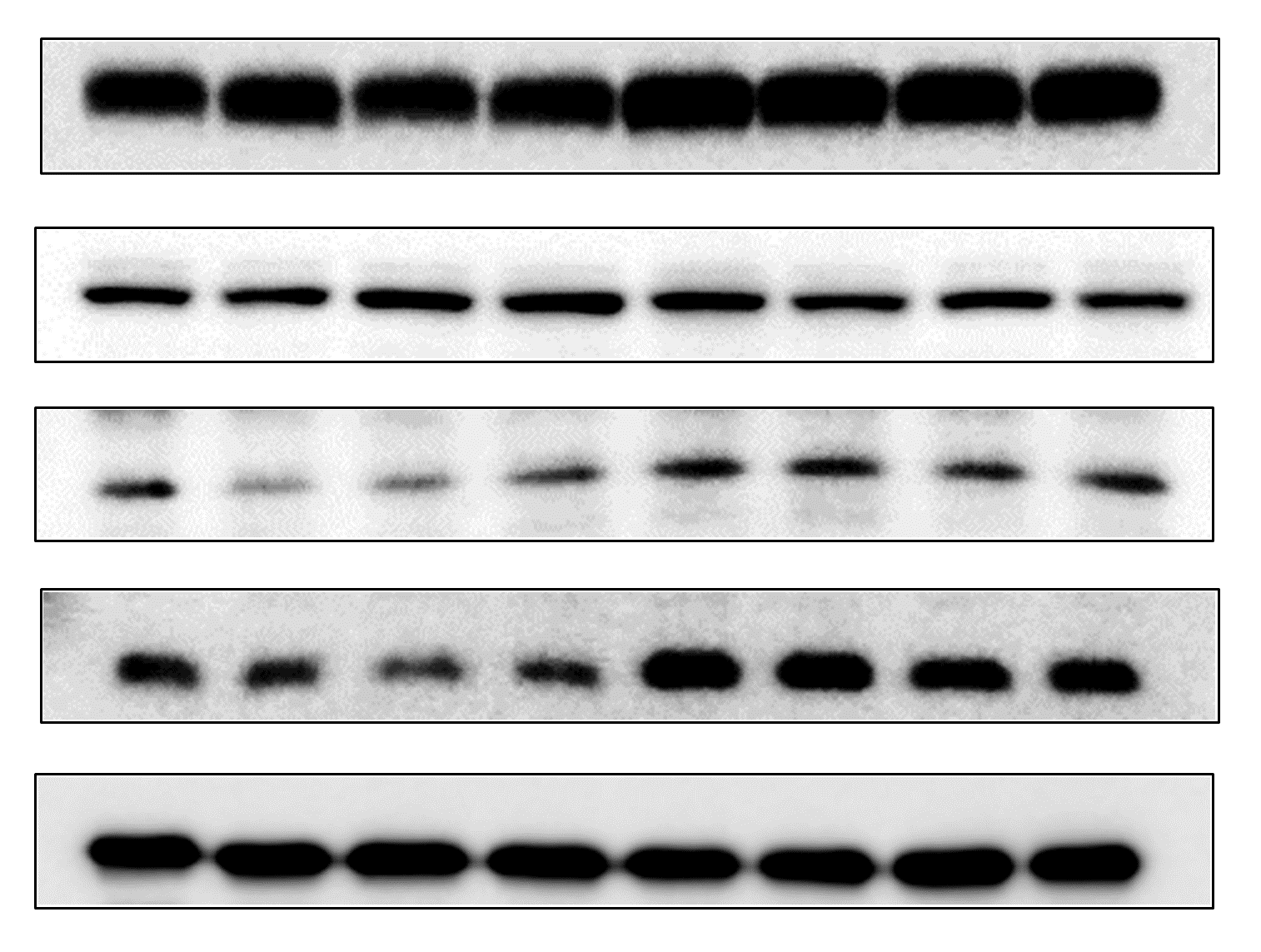

Supplement: Supplementary file 6 — Additional file 6: Fig.S1 Gossypetin does not affect expression of β-, and γ-secretases and activity of β-secretase. (A to G) Time dependent β-secretase activity of mouse hippocampal lysate was measured with Relative Fluorescence Unit (RFU). Fluorescence excitation and emission wavelength was 335 nm and 495 nm respectively (A). Bar graph of RFU at each time point of 10 min (B), 20 min (C), 30 min (D), 40 min (E), 50 min (F), 60 min (G). (n = 10~12 mice per group) (H to L) Representative images of Western blot analysis for β-, γ-secretase subunits, and GAPDH (H). Bar graphs represent relative protein expression levels of BACE1 (I), Nicastrin (J), APH-1 (K), and PEN2 (L). (n = 12~15 mice per group) (M to P) Bar graphs represent relative mRNA expression level of β-, and γ-secretase subunits bace1 (M), ncstn (N), aph1 (O), pen2 (P). (n = 9~10 mice per group) Error bars represent the mean ± SD, *p < 0.05, ns = not significant, two-way ANOVA followed by Tukey’s multiple comparisons test. Fig. S2 Cell type classification of brain samples. (A) UMAP plot showing all cells from the brain samples, colored by their cell types. (B) Heatmap illustrating the Z-scores of average normalized expressions of cell type markers. (C) Violin plots displaying the log-scaled number of detected genes (top), Unique Molecular Identifiers (UMIs) (middle), and the percentage of mitochondrial gene expressions (bottom) per cell for each cell type. (D) UMAP plots showing all cells from the brain samples, colored by their sampled region (left), mouse strain (middle), or drug administration (right) condition. Fig. S3 Detailed subtyping of the microglial population. (A) UMAP plots showing all microglial cells from cortex region. The cells are colored by their celltypes (left). Heatmap showing the Z-scores of average normalized expressions of representative DEGs for each cell type from cortex region (right). (B) UMAP plots showing microglial cells from cortex (left) or hippocampus (right), colored by co [file 13195_2022_1096_MOESM6_ESM.zip › Figure S1H.png]

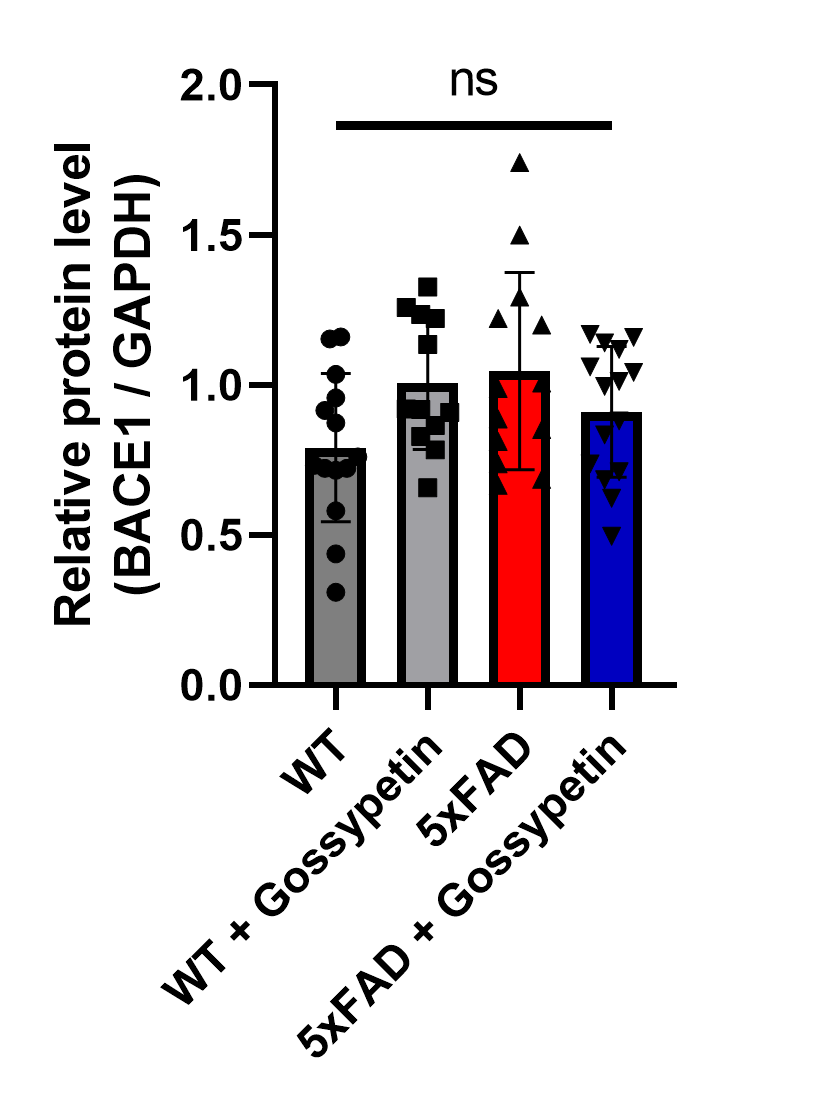

Supplement: Supplementary file 6 — Additional file 6: Fig.S1 Gossypetin does not affect expression of β-, and γ-secretases and activity of β-secretase. (A to G) Time dependent β-secretase activity of mouse hippocampal lysate was measured with Relative Fluorescence Unit (RFU). Fluorescence excitation and emission wavelength was 335 nm and 495 nm respectively (A). Bar graph of RFU at each time point of 10 min (B), 20 min (C), 30 min (D), 40 min (E), 50 min (F), 60 min (G). (n = 10~12 mice per group) (H to L) Representative images of Western blot analysis for β-, γ-secretase subunits, and GAPDH (H). Bar graphs represent relative protein expression levels of BACE1 (I), Nicastrin (J), APH-1 (K), and PEN2 (L). (n = 12~15 mice per group) (M to P) Bar graphs represent relative mRNA expression level of β-, and γ-secretase subunits bace1 (M), ncstn (N), aph1 (O), pen2 (P). (n = 9~10 mice per group) Error bars represent the mean ± SD, *p < 0.05, ns = not significant, two-way ANOVA followed by Tukey’s multiple comparisons test. Fig. S2 Cell type classification of brain samples. (A) UMAP plot showing all cells from the brain samples, colored by their cell types. (B) Heatmap illustrating the Z-scores of average normalized expressions of cell type markers. (C) Violin plots displaying the log-scaled number of detected genes (top), Unique Molecular Identifiers (UMIs) (middle), and the percentage of mitochondrial gene expressions (bottom) per cell for each cell type. (D) UMAP plots showing all cells from the brain samples, colored by their sampled region (left), mouse strain (middle), or drug administration (right) condition. Fig. S3 Detailed subtyping of the microglial population. (A) UMAP plots showing all microglial cells from cortex region. The cells are colored by their celltypes (left). Heatmap showing the Z-scores of average normalized expressions of representative DEGs for each cell type from cortex region (right). (B) UMAP plots showing microglial cells from cortex (left) or hippocampus (right), colored by co [file 13195_2022_1096_MOESM6_ESM.zip › Figure S1I.png]

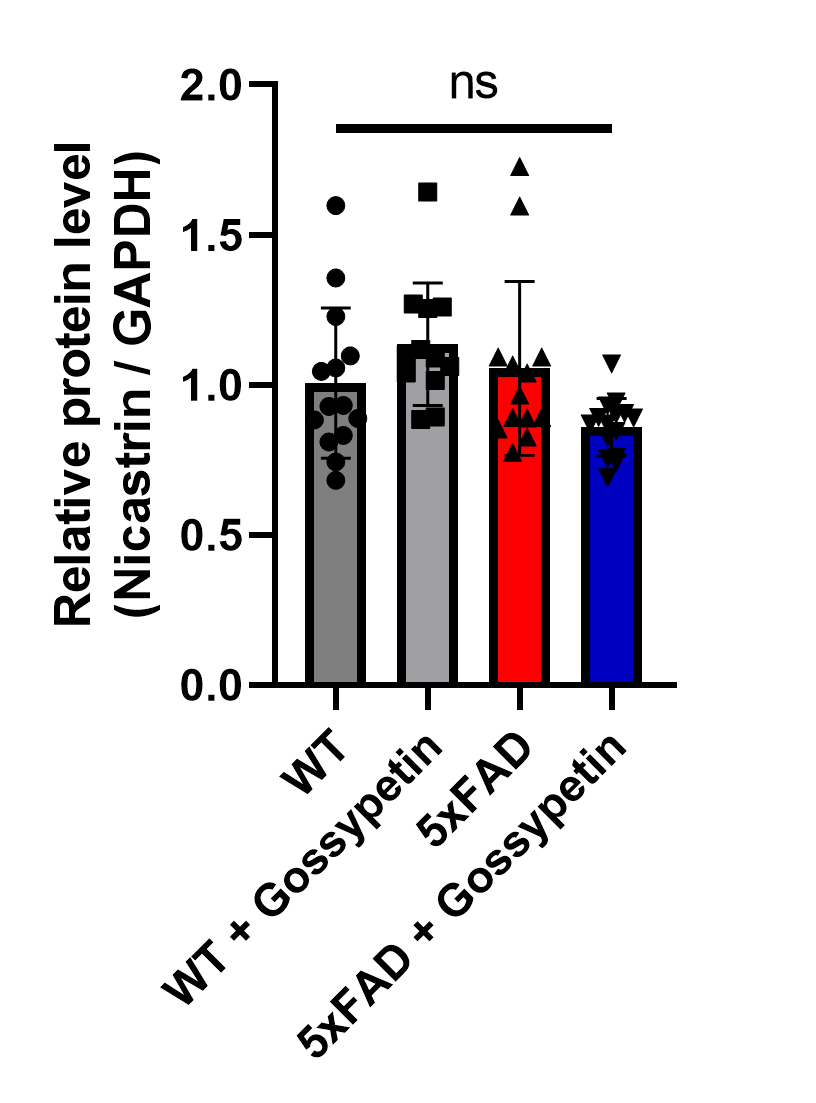

Supplement: Supplementary file 6 — Additional file 6: Fig.S1 Gossypetin does not affect expression of β-, and γ-secretases and activity of β-secretase. (A to G) Time dependent β-secretase activity of mouse hippocampal lysate was measured with Relative Fluorescence Unit (RFU). Fluorescence excitation and emission wavelength was 335 nm and 495 nm respectively (A). Bar graph of RFU at each time point of 10 min (B), 20 min (C), 30 min (D), 40 min (E), 50 min (F), 60 min (G). (n = 10~12 mice per group) (H to L) Representative images of Western blot analysis for β-, γ-secretase subunits, and GAPDH (H). Bar graphs represent relative protein expression levels of BACE1 (I), Nicastrin (J), APH-1 (K), and PEN2 (L). (n = 12~15 mice per group) (M to P) Bar graphs represent relative mRNA expression level of β-, and γ-secretase subunits bace1 (M), ncstn (N), aph1 (O), pen2 (P). (n = 9~10 mice per group) Error bars represent the mean ± SD, *p < 0.05, ns = not significant, two-way ANOVA followed by Tukey’s multiple comparisons test. Fig. S2 Cell type classification of brain samples. (A) UMAP plot showing all cells from the brain samples, colored by their cell types. (B) Heatmap illustrating the Z-scores of average normalized expressions of cell type markers. (C) Violin plots displaying the log-scaled number of detected genes (top), Unique Molecular Identifiers (UMIs) (middle), and the percentage of mitochondrial gene expressions (bottom) per cell for each cell type. (D) UMAP plots showing all cells from the brain samples, colored by their sampled region (left), mouse strain (middle), or drug administration (right) condition. Fig. S3 Detailed subtyping of the microglial population. (A) UMAP plots showing all microglial cells from cortex region. The cells are colored by their celltypes (left). Heatmap showing the Z-scores of average normalized expressions of representative DEGs for each cell type from cortex region (right). (B) UMAP plots showing microglial cells from cortex (left) or hippocampus (right), colored by co [file 13195_2022_1096_MOESM6_ESM.zip › Figure S1J.png]

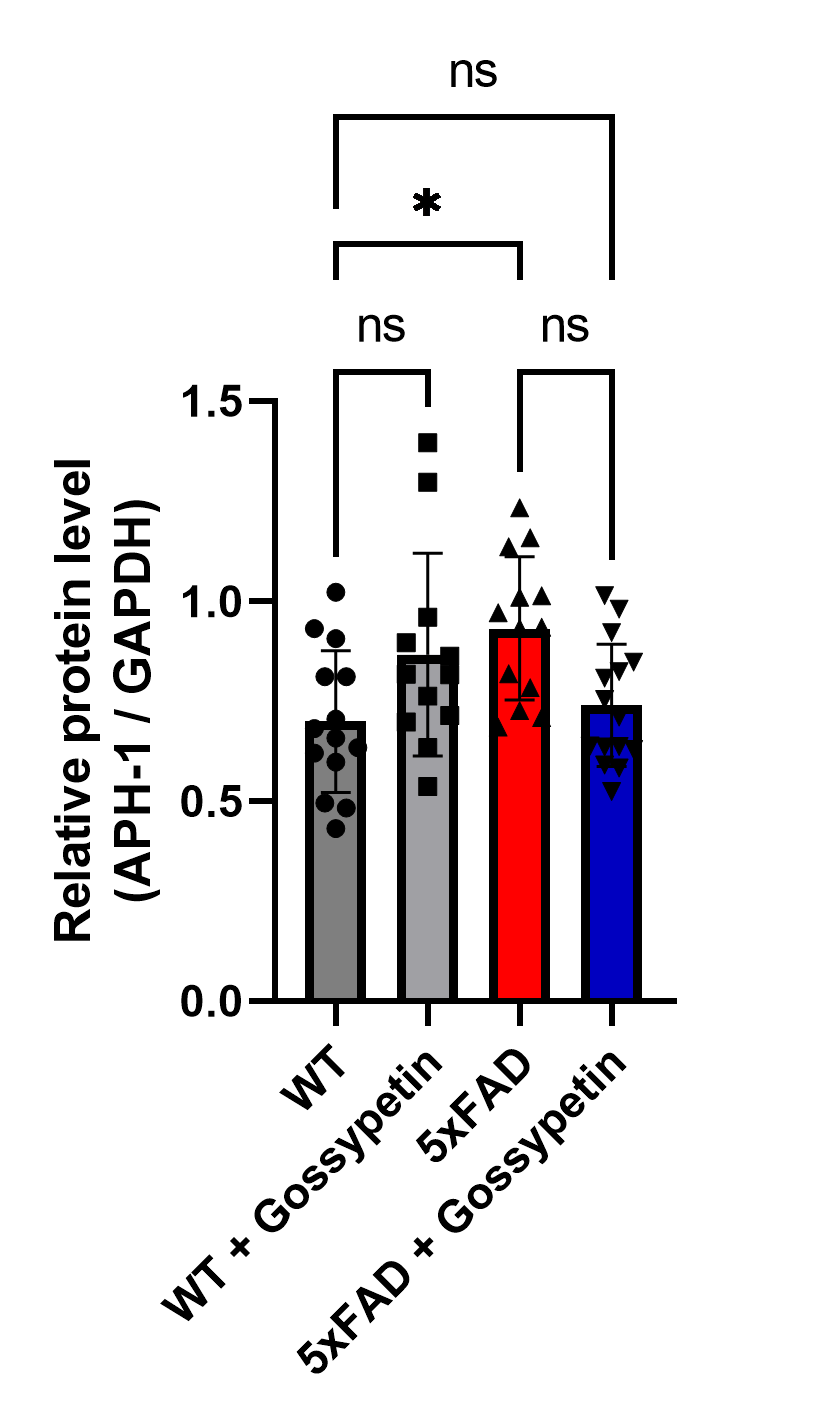

Supplement: Supplementary file 6 — Additional file 6: Fig.S1 Gossypetin does not affect expression of β-, and γ-secretases and activity of β-secretase. (A to G) Time dependent β-secretase activity of mouse hippocampal lysate was measured with Relative Fluorescence Unit (RFU). Fluorescence excitation and emission wavelength was 335 nm and 495 nm respectively (A). Bar graph of RFU at each time point of 10 min (B), 20 min (C), 30 min (D), 40 min (E), 50 min (F), 60 min (G). (n = 10~12 mice per group) (H to L) Representative images of Western blot analysis for β-, γ-secretase subunits, and GAPDH (H). Bar graphs represent relative protein expression levels of BACE1 (I), Nicastrin (J), APH-1 (K), and PEN2 (L). (n = 12~15 mice per group) (M to P) Bar graphs represent relative mRNA expression level of β-, and γ-secretase subunits bace1 (M), ncstn (N), aph1 (O), pen2 (P). (n = 9~10 mice per group) Error bars represent the mean ± SD, *p < 0.05, ns = not significant, two-way ANOVA followed by Tukey’s multiple comparisons test. Fig. S2 Cell type classification of brain samples. (A) UMAP plot showing all cells from the brain samples, colored by their cell types. (B) Heatmap illustrating the Z-scores of average normalized expressions of cell type markers. (C) Violin plots displaying the log-scaled number of detected genes (top), Unique Molecular Identifiers (UMIs) (middle), and the percentage of mitochondrial gene expressions (bottom) per cell for each cell type. (D) UMAP plots showing all cells from the brain samples, colored by their sampled region (left), mouse strain (middle), or drug administration (right) condition. Fig. S3 Detailed subtyping of the microglial population. (A) UMAP plots showing all microglial cells from cortex region. The cells are colored by their celltypes (left). Heatmap showing the Z-scores of average normalized expressions of representative DEGs for each cell type from cortex region (right). (B) UMAP plots showing microglial cells from cortex (left) or hippocampus (right), colored by co [file 13195_2022_1096_MOESM6_ESM.zip › Figure S1K.png]

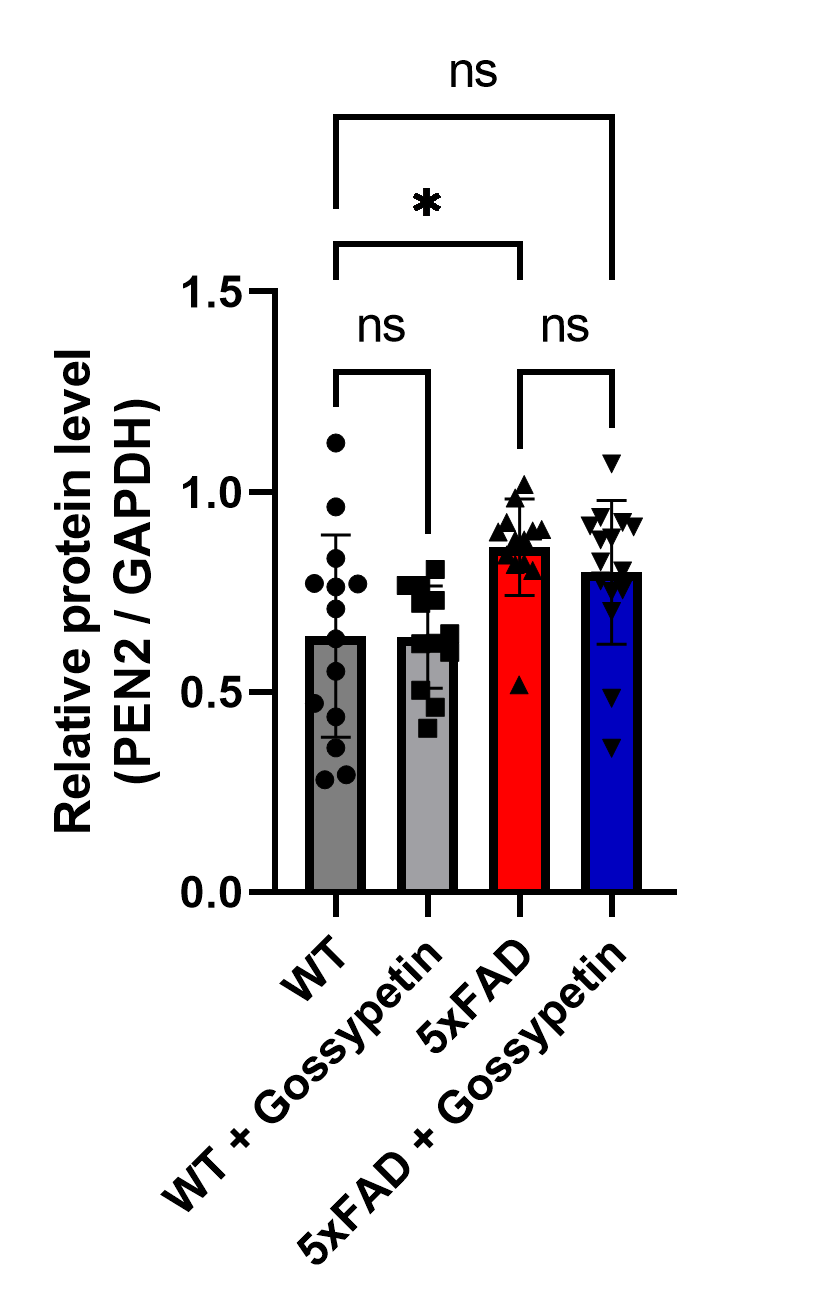

Supplement: Supplementary file 6 — Additional file 6: Fig.S1 Gossypetin does not affect expression of β-, and γ-secretases and activity of β-secretase. (A to G) Time dependent β-secretase activity of mouse hippocampal lysate was measured with Relative Fluorescence Unit (RFU). Fluorescence excitation and emission wavelength was 335 nm and 495 nm respectively (A). Bar graph of RFU at each time point of 10 min (B), 20 min (C), 30 min (D), 40 min (E), 50 min (F), 60 min (G). (n = 10~12 mice per group) (H to L) Representative images of Western blot analysis for β-, γ-secretase subunits, and GAPDH (H). Bar graphs represent relative protein expression levels of BACE1 (I), Nicastrin (J), APH-1 (K), and PEN2 (L). (n = 12~15 mice per group) (M to P) Bar graphs represent relative mRNA expression level of β-, and γ-secretase subunits bace1 (M), ncstn (N), aph1 (O), pen2 (P). (n = 9~10 mice per group) Error bars represent the mean ± SD, *p < 0.05, ns = not significant, two-way ANOVA followed by Tukey’s multiple comparisons test. Fig. S2 Cell type classification of brain samples. (A) UMAP plot showing all cells from the brain samples, colored by their cell types. (B) Heatmap illustrating the Z-scores of average normalized expressions of cell type markers. (C) Violin plots displaying the log-scaled number of detected genes (top), Unique Molecular Identifiers (UMIs) (middle), and the percentage of mitochondrial gene expressions (bottom) per cell for each cell type. (D) UMAP plots showing all cells from the brain samples, colored by their sampled region (left), mouse strain (middle), or drug administration (right) condition. Fig. S3 Detailed subtyping of the microglial population. (A) UMAP plots showing all microglial cells from cortex region. The cells are colored by their celltypes (left). Heatmap showing the Z-scores of average normalized expressions of representative DEGs for each cell type from cortex region (right). (B) UMAP plots showing microglial cells from cortex (left) or hippocampus (right), colored by co [file 13195_2022_1096_MOESM6_ESM.zip › Figure S1L.png]

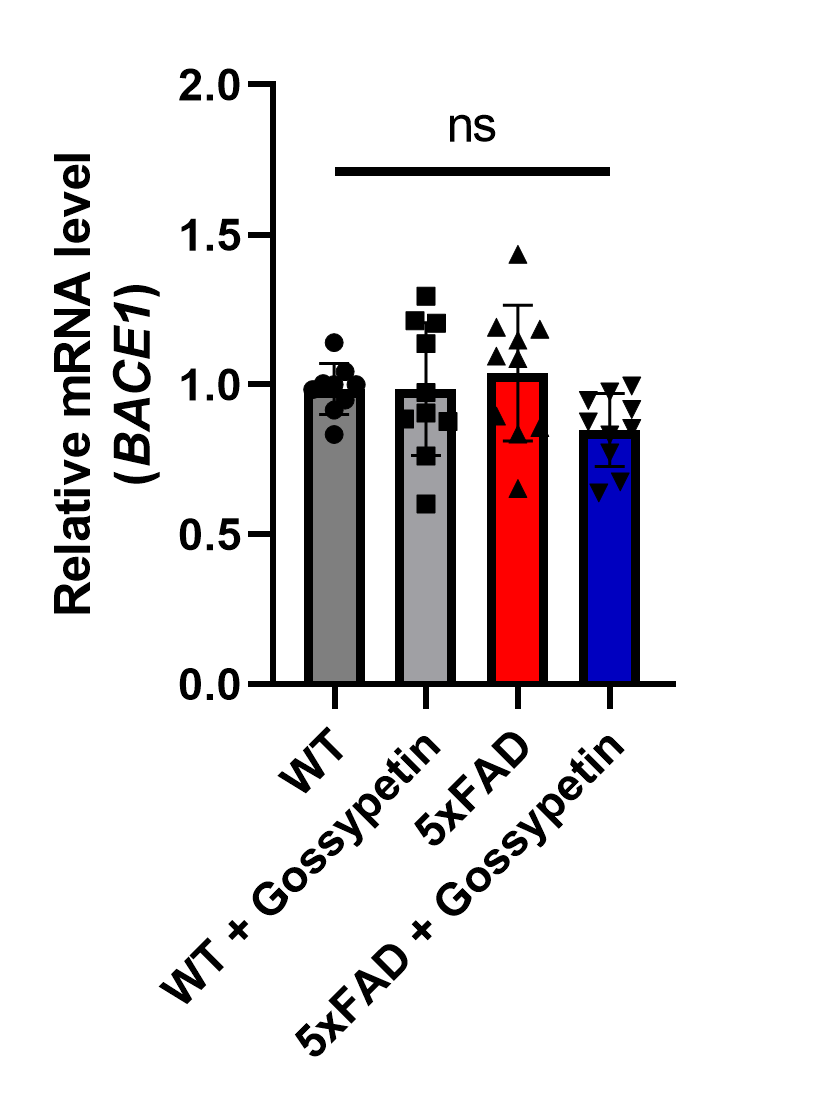

Supplement: Supplementary file 6 — Additional file 6: Fig.S1 Gossypetin does not affect expression of β-, and γ-secretases and activity of β-secretase. (A to G) Time dependent β-secretase activity of mouse hippocampal lysate was measured with Relative Fluorescence Unit (RFU). Fluorescence excitation and emission wavelength was 335 nm and 495 nm respectively (A). Bar graph of RFU at each time point of 10 min (B), 20 min (C), 30 min (D), 40 min (E), 50 min (F), 60 min (G). (n = 10~12 mice per group) (H to L) Representative images of Western blot analysis for β-, γ-secretase subunits, and GAPDH (H). Bar graphs represent relative protein expression levels of BACE1 (I), Nicastrin (J), APH-1 (K), and PEN2 (L). (n = 12~15 mice per group) (M to P) Bar graphs represent relative mRNA expression level of β-, and γ-secretase subunits bace1 (M), ncstn (N), aph1 (O), pen2 (P). (n = 9~10 mice per group) Error bars represent the mean ± SD, *p < 0.05, ns = not significant, two-way ANOVA followed by Tukey’s multiple comparisons test. Fig. S2 Cell type classification of brain samples. (A) UMAP plot showing all cells from the brain samples, colored by their cell types. (B) Heatmap illustrating the Z-scores of average normalized expressions of cell type markers. (C) Violin plots displaying the log-scaled number of detected genes (top), Unique Molecular Identifiers (UMIs) (middle), and the percentage of mitochondrial gene expressions (bottom) per cell for each cell type. (D) UMAP plots showing all cells from the brain samples, colored by their sampled region (left), mouse strain (middle), or drug administration (right) condition. Fig. S3 Detailed subtyping of the microglial population. (A) UMAP plots showing all microglial cells from cortex region. The cells are colored by their celltypes (left). Heatmap showing the Z-scores of average normalized expressions of representative DEGs for each cell type from cortex region (right). (B) UMAP plots showing microglial cells from cortex (left) or hippocampus (right), colored by co [file 13195_2022_1096_MOESM6_ESM.zip › Figure S1M.png]

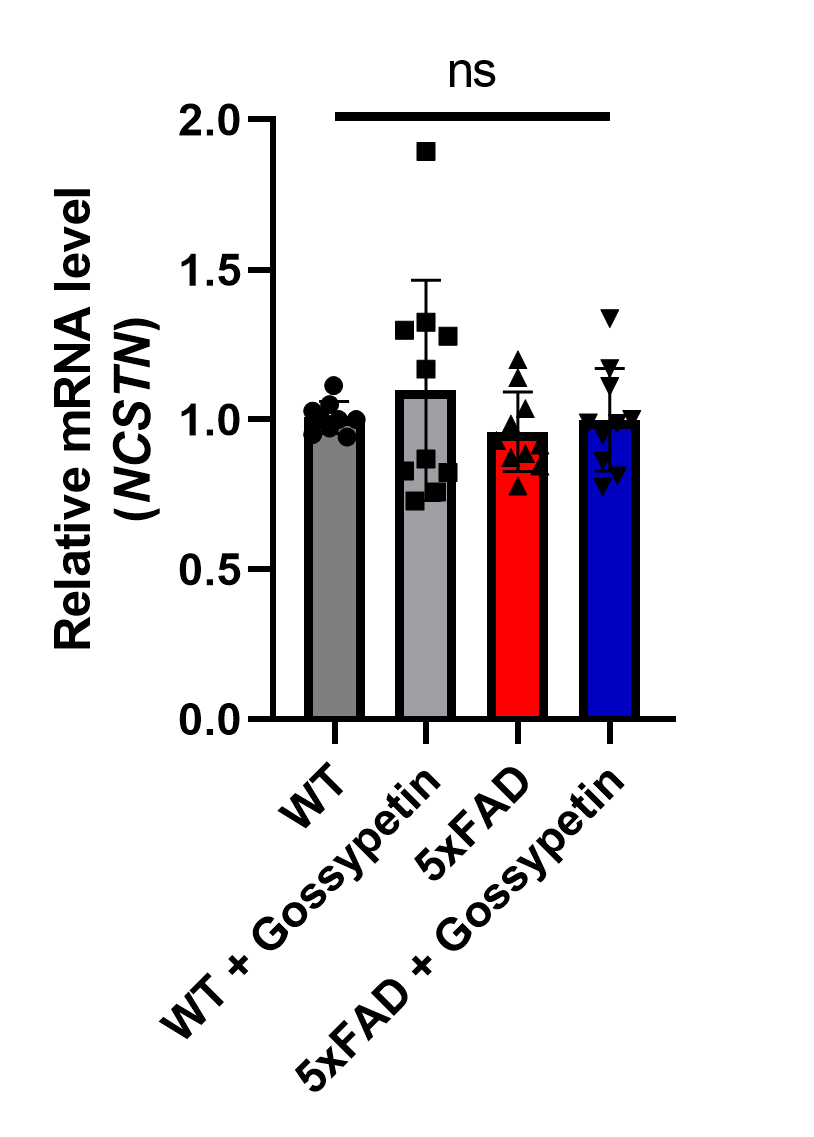

Supplement: Supplementary file 6 — Additional file 6: Fig.S1 Gossypetin does not affect expression of β-, and γ-secretases and activity of β-secretase. (A to G) Time dependent β-secretase activity of mouse hippocampal lysate was measured with Relative Fluorescence Unit (RFU). Fluorescence excitation and emission wavelength was 335 nm and 495 nm respectively (A). Bar graph of RFU at each time point of 10 min (B), 20 min (C), 30 min (D), 40 min (E), 50 min (F), 60 min (G). (n = 10~12 mice per group) (H to L) Representative images of Western blot analysis for β-, γ-secretase subunits, and GAPDH (H). Bar graphs represent relative protein expression levels of BACE1 (I), Nicastrin (J), APH-1 (K), and PEN2 (L). (n = 12~15 mice per group) (M to P) Bar graphs represent relative mRNA expression level of β-, and γ-secretase subunits bace1 (M), ncstn (N), aph1 (O), pen2 (P). (n = 9~10 mice per group) Error bars represent the mean ± SD, *p < 0.05, ns = not significant, two-way ANOVA followed by Tukey’s multiple comparisons test. Fig. S2 Cell type classification of brain samples. (A) UMAP plot showing all cells from the brain samples, colored by their cell types. (B) Heatmap illustrating the Z-scores of average normalized expressions of cell type markers. (C) Violin plots displaying the log-scaled number of detected genes (top), Unique Molecular Identifiers (UMIs) (middle), and the percentage of mitochondrial gene expressions (bottom) per cell for each cell type. (D) UMAP plots showing all cells from the brain samples, colored by their sampled region (left), mouse strain (middle), or drug administration (right) condition. Fig. S3 Detailed subtyping of the microglial population. (A) UMAP plots showing all microglial cells from cortex region. The cells are colored by their celltypes (left). Heatmap showing the Z-scores of average normalized expressions of representative DEGs for each cell type from cortex region (right). (B) UMAP plots showing microglial cells from cortex (left) or hippocampus (right), colored by co [file 13195_2022_1096_MOESM6_ESM.zip › Figure S1N.png]

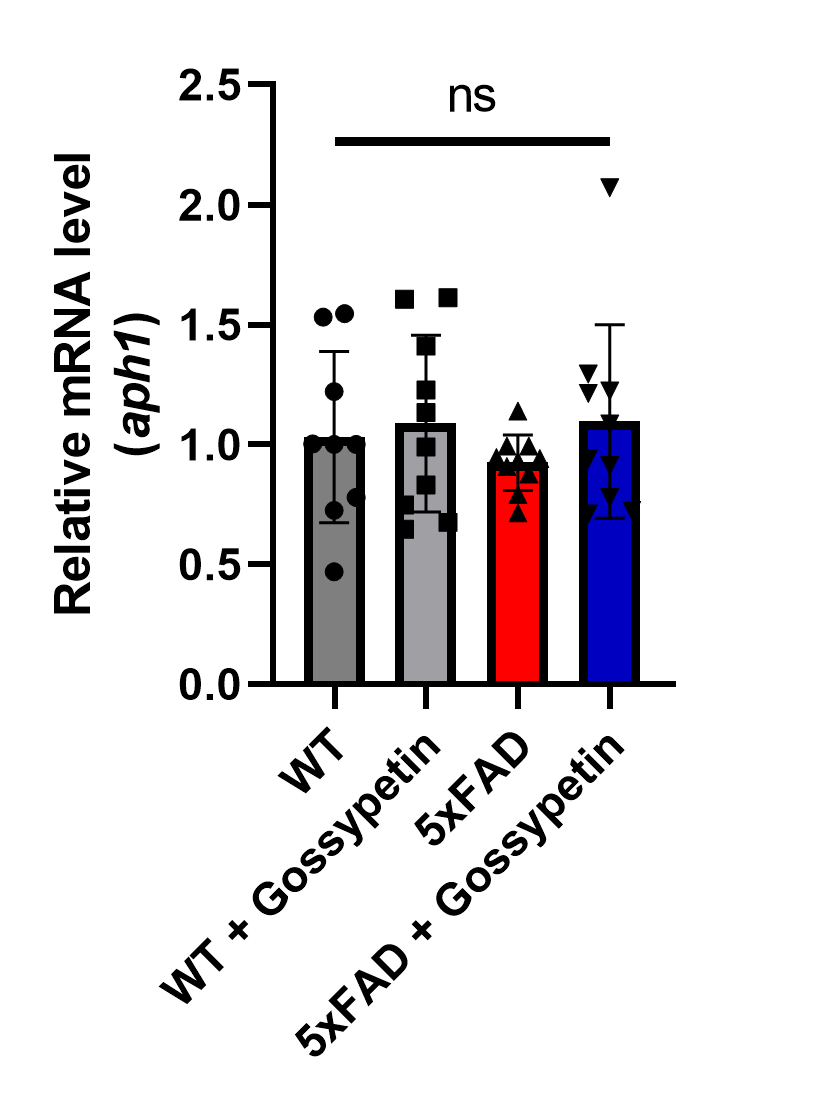

Supplement: Supplementary file 6 — Additional file 6: Fig.S1 Gossypetin does not affect expression of β-, and γ-secretases and activity of β-secretase. (A to G) Time dependent β-secretase activity of mouse hippocampal lysate was measured with Relative Fluorescence Unit (RFU). Fluorescence excitation and emission wavelength was 335 nm and 495 nm respectively (A). Bar graph of RFU at each time point of 10 min (B), 20 min (C), 30 min (D), 40 min (E), 50 min (F), 60 min (G). (n = 10~12 mice per group) (H to L) Representative images of Western blot analysis for β-, γ-secretase subunits, and GAPDH (H). Bar graphs represent relative protein expression levels of BACE1 (I), Nicastrin (J), APH-1 (K), and PEN2 (L). (n = 12~15 mice per group) (M to P) Bar graphs represent relative mRNA expression level of β-, and γ-secretase subunits bace1 (M), ncstn (N), aph1 (O), pen2 (P). (n = 9~10 mice per group) Error bars represent the mean ± SD, *p < 0.05, ns = not significant, two-way ANOVA followed by Tukey’s multiple comparisons test. Fig. S2 Cell type classification of brain samples. (A) UMAP plot showing all cells from the brain samples, colored by their cell types. (B) Heatmap illustrating the Z-scores of average normalized expressions of cell type markers. (C) Violin plots displaying the log-scaled number of detected genes (top), Unique Molecular Identifiers (UMIs) (middle), and the percentage of mitochondrial gene expressions (bottom) per cell for each cell type. (D) UMAP plots showing all cells from the brain samples, colored by their sampled region (left), mouse strain (middle), or drug administration (right) condition. Fig. S3 Detailed subtyping of the microglial population. (A) UMAP plots showing all microglial cells from cortex region. The cells are colored by their celltypes (left). Heatmap showing the Z-scores of average normalized expressions of representative DEGs for each cell type from cortex region (right). (B) UMAP plots showing microglial cells from cortex (left) or hippocampus (right), colored by co [file 13195_2022_1096_MOESM6_ESM.zip › Figure S1O.png]

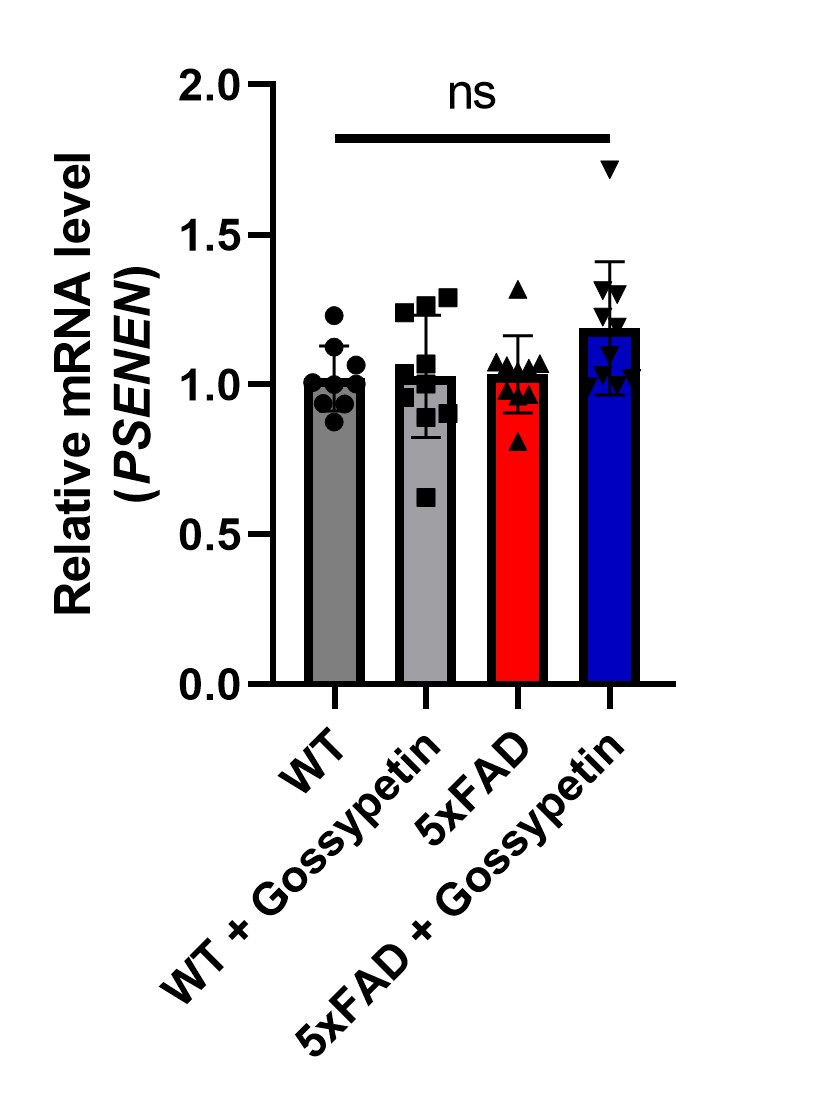

Supplement: Supplementary file 6 — Additional file 6: Fig.S1 Gossypetin does not affect expression of β-, and γ-secretases and activity of β-secretase. (A to G) Time dependent β-secretase activity of mouse hippocampal lysate was measured with Relative Fluorescence Unit (RFU). Fluorescence excitation and emission wavelength was 335 nm and 495 nm respectively (A). Bar graph of RFU at each time point of 10 min (B), 20 min (C), 30 min (D), 40 min (E), 50 min (F), 60 min (G). (n = 10~12 mice per group) (H to L) Representative images of Western blot analysis for β-, γ-secretase subunits, and GAPDH (H). Bar graphs represent relative protein expression levels of BACE1 (I), Nicastrin (J), APH-1 (K), and PEN2 (L). (n = 12~15 mice per group) (M to P) Bar graphs represent relative mRNA expression level of β-, and γ-secretase subunits bace1 (M), ncstn (N), aph1 (O), pen2 (P). (n = 9~10 mice per group) Error bars represent the mean ± SD, *p < 0.05, ns = not significant, two-way ANOVA followed by Tukey’s multiple comparisons test. Fig. S2 Cell type classification of brain samples. (A) UMAP plot showing all cells from the brain samples, colored by their cell types. (B) Heatmap illustrating the Z-scores of average normalized expressions of cell type markers. (C) Violin plots displaying the log-scaled number of detected genes (top), Unique Molecular Identifiers (UMIs) (middle), and the percentage of mitochondrial gene expressions (bottom) per cell for each cell type. (D) UMAP plots showing all cells from the brain samples, colored by their sampled region (left), mouse strain (middle), or drug administration (right) condition. Fig. S3 Detailed subtyping of the microglial population. (A) UMAP plots showing all microglial cells from cortex region. The cells are colored by their celltypes (left). Heatmap showing the Z-scores of average normalized expressions of representative DEGs for each cell type from cortex region (right). (B) UMAP plots showing microglial cells from cortex (left) or hippocampus (right), colored by co [file 13195_2022_1096_MOESM6_ESM.zip › Figure S1P.png]

Figure S2. Cell type classification of brain samples

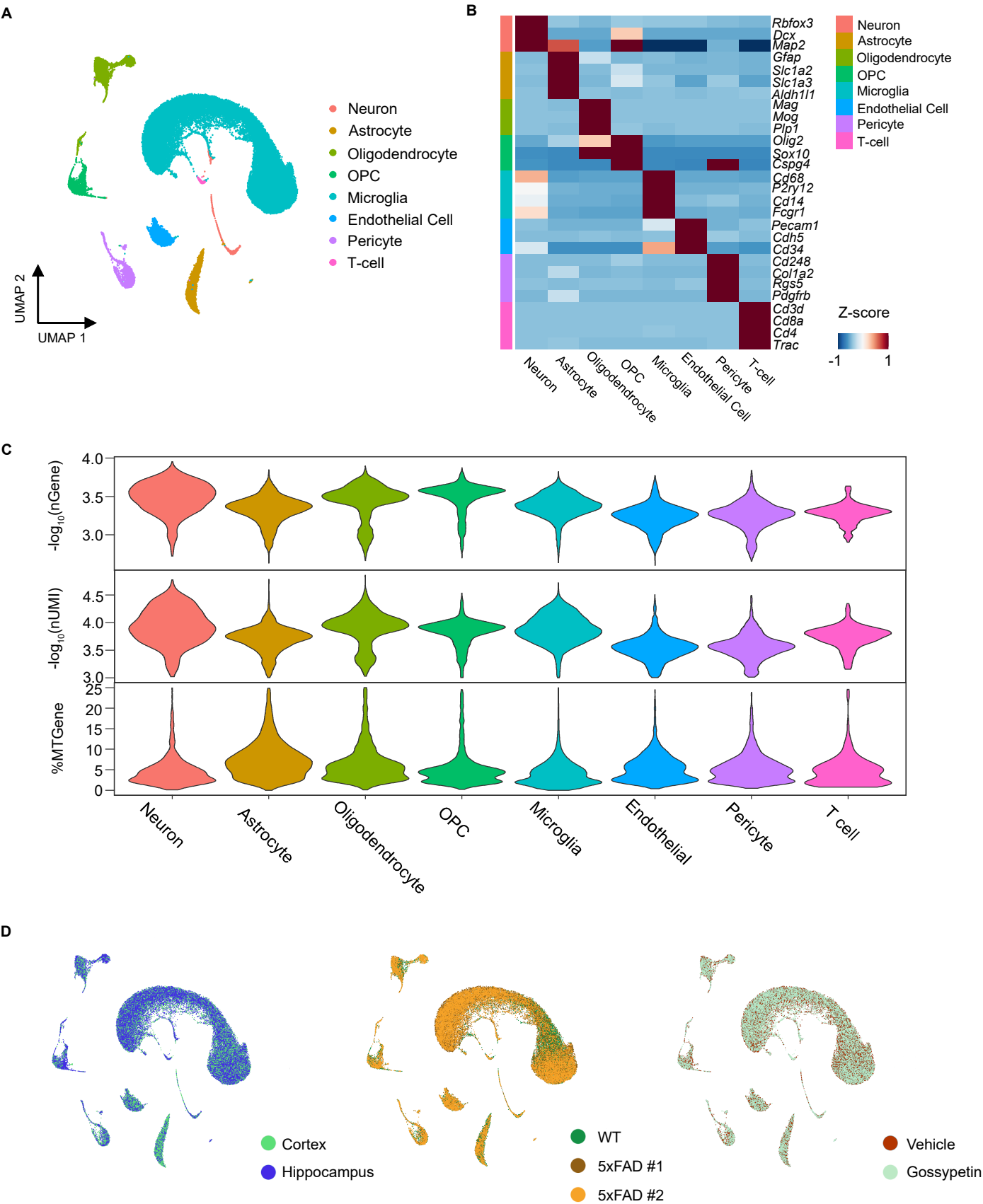

Supplement: Supplementary file 6 — Additional file 6: Fig.S1 Gossypetin does not affect expression of β-, and γ-secretases and activity of β-secretase. (A to G) Time dependent β-secretase activity of mouse hippocampal lysate was measured with Relative Fluorescence Unit (RFU). Fluorescence excitation and emission wavelength was 335 nm and 495 nm respectively (A). Bar graph of RFU at each time point of 10 min (B), 20 min (C), 30 min (D), 40 min (E), 50 min (F), 60 min (G). (n = 10~12 mice per group) (H to L) Representative images of Western blot analysis for β-, γ-secretase subunits, and GAPDH (H). Bar graphs represent relative protein expression levels of BACE1 (I), Nicastrin (J), APH-1 (K), and PEN2 (L). (n = 12~15 mice per group) (M to P) Bar graphs represent relative mRNA expression level of β-, and γ-secretase subunits bace1 (M), ncstn (N), aph1 (O), pen2 (P). (n = 9~10 mice per group) Error bars represent the mean ± SD, *p < 0.05, ns = not significant, two-way ANOVA followed by Tukey’s multiple comparisons test. Fig. S2 Cell type classification of brain samples. (A) UMAP plot showing all cells from the brain samples, colored by their cell types. (B) Heatmap illustrating the Z-scores of average normalized expressions of cell type markers. (C) Violin plots displaying the log-scaled number of detected genes (top), Unique Molecular Identifiers (UMIs) (middle), and the percentage of mitochondrial gene expressions (bottom) per cell for each cell type. (D) UMAP plots showing all cells from the brain samples, colored by their sampled region (left), mouse strain (middle), or drug administration (right) condition. Fig. S3 Detailed subtyping of the microglial population. (A) UMAP plots showing all microglial cells from cortex region. The cells are colored by their celltypes (left). Heatmap showing the Z-scores of average normalized expressions of representative DEGs for each cell type from cortex region (right). (B) UMAP plots showing microglial cells from cortex (left) or hippocampus (right), colored by co [file 13195_2022_1096_MOESM6_ESM.zip › Figure S2 (Total).pdf]

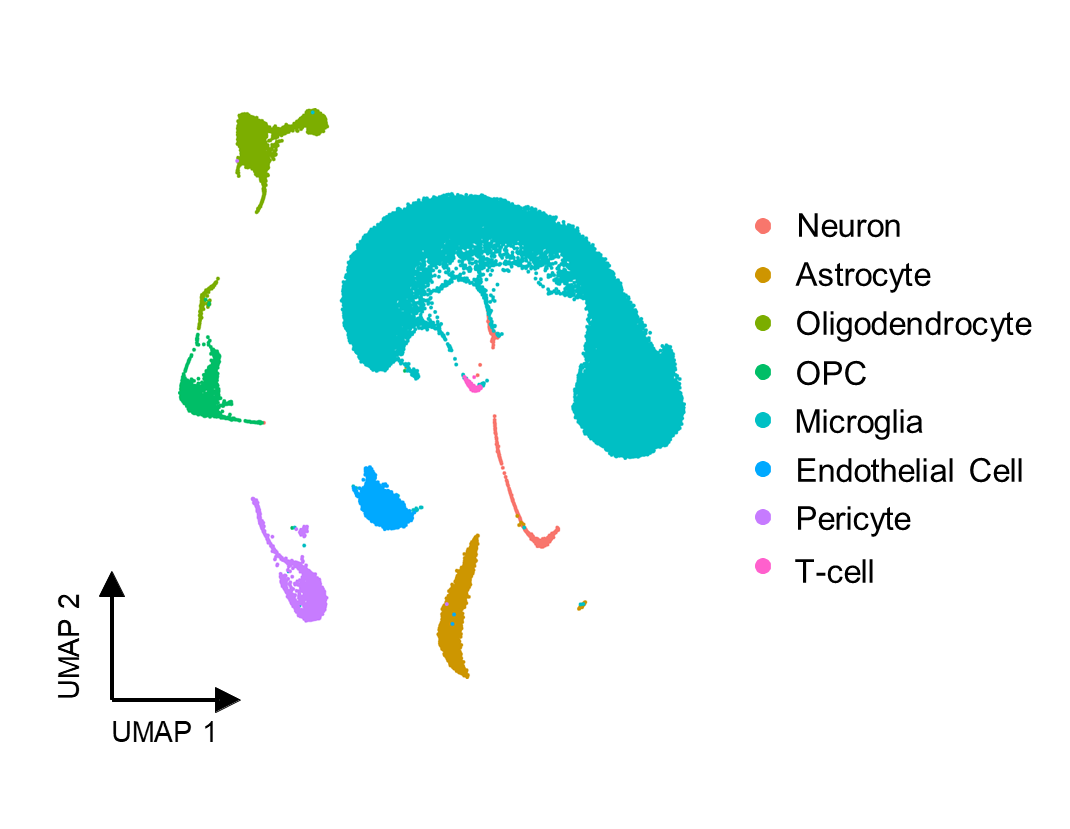

Supplement: Supplementary file 6 — Additional file 6: Fig.S1 Gossypetin does not affect expression of β-, and γ-secretases and activity of β-secretase. (A to G) Time dependent β-secretase activity of mouse hippocampal lysate was measured with Relative Fluorescence Unit (RFU). Fluorescence excitation and emission wavelength was 335 nm and 495 nm respectively (A). Bar graph of RFU at each time point of 10 min (B), 20 min (C), 30 min (D), 40 min (E), 50 min (F), 60 min (G). (n = 10~12 mice per group) (H to L) Representative images of Western blot analysis for β-, γ-secretase subunits, and GAPDH (H). Bar graphs represent relative protein expression levels of BACE1 (I), Nicastrin (J), APH-1 (K), and PEN2 (L). (n = 12~15 mice per group) (M to P) Bar graphs represent relative mRNA expression level of β-, and γ-secretase subunits bace1 (M), ncstn (N), aph1 (O), pen2 (P). (n = 9~10 mice per group) Error bars represent the mean ± SD, *p < 0.05, ns = not significant, two-way ANOVA followed by Tukey’s multiple comparisons test. Fig. S2 Cell type classification of brain samples. (A) UMAP plot showing all cells from the brain samples, colored by their cell types. (B) Heatmap illustrating the Z-scores of average normalized expressions of cell type markers. (C) Violin plots displaying the log-scaled number of detected genes (top), Unique Molecular Identifiers (UMIs) (middle), and the percentage of mitochondrial gene expressions (bottom) per cell for each cell type. (D) UMAP plots showing all cells from the brain samples, colored by their sampled region (left), mouse strain (middle), or drug administration (right) condition. Fig. S3 Detailed subtyping of the microglial population. (A) UMAP plots showing all microglial cells from cortex region. The cells are colored by their celltypes (left). Heatmap showing the Z-scores of average normalized expressions of representative DEGs for each cell type from cortex region (right). (B) UMAP plots showing microglial cells from cortex (left) or hippocampus (right), colored by co [file 13195_2022_1096_MOESM6_ESM.zip › Figure S2A.png]

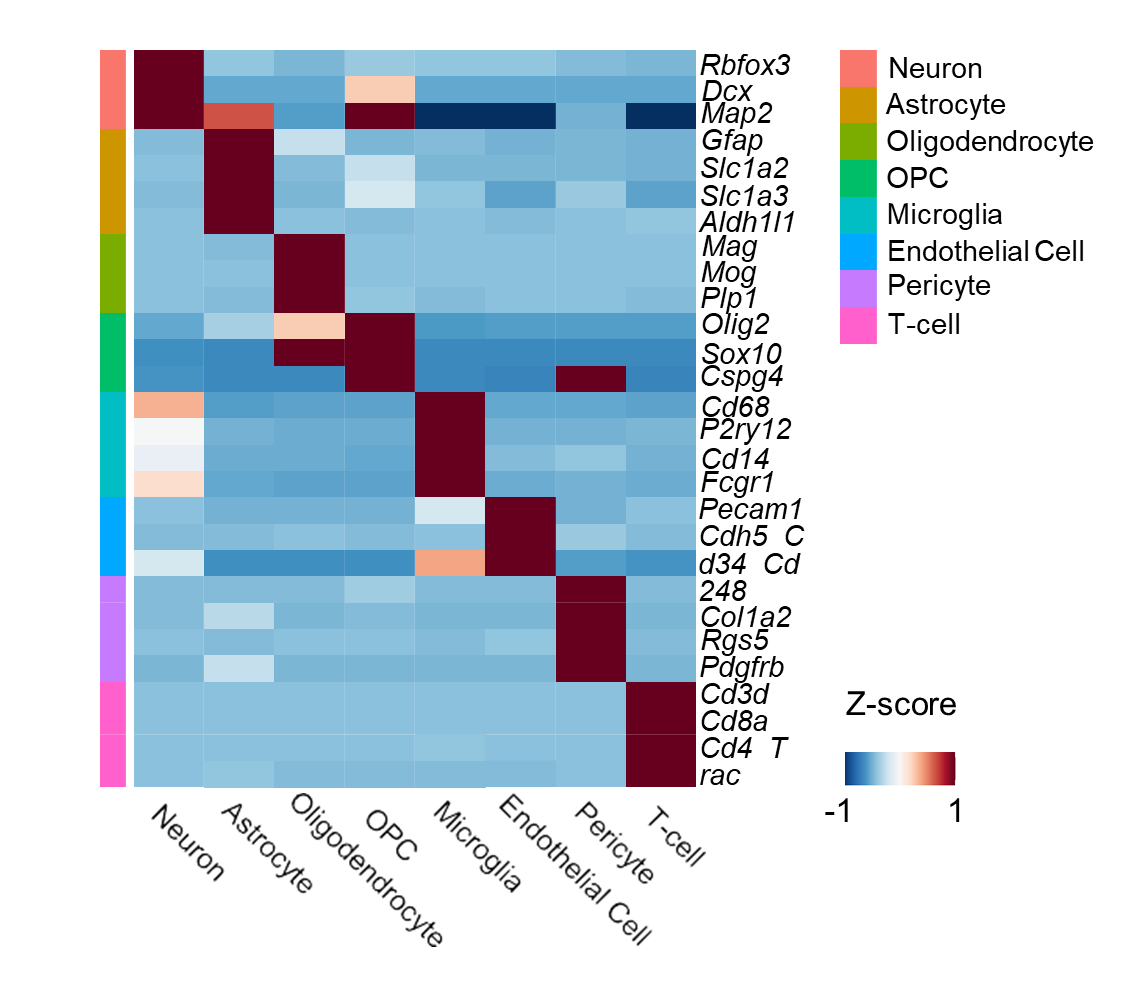

Supplement: Supplementary file 6 — Additional file 6: Fig.S1 Gossypetin does not affect expression of β-, and γ-secretases and activity of β-secretase. (A to G) Time dependent β-secretase activity of mouse hippocampal lysate was measured with Relative Fluorescence Unit (RFU). Fluorescence excitation and emission wavelength was 335 nm and 495 nm respectively (A). Bar graph of RFU at each time point of 10 min (B), 20 min (C), 30 min (D), 40 min (E), 50 min (F), 60 min (G). (n = 10~12 mice per group) (H to L) Representative images of Western blot analysis for β-, γ-secretase subunits, and GAPDH (H). Bar graphs represent relative protein expression levels of BACE1 (I), Nicastrin (J), APH-1 (K), and PEN2 (L). (n = 12~15 mice per group) (M to P) Bar graphs represent relative mRNA expression level of β-, and γ-secretase subunits bace1 (M), ncstn (N), aph1 (O), pen2 (P). (n = 9~10 mice per group) Error bars represent the mean ± SD, *p < 0.05, ns = not significant, two-way ANOVA followed by Tukey’s multiple comparisons test. Fig. S2 Cell type classification of brain samples. (A) UMAP plot showing all cells from the brain samples, colored by their cell types. (B) Heatmap illustrating the Z-scores of average normalized expressions of cell type markers. (C) Violin plots displaying the log-scaled number of detected genes (top), Unique Molecular Identifiers (UMIs) (middle), and the percentage of mitochondrial gene expressions (bottom) per cell for each cell type. (D) UMAP plots showing all cells from the brain samples, colored by their sampled region (left), mouse strain (middle), or drug administration (right) condition. Fig. S3 Detailed subtyping of the microglial population. (A) UMAP plots showing all microglial cells from cortex region. The cells are colored by their celltypes (left). Heatmap showing the Z-scores of average normalized expressions of representative DEGs for each cell type from cortex region (right). (B) UMAP plots showing microglial cells from cortex (left) or hippocampus (right), colored by co [file 13195_2022_1096_MOESM6_ESM.zip › Figure S2B.png]

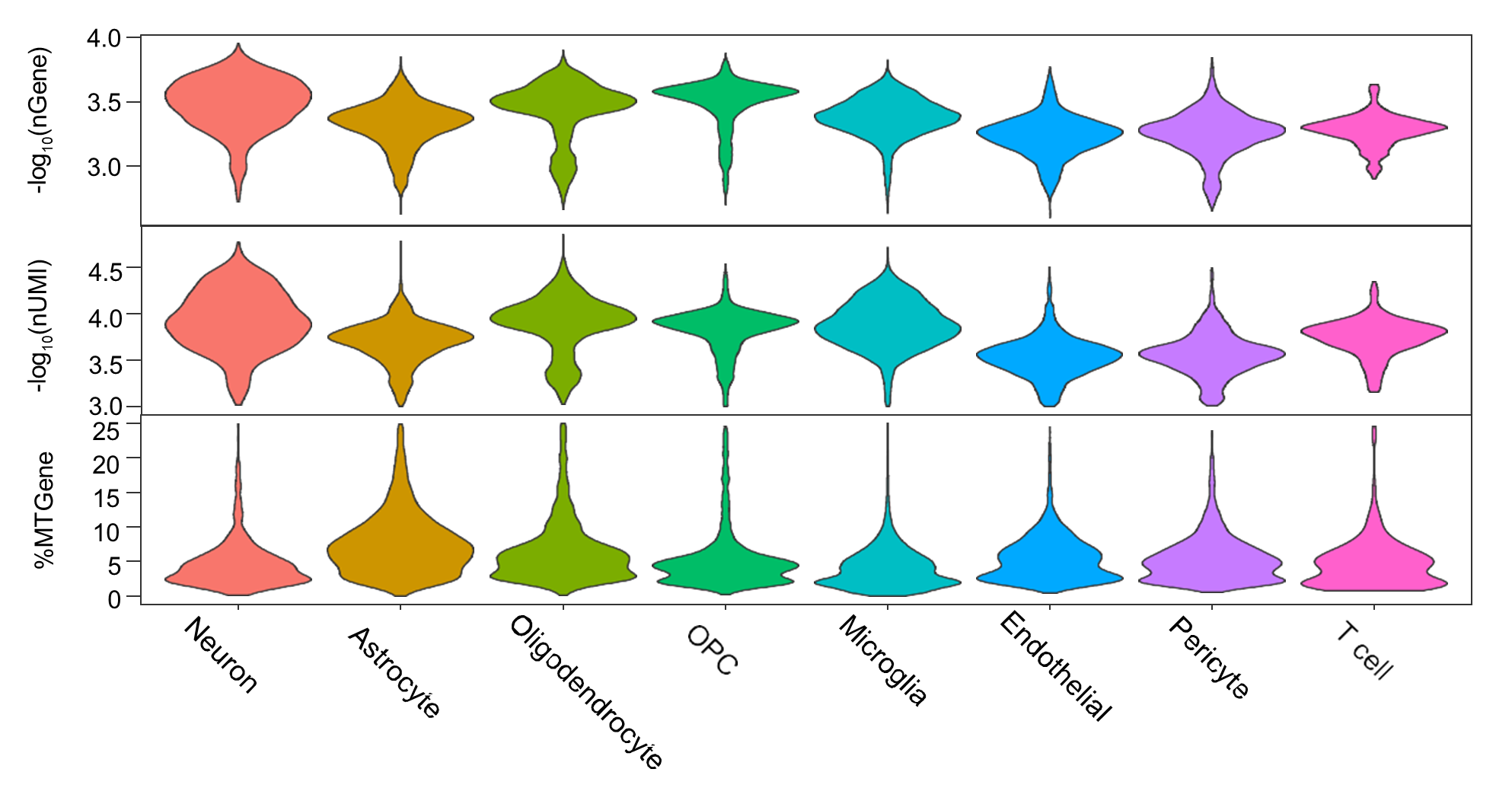

Supplement: Supplementary file 6 — Additional file 6: Fig.S1 Gossypetin does not affect expression of β-, and γ-secretases and activity of β-secretase. (A to G) Time dependent β-secretase activity of mouse hippocampal lysate was measured with Relative Fluorescence Unit (RFU). Fluorescence excitation and emission wavelength was 335 nm and 495 nm respectively (A). Bar graph of RFU at each time point of 10 min (B), 20 min (C), 30 min (D), 40 min (E), 50 min (F), 60 min (G). (n = 10~12 mice per group) (H to L) Representative images of Western blot analysis for β-, γ-secretase subunits, and GAPDH (H). Bar graphs represent relative protein expression levels of BACE1 (I), Nicastrin (J), APH-1 (K), and PEN2 (L). (n = 12~15 mice per group) (M to P) Bar graphs represent relative mRNA expression level of β-, and γ-secretase subunits bace1 (M), ncstn (N), aph1 (O), pen2 (P). (n = 9~10 mice per group) Error bars represent the mean ± SD, *p < 0.05, ns = not significant, two-way ANOVA followed by Tukey’s multiple comparisons test. Fig. S2 Cell type classification of brain samples. (A) UMAP plot showing all cells from the brain samples, colored by their cell types. (B) Heatmap illustrating the Z-scores of average normalized expressions of cell type markers. (C) Violin plots displaying the log-scaled number of detected genes (top), Unique Molecular Identifiers (UMIs) (middle), and the percentage of mitochondrial gene expressions (bottom) per cell for each cell type. (D) UMAP plots showing all cells from the brain samples, colored by their sampled region (left), mouse strain (middle), or drug administration (right) condition. Fig. S3 Detailed subtyping of the microglial population. (A) UMAP plots showing all microglial cells from cortex region. The cells are colored by their celltypes (left). Heatmap showing the Z-scores of average normalized expressions of representative DEGs for each cell type from cortex region (right). (B) UMAP plots showing microglial cells from cortex (left) or hippocampus (right), colored by co [file 13195_2022_1096_MOESM6_ESM.zip › Figure S2C.png]

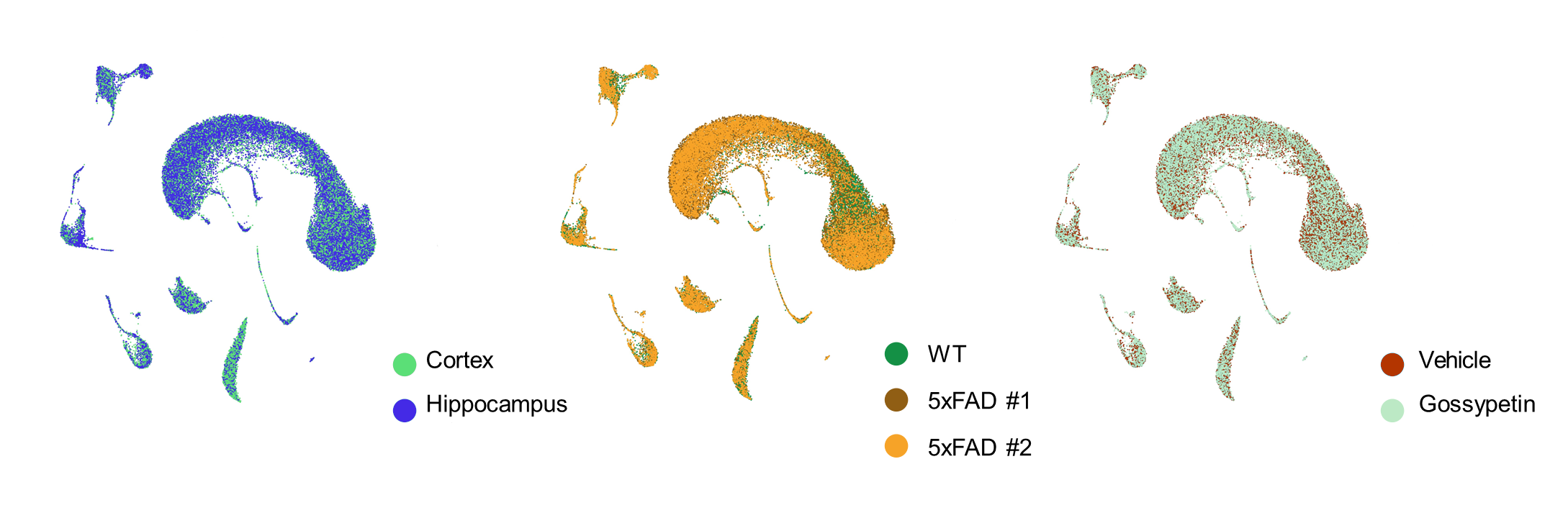

Supplement: Supplementary file 6 — Additional file 6: Fig.S1 Gossypetin does not affect expression of β-, and γ-secretases and activity of β-secretase. (A to G) Time dependent β-secretase activity of mouse hippocampal lysate was measured with Relative Fluorescence Unit (RFU). Fluorescence excitation and emission wavelength was 335 nm and 495 nm respectively (A). Bar graph of RFU at each time point of 10 min (B), 20 min (C), 30 min (D), 40 min (E), 50 min (F), 60 min (G). (n = 10~12 mice per group) (H to L) Representative images of Western blot analysis for β-, γ-secretase subunits, and GAPDH (H). Bar graphs represent relative protein expression levels of BACE1 (I), Nicastrin (J), APH-1 (K), and PEN2 (L). (n = 12~15 mice per group) (M to P) Bar graphs represent relative mRNA expression level of β-, and γ-secretase subunits bace1 (M), ncstn (N), aph1 (O), pen2 (P). (n = 9~10 mice per group) Error bars represent the mean ± SD, *p < 0.05, ns = not significant, two-way ANOVA followed by Tukey’s multiple comparisons test. Fig. S2 Cell type classification of brain samples. (A) UMAP plot showing all cells from the brain samples, colored by their cell types. (B) Heatmap illustrating the Z-scores of average normalized expressions of cell type markers. (C) Violin plots displaying the log-scaled number of detected genes (top), Unique Molecular Identifiers (UMIs) (middle), and the percentage of mitochondrial gene expressions (bottom) per cell for each cell type. (D) UMAP plots showing all cells from the brain samples, colored by their sampled region (left), mouse strain (middle), or drug administration (right) condition. Fig. S3 Detailed subtyping of the microglial population. (A) UMAP plots showing all microglial cells from cortex region. The cells are colored by their celltypes (left). Heatmap showing the Z-scores of average normalized expressions of representative DEGs for each cell type from cortex region (right). (B) UMAP plots showing microglial cells from cortex (left) or hippocampus (right), colored by co [file 13195_2022_1096_MOESM6_ESM.zip › Figure S2D.png]

Figure S3. Detailed subtyping of the microglial population

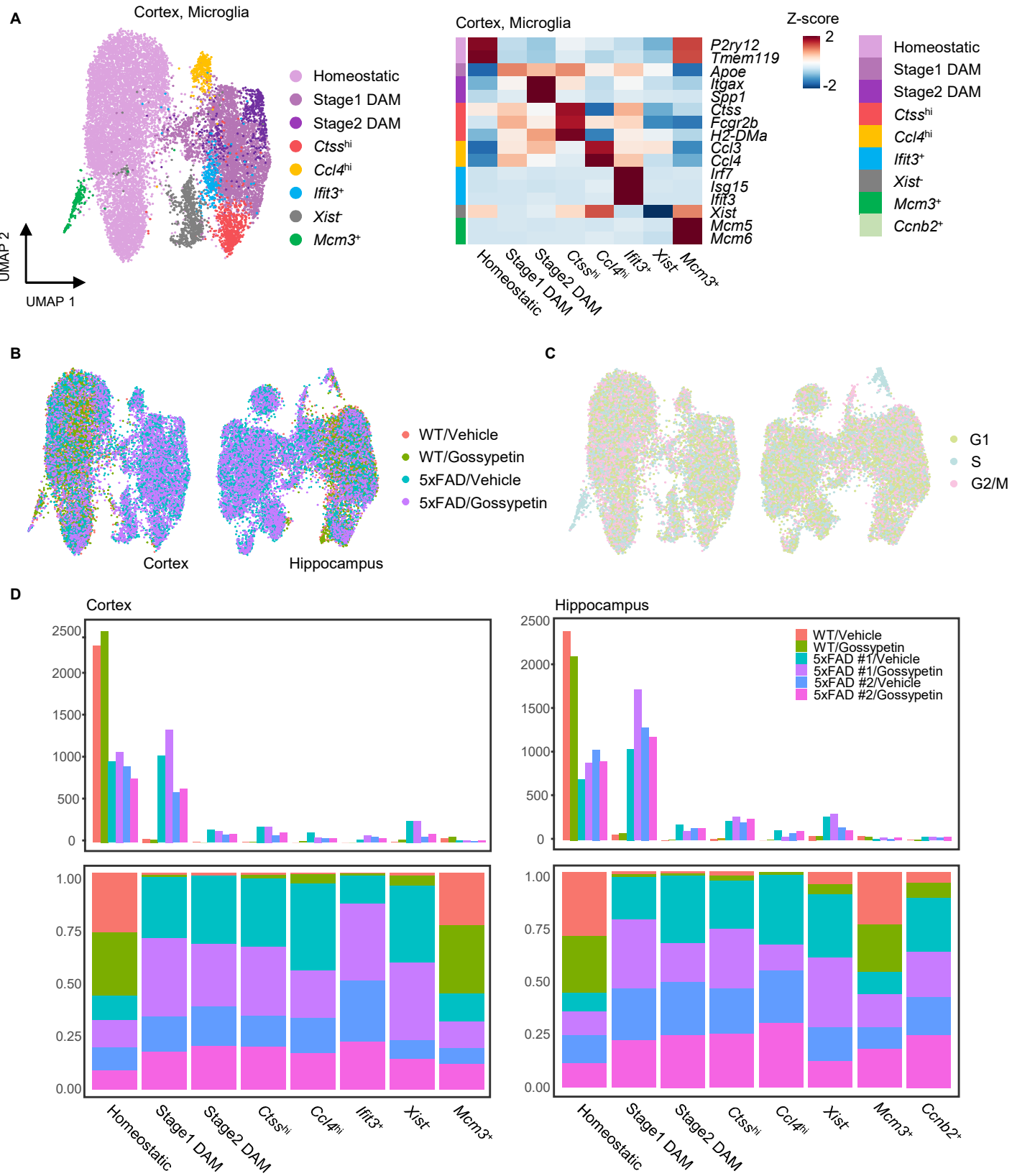

Supplement: Supplementary file 6 — Additional file 6: Fig.S1 Gossypetin does not affect expression of β-, and γ-secretases and activity of β-secretase. (A to G) Time dependent β-secretase activity of mouse hippocampal lysate was measured with Relative Fluorescence Unit (RFU). Fluorescence excitation and emission wavelength was 335 nm and 495 nm respectively (A). Bar graph of RFU at each time point of 10 min (B), 20 min (C), 30 min (D), 40 min (E), 50 min (F), 60 min (G). (n = 10~12 mice per group) (H to L) Representative images of Western blot analysis for β-, γ-secretase subunits, and GAPDH (H). Bar graphs represent relative protein expression levels of BACE1 (I), Nicastrin (J), APH-1 (K), and PEN2 (L). (n = 12~15 mice per group) (M to P) Bar graphs represent relative mRNA expression level of β-, and γ-secretase subunits bace1 (M), ncstn (N), aph1 (O), pen2 (P). (n = 9~10 mice per group) Error bars represent the mean ± SD, *p < 0.05, ns = not significant, two-way ANOVA followed by Tukey’s multiple comparisons test. Fig. S2 Cell type classification of brain samples. (A) UMAP plot showing all cells from the brain samples, colored by their cell types. (B) Heatmap illustrating the Z-scores of average normalized expressions of cell type markers. (C) Violin plots displaying the log-scaled number of detected genes (top), Unique Molecular Identifiers (UMIs) (middle), and the percentage of mitochondrial gene expressions (bottom) per cell for each cell type. (D) UMAP plots showing all cells from the brain samples, colored by their sampled region (left), mouse strain (middle), or drug administration (right) condition. Fig. S3 Detailed subtyping of the microglial population. (A) UMAP plots showing all microglial cells from cortex region. The cells are colored by their celltypes (left). Heatmap showing the Z-scores of average normalized expressions of representative DEGs for each cell type from cortex region (right). (B) UMAP plots showing microglial cells from cortex (left) or hippocampus (right), colored by co [file 13195_2022_1096_MOESM6_ESM.zip › Figure S3 (Total).pdf]

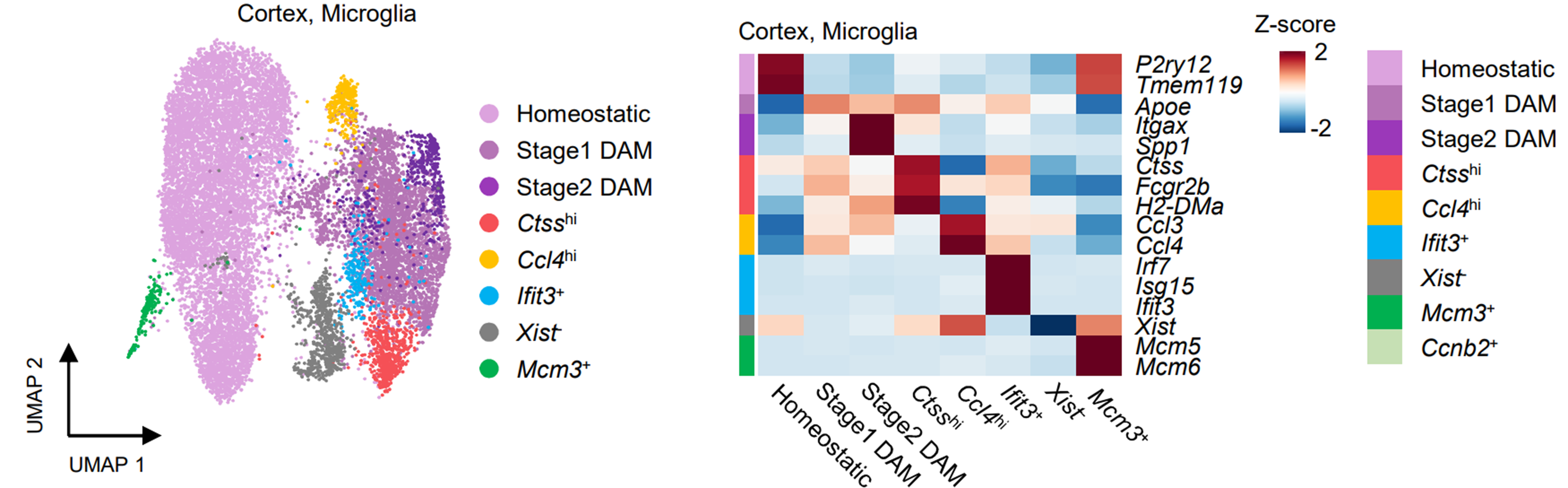

Supplement: Supplementary file 6 — Additional file 6: Fig.S1 Gossypetin does not affect expression of β-, and γ-secretases and activity of β-secretase. (A to G) Time dependent β-secretase activity of mouse hippocampal lysate was measured with Relative Fluorescence Unit (RFU). Fluorescence excitation and emission wavelength was 335 nm and 495 nm respectively (A). Bar graph of RFU at each time point of 10 min (B), 20 min (C), 30 min (D), 40 min (E), 50 min (F), 60 min (G). (n = 10~12 mice per group) (H to L) Representative images of Western blot analysis for β-, γ-secretase subunits, and GAPDH (H). Bar graphs represent relative protein expression levels of BACE1 (I), Nicastrin (J), APH-1 (K), and PEN2 (L). (n = 12~15 mice per group) (M to P) Bar graphs represent relative mRNA expression level of β-, and γ-secretase subunits bace1 (M), ncstn (N), aph1 (O), pen2 (P). (n = 9~10 mice per group) Error bars represent the mean ± SD, *p < 0.05, ns = not significant, two-way ANOVA followed by Tukey’s multiple comparisons test. Fig. S2 Cell type classification of brain samples. (A) UMAP plot showing all cells from the brain samples, colored by their cell types. (B) Heatmap illustrating the Z-scores of average normalized expressions of cell type markers. (C) Violin plots displaying the log-scaled number of detected genes (top), Unique Molecular Identifiers (UMIs) (middle), and the percentage of mitochondrial gene expressions (bottom) per cell for each cell type. (D) UMAP plots showing all cells from the brain samples, colored by their sampled region (left), mouse strain (middle), or drug administration (right) condition. Fig. S3 Detailed subtyping of the microglial population. (A) UMAP plots showing all microglial cells from cortex region. The cells are colored by their celltypes (left). Heatmap showing the Z-scores of average normalized expressions of representative DEGs for each cell type from cortex region (right). (B) UMAP plots showing microglial cells from cortex (left) or hippocampus (right), colored by co [file 13195_2022_1096_MOESM6_ESM.zip › Figure S3A.png]

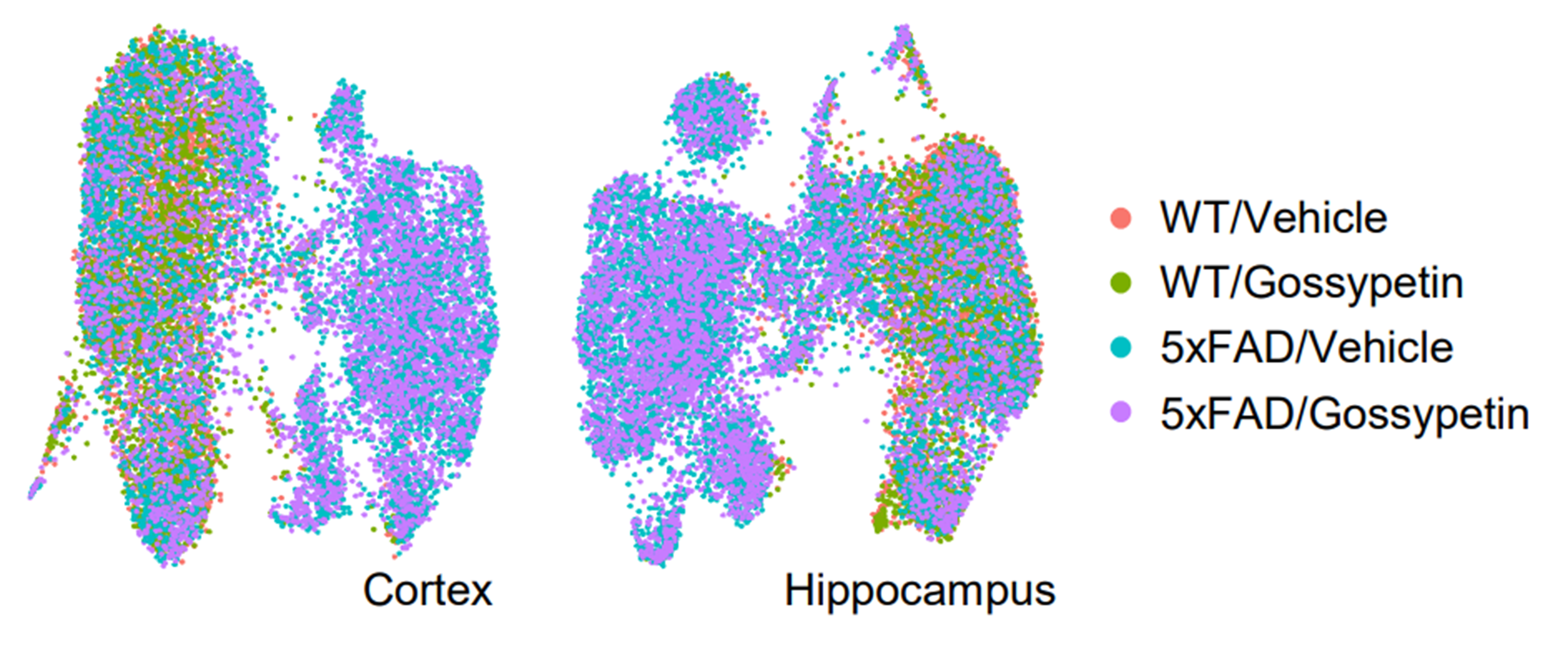

Supplement: Supplementary file 6 — Additional file 6: Fig.S1 Gossypetin does not affect expression of β-, and γ-secretases and activity of β-secretase. (A to G) Time dependent β-secretase activity of mouse hippocampal lysate was measured with Relative Fluorescence Unit (RFU). Fluorescence excitation and emission wavelength was 335 nm and 495 nm respectively (A). Bar graph of RFU at each time point of 10 min (B), 20 min (C), 30 min (D), 40 min (E), 50 min (F), 60 min (G). (n = 10~12 mice per group) (H to L) Representative images of Western blot analysis for β-, γ-secretase subunits, and GAPDH (H). Bar graphs represent relative protein expression levels of BACE1 (I), Nicastrin (J), APH-1 (K), and PEN2 (L). (n = 12~15 mice per group) (M to P) Bar graphs represent relative mRNA expression level of β-, and γ-secretase subunits bace1 (M), ncstn (N), aph1 (O), pen2 (P). (n = 9~10 mice per group) Error bars represent the mean ± SD, *p < 0.05, ns = not significant, two-way ANOVA followed by Tukey’s multiple comparisons test. Fig. S2 Cell type classification of brain samples. (A) UMAP plot showing all cells from the brain samples, colored by their cell types. (B) Heatmap illustrating the Z-scores of average normalized expressions of cell type markers. (C) Violin plots displaying the log-scaled number of detected genes (top), Unique Molecular Identifiers (UMIs) (middle), and the percentage of mitochondrial gene expressions (bottom) per cell for each cell type. (D) UMAP plots showing all cells from the brain samples, colored by their sampled region (left), mouse strain (middle), or drug administration (right) condition. Fig. S3 Detailed subtyping of the microglial population. (A) UMAP plots showing all microglial cells from cortex region. The cells are colored by their celltypes (left). Heatmap showing the Z-scores of average normalized expressions of representative DEGs for each cell type from cortex region (right). (B) UMAP plots showing microglial cells from cortex (left) or hippocampus (right), colored by co [file 13195_2022_1096_MOESM6_ESM.zip › Figure S3B.png]

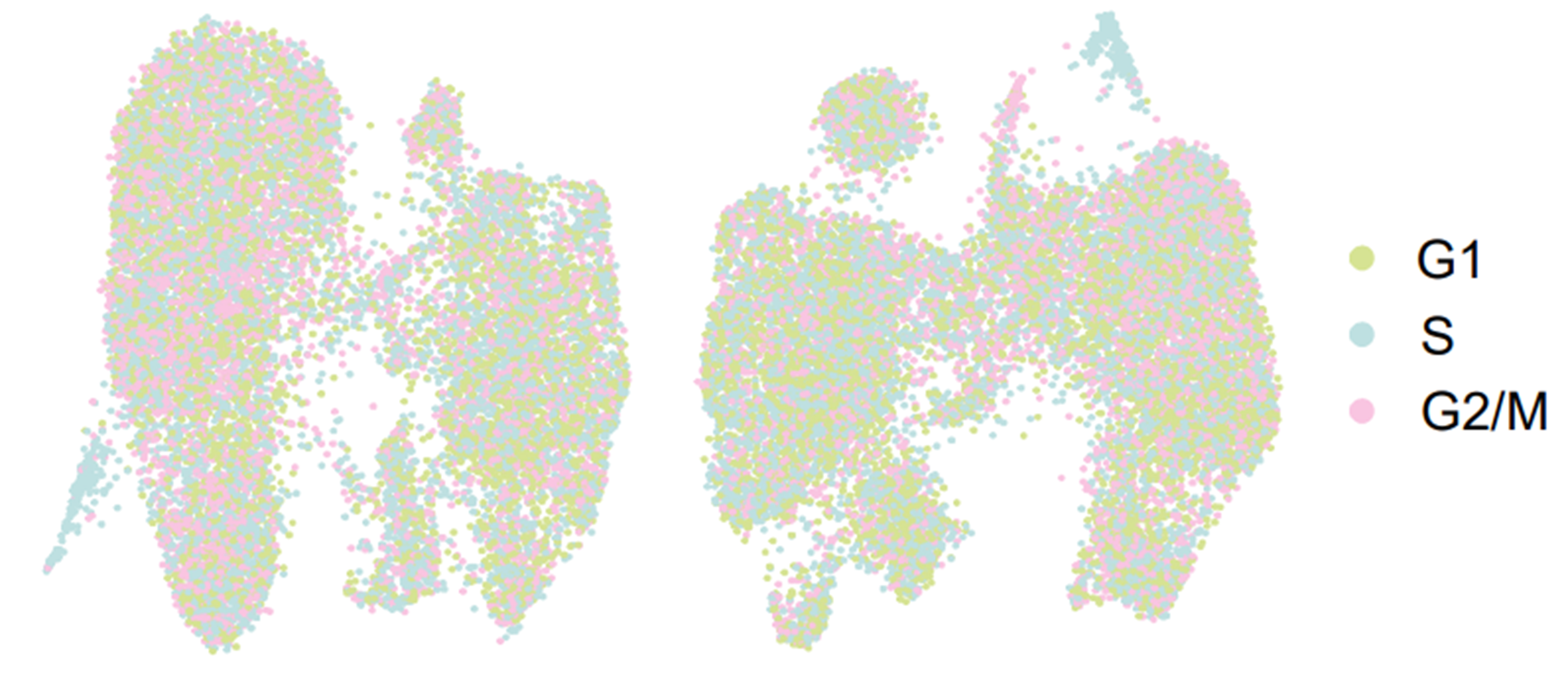

Supplement: Supplementary file 6 — Additional file 6: Fig.S1 Gossypetin does not affect expression of β-, and γ-secretases and activity of β-secretase. (A to G) Time dependent β-secretase activity of mouse hippocampal lysate was measured with Relative Fluorescence Unit (RFU). Fluorescence excitation and emission wavelength was 335 nm and 495 nm respectively (A). Bar graph of RFU at each time point of 10 min (B), 20 min (C), 30 min (D), 40 min (E), 50 min (F), 60 min (G). (n = 10~12 mice per group) (H to L) Representative images of Western blot analysis for β-, γ-secretase subunits, and GAPDH (H). Bar graphs represent relative protein expression levels of BACE1 (I), Nicastrin (J), APH-1 (K), and PEN2 (L). (n = 12~15 mice per group) (M to P) Bar graphs represent relative mRNA expression level of β-, and γ-secretase subunits bace1 (M), ncstn (N), aph1 (O), pen2 (P). (n = 9~10 mice per group) Error bars represent the mean ± SD, *p < 0.05, ns = not significant, two-way ANOVA followed by Tukey’s multiple comparisons test. Fig. S2 Cell type classification of brain samples. (A) UMAP plot showing all cells from the brain samples, colored by their cell types. (B) Heatmap illustrating the Z-scores of average normalized expressions of cell type markers. (C) Violin plots displaying the log-scaled number of detected genes (top), Unique Molecular Identifiers (UMIs) (middle), and the percentage of mitochondrial gene expressions (bottom) per cell for each cell type. (D) UMAP plots showing all cells from the brain samples, colored by their sampled region (left), mouse strain (middle), or drug administration (right) condition. Fig. S3 Detailed subtyping of the microglial population. (A) UMAP plots showing all microglial cells from cortex region. The cells are colored by their celltypes (left). Heatmap showing the Z-scores of average normalized expressions of representative DEGs for each cell type from cortex region (right). (B) UMAP plots showing microglial cells from cortex (left) or hippocampus (right), colored by co [file 13195_2022_1096_MOESM6_ESM.zip › Figure S3C.png]

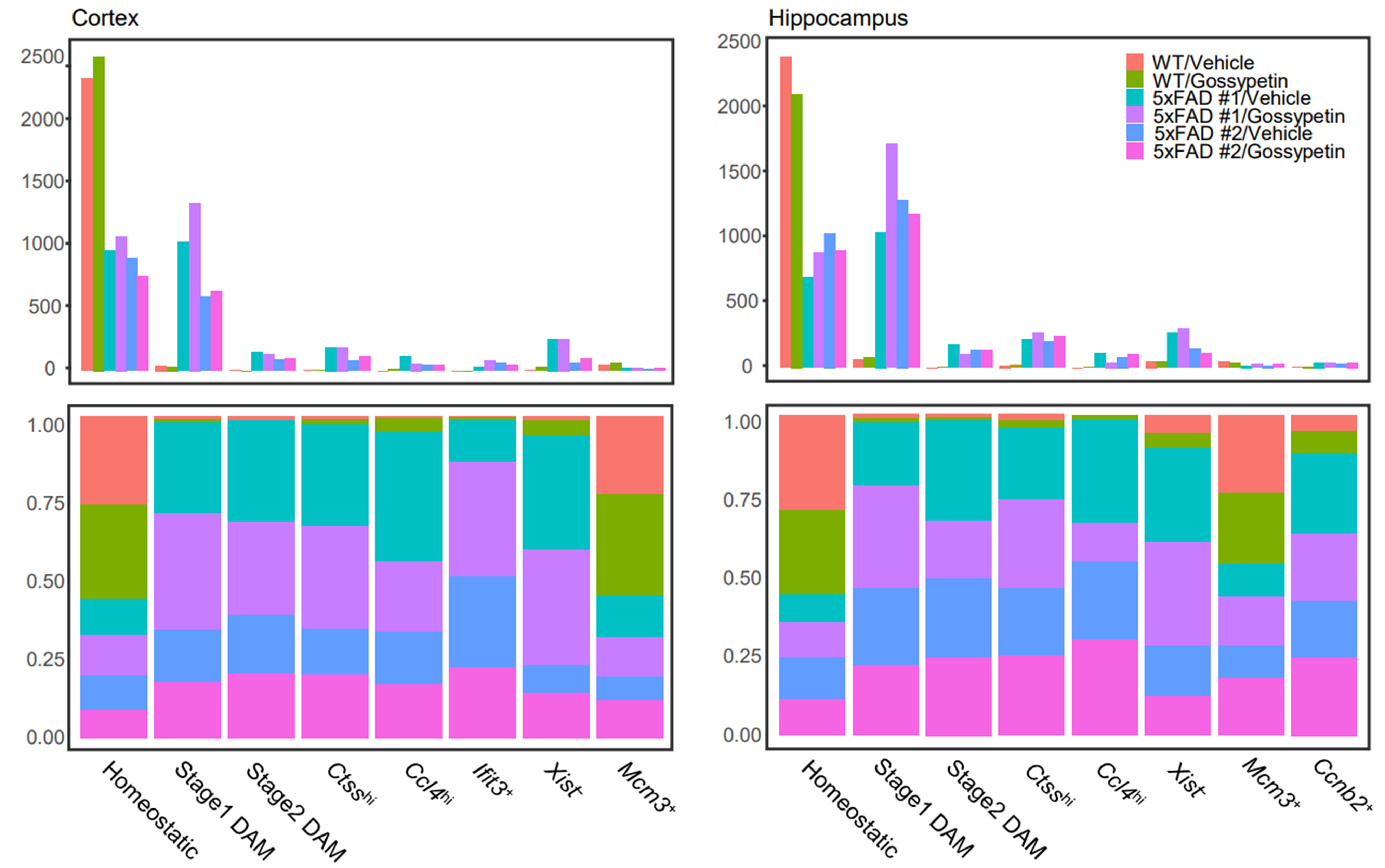

Supplement: Supplementary file 6 — Additional file 6: Fig.S1 Gossypetin does not affect expression of β-, and γ-secretases and activity of β-secretase. (A to G) Time dependent β-secretase activity of mouse hippocampal lysate was measured with Relative Fluorescence Unit (RFU). Fluorescence excitation and emission wavelength was 335 nm and 495 nm respectively (A). Bar graph of RFU at each time point of 10 min (B), 20 min (C), 30 min (D), 40 min (E), 50 min (F), 60 min (G). (n = 10~12 mice per group) (H to L) Representative images of Western blot analysis for β-, γ-secretase subunits, and GAPDH (H). Bar graphs represent relative protein expression levels of BACE1 (I), Nicastrin (J), APH-1 (K), and PEN2 (L). (n = 12~15 mice per group) (M to P) Bar graphs represent relative mRNA expression level of β-, and γ-secretase subunits bace1 (M), ncstn (N), aph1 (O), pen2 (P). (n = 9~10 mice per group) Error bars represent the mean ± SD, *p < 0.05, ns = not significant, two-way ANOVA followed by Tukey’s multiple comparisons test. Fig. S2 Cell type classification of brain samples. (A) UMAP plot showing all cells from the brain samples, colored by their cell types. (B) Heatmap illustrating the Z-scores of average normalized expressions of cell type markers. (C) Violin plots displaying the log-scaled number of detected genes (top), Unique Molecular Identifiers (UMIs) (middle), and the percentage of mitochondrial gene expressions (bottom) per cell for each cell type. (D) UMAP plots showing all cells from the brain samples, colored by their sampled region (left), mouse strain (middle), or drug administration (right) condition. Fig. S3 Detailed subtyping of the microglial population. (A) UMAP plots showing all microglial cells from cortex region. The cells are colored by their celltypes (left). Heatmap showing the Z-scores of average normalized expressions of representative DEGs for each cell type from cortex region (right). (B) UMAP plots showing microglial cells from cortex (left) or hippocampus (right), colored by co [file 13195_2022_1096_MOESM6_ESM.zip › Figure S3D.png]

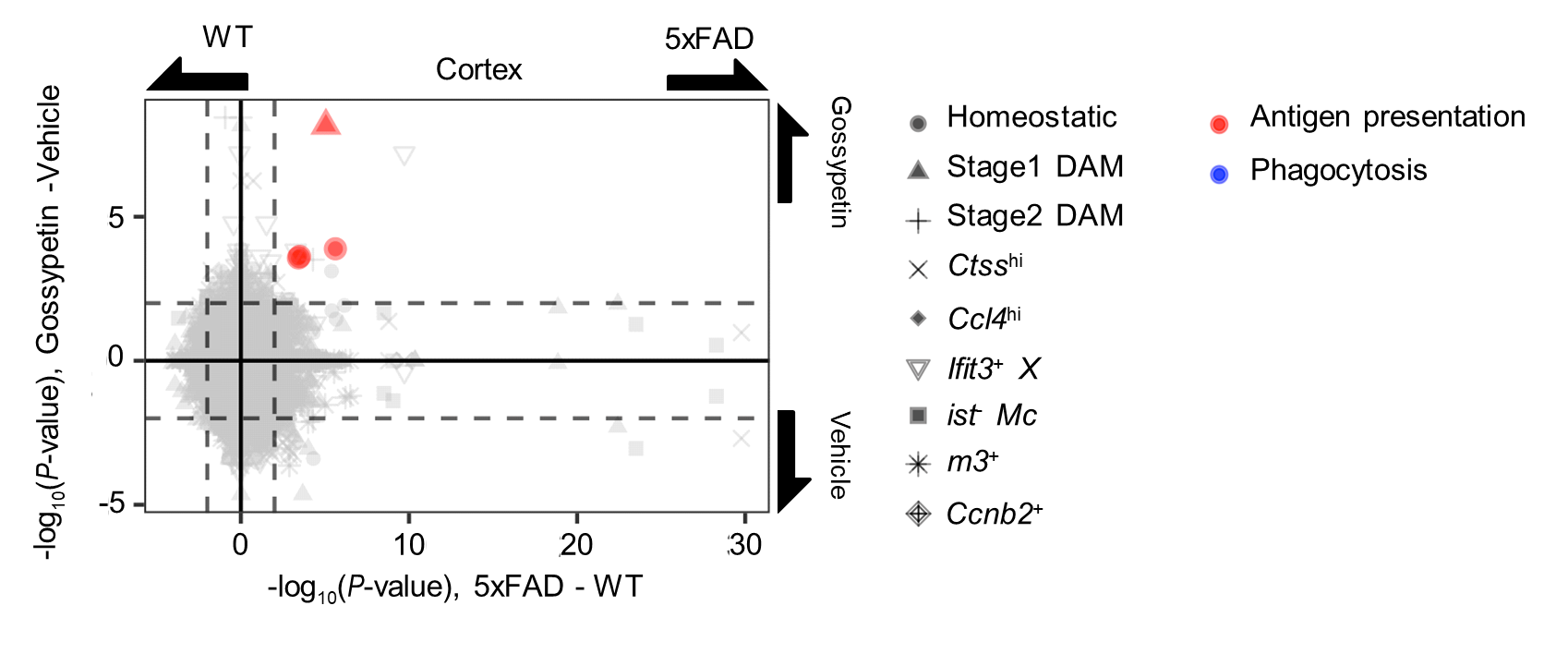

Supplement: Supplementary file 6 — Additional file 6: Fig.S1 Gossypetin does not affect expression of β-, and γ-secretases and activity of β-secretase. (A to G) Time dependent β-secretase activity of mouse hippocampal lysate was measured with Relative Fluorescence Unit (RFU). Fluorescence excitation and emission wavelength was 335 nm and 495 nm respectively (A). Bar graph of RFU at each time point of 10 min (B), 20 min (C), 30 min (D), 40 min (E), 50 min (F), 60 min (G). (n = 10~12 mice per group) (H to L) Representative images of Western blot analysis for β-, γ-secretase subunits, and GAPDH (H). Bar graphs represent relative protein expression levels of BACE1 (I), Nicastrin (J), APH-1 (K), and PEN2 (L). (n = 12~15 mice per group) (M to P) Bar graphs represent relative mRNA expression level of β-, and γ-secretase subunits bace1 (M), ncstn (N), aph1 (O), pen2 (P). (n = 9~10 mice per group) Error bars represent the mean ± SD, *p < 0.05, ns = not significant, two-way ANOVA followed by Tukey’s multiple comparisons test. Fig. S2 Cell type classification of brain samples. (A) UMAP plot showing all cells from the brain samples, colored by their cell types. (B) Heatmap illustrating the Z-scores of average normalized expressions of cell type markers. (C) Violin plots displaying the log-scaled number of detected genes (top), Unique Molecular Identifiers (UMIs) (middle), and the percentage of mitochondrial gene expressions (bottom) per cell for each cell type. (D) UMAP plots showing all cells from the brain samples, colored by their sampled region (left), mouse strain (middle), or drug administration (right) condition. Fig. S3 Detailed subtyping of the microglial population. (A) UMAP plots showing all microglial cells from cortex region. The cells are colored by their celltypes (left). Heatmap showing the Z-scores of average normalized expressions of representative DEGs for each cell type from cortex region (right). (B) UMAP plots showing microglial cells from cortex (left) or hippocampus (right), colored by co [file 13195_2022_1096_MOESM6_ESM.zip › Figure S4A.png]

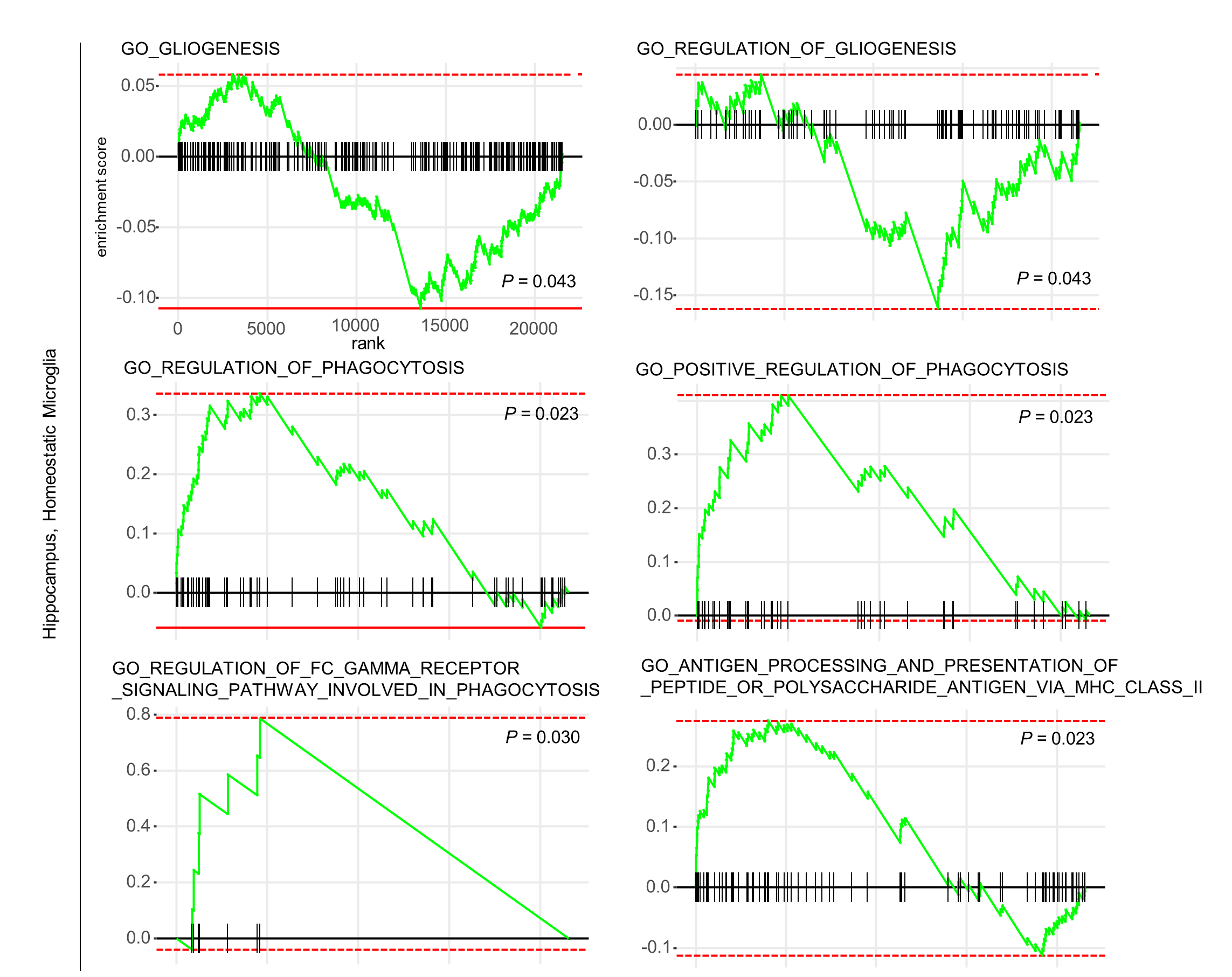

Supplement: Supplementary file 6 — Additional file 6: Fig.S1 Gossypetin does not affect expression of β-, and γ-secretases and activity of β-secretase. (A to G) Time dependent β-secretase activity of mouse hippocampal lysate was measured with Relative Fluorescence Unit (RFU). Fluorescence excitation and emission wavelength was 335 nm and 495 nm respectively (A). Bar graph of RFU at each time point of 10 min (B), 20 min (C), 30 min (D), 40 min (E), 50 min (F), 60 min (G). (n = 10~12 mice per group) (H to L) Representative images of Western blot analysis for β-, γ-secretase subunits, and GAPDH (H). Bar graphs represent relative protein expression levels of BACE1 (I), Nicastrin (J), APH-1 (K), and PEN2 (L). (n = 12~15 mice per group) (M to P) Bar graphs represent relative mRNA expression level of β-, and γ-secretase subunits bace1 (M), ncstn (N), aph1 (O), pen2 (P). (n = 9~10 mice per group) Error bars represent the mean ± SD, *p < 0.05, ns = not significant, two-way ANOVA followed by Tukey’s multiple comparisons test. Fig. S2 Cell type classification of brain samples. (A) UMAP plot showing all cells from the brain samples, colored by their cell types. (B) Heatmap illustrating the Z-scores of average normalized expressions of cell type markers. (C) Violin plots displaying the log-scaled number of detected genes (top), Unique Molecular Identifiers (UMIs) (middle), and the percentage of mitochondrial gene expressions (bottom) per cell for each cell type. (D) UMAP plots showing all cells from the brain samples, colored by their sampled region (left), mouse strain (middle), or drug administration (right) condition. Fig. S3 Detailed subtyping of the microglial population. (A) UMAP plots showing all microglial cells from cortex region. The cells are colored by their celltypes (left). Heatmap showing the Z-scores of average normalized expressions of representative DEGs for each cell type from cortex region (right). (B) UMAP plots showing microglial cells from cortex (left) or hippocampus (right), colored by co [file 13195_2022_1096_MOESM6_ESM.zip › Figure S4B.png]

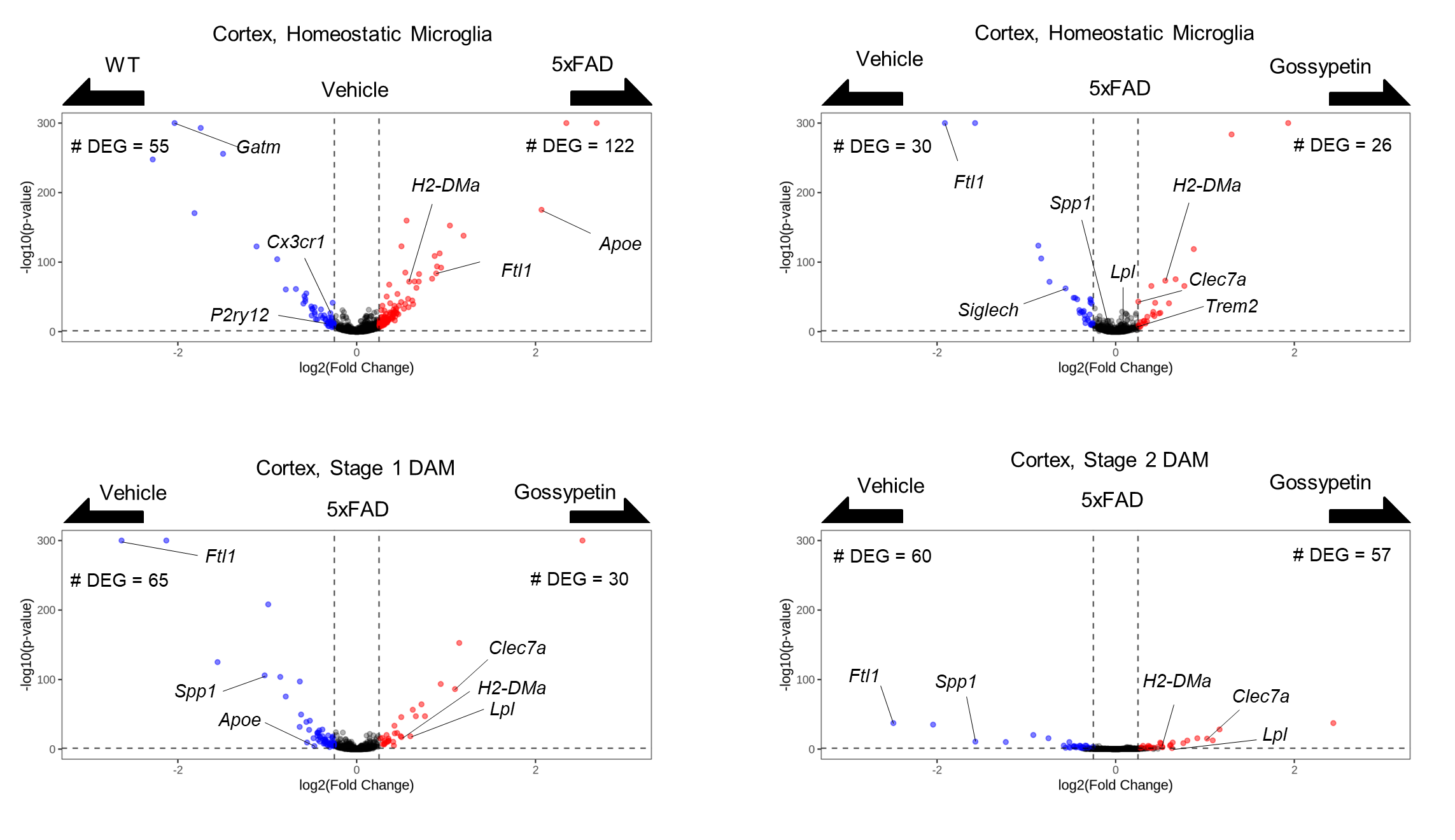

Supplement: Supplementary file 6 — Additional file 6: Fig.S1 Gossypetin does not affect expression of β-, and γ-secretases and activity of β-secretase. (A to G) Time dependent β-secretase activity of mouse hippocampal lysate was measured with Relative Fluorescence Unit (RFU). Fluorescence excitation and emission wavelength was 335 nm and 495 nm respectively (A). Bar graph of RFU at each time point of 10 min (B), 20 min (C), 30 min (D), 40 min (E), 50 min (F), 60 min (G). (n = 10~12 mice per group) (H to L) Representative images of Western blot analysis for β-, γ-secretase subunits, and GAPDH (H). Bar graphs represent relative protein expression levels of BACE1 (I), Nicastrin (J), APH-1 (K), and PEN2 (L). (n = 12~15 mice per group) (M to P) Bar graphs represent relative mRNA expression level of β-, and γ-secretase subunits bace1 (M), ncstn (N), aph1 (O), pen2 (P). (n = 9~10 mice per group) Error bars represent the mean ± SD, *p < 0.05, ns = not significant, two-way ANOVA followed by Tukey’s multiple comparisons test. Fig. S2 Cell type classification of brain samples. (A) UMAP plot showing all cells from the brain samples, colored by their cell types. (B) Heatmap illustrating the Z-scores of average normalized expressions of cell type markers. (C) Violin plots displaying the log-scaled number of detected genes (top), Unique Molecular Identifiers (UMIs) (middle), and the percentage of mitochondrial gene expressions (bottom) per cell for each cell type. (D) UMAP plots showing all cells from the brain samples, colored by their sampled region (left), mouse strain (middle), or drug administration (right) condition. Fig. S3 Detailed subtyping of the microglial population. (A) UMAP plots showing all microglial cells from cortex region. The cells are colored by their celltypes (left). Heatmap showing the Z-scores of average normalized expressions of representative DEGs for each cell type from cortex region (right). (B) UMAP plots showing microglial cells from cortex (left) or hippocampus (right), colored by co [file 13195_2022_1096_MOESM6_ESM.zip › Figure S5A.png]

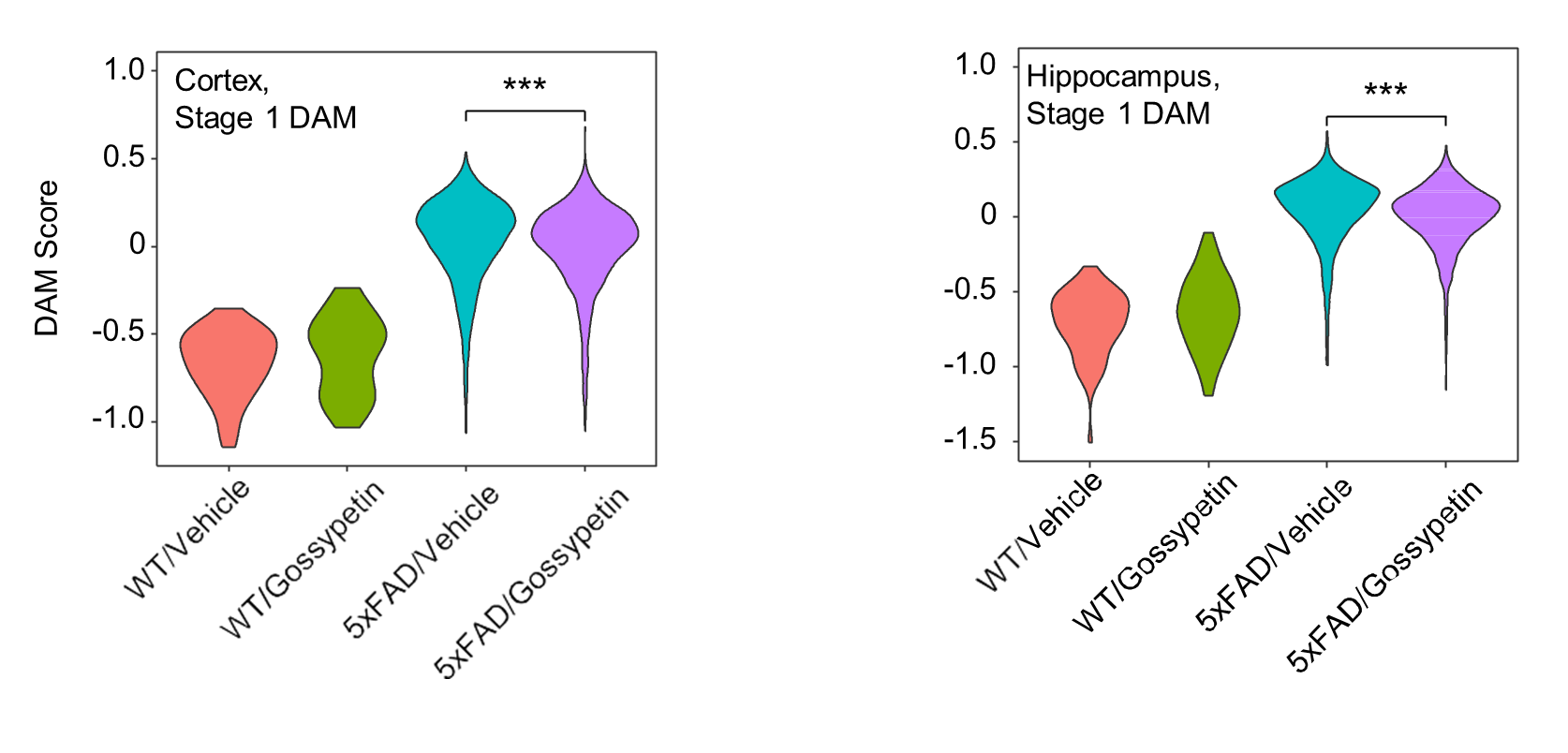

Supplement: Supplementary file 6 — Additional file 6: Fig.S1 Gossypetin does not affect expression of β-, and γ-secretases and activity of β-secretase. (A to G) Time dependent β-secretase activity of mouse hippocampal lysate was measured with Relative Fluorescence Unit (RFU). Fluorescence excitation and emission wavelength was 335 nm and 495 nm respectively (A). Bar graph of RFU at each time point of 10 min (B), 20 min (C), 30 min (D), 40 min (E), 50 min (F), 60 min (G). (n = 10~12 mice per group) (H to L) Representative images of Western blot analysis for β-, γ-secretase subunits, and GAPDH (H). Bar graphs represent relative protein expression levels of BACE1 (I), Nicastrin (J), APH-1 (K), and PEN2 (L). (n = 12~15 mice per group) (M to P) Bar graphs represent relative mRNA expression level of β-, and γ-secretase subunits bace1 (M), ncstn (N), aph1 (O), pen2 (P). (n = 9~10 mice per group) Error bars represent the mean ± SD, *p < 0.05, ns = not significant, two-way ANOVA followed by Tukey’s multiple comparisons test. Fig. S2 Cell type classification of brain samples. (A) UMAP plot showing all cells from the brain samples, colored by their cell types. (B) Heatmap illustrating the Z-scores of average normalized expressions of cell type markers. (C) Violin plots displaying the log-scaled number of detected genes (top), Unique Molecular Identifiers (UMIs) (middle), and the percentage of mitochondrial gene expressions (bottom) per cell for each cell type. (D) UMAP plots showing all cells from the brain samples, colored by their sampled region (left), mouse strain (middle), or drug administration (right) condition. Fig. S3 Detailed subtyping of the microglial population. (A) UMAP plots showing all microglial cells from cortex region. The cells are colored by their celltypes (left). Heatmap showing the Z-scores of average normalized expressions of representative DEGs for each cell type from cortex region (right). (B) UMAP plots showing microglial cells from cortex (left) or hippocampus (right), colored by co [file 13195_2022_1096_MOESM6_ESM.zip › Figure S5B.png]

Figure S6. Gossypetin ameliorates gliosis in microglia and astrocytes

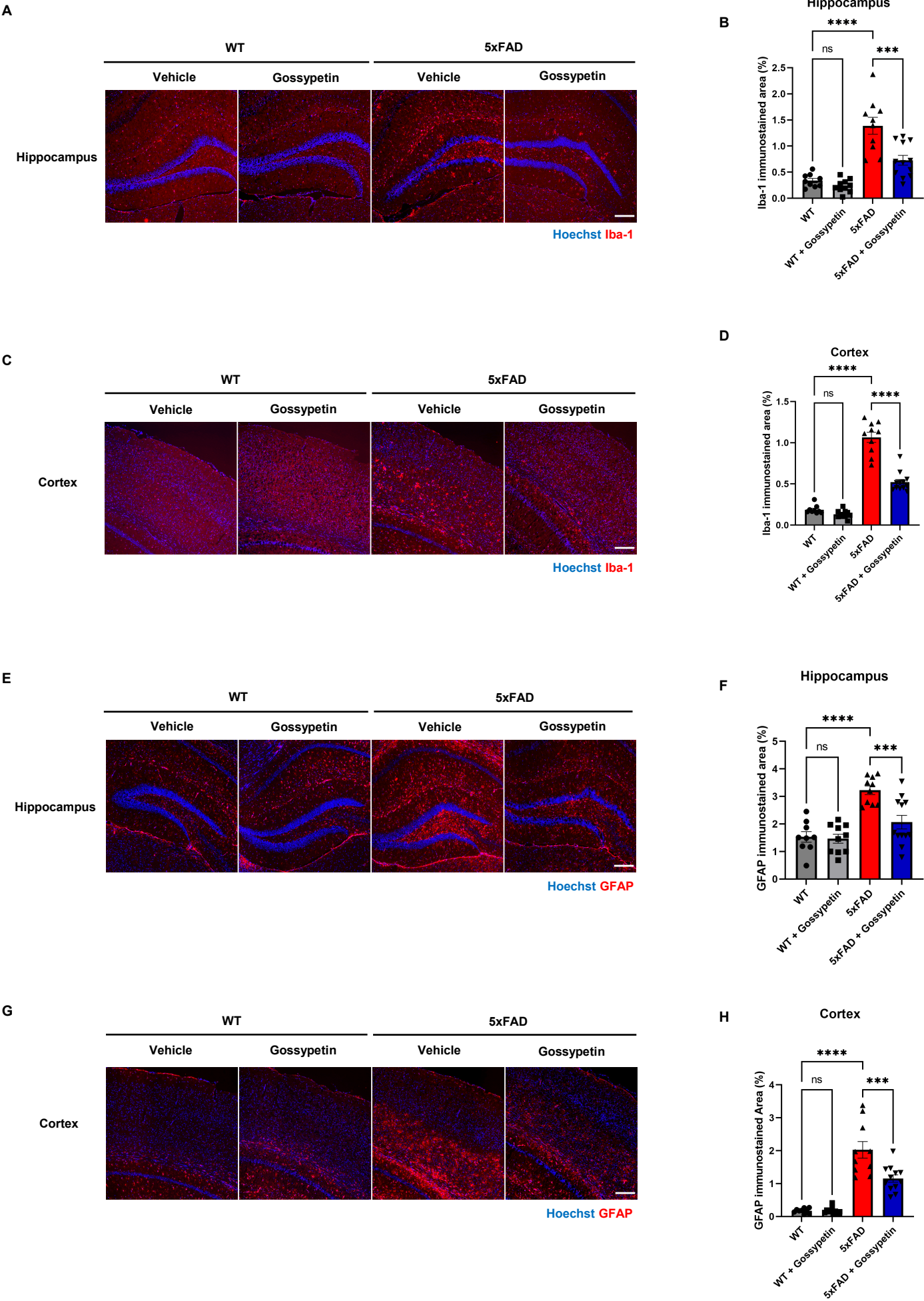

Supplement: Supplementary file 6 — Additional file 6: Fig.S1 Gossypetin does not affect expression of β-, and γ-secretases and activity of β-secretase. (A to G) Time dependent β-secretase activity of mouse hippocampal lysate was measured with Relative Fluorescence Unit (RFU). Fluorescence excitation and emission wavelength was 335 nm and 495 nm respectively (A). Bar graph of RFU at each time point of 10 min (B), 20 min (C), 30 min (D), 40 min (E), 50 min (F), 60 min (G). (n = 10~12 mice per group) (H to L) Representative images of Western blot analysis for β-, γ-secretase subunits, and GAPDH (H). Bar graphs represent relative protein expression levels of BACE1 (I), Nicastrin (J), APH-1 (K), and PEN2 (L). (n = 12~15 mice per group) (M to P) Bar graphs represent relative mRNA expression level of β-, and γ-secretase subunits bace1 (M), ncstn (N), aph1 (O), pen2 (P). (n = 9~10 mice per group) Error bars represent the mean ± SD, *p < 0.05, ns = not significant, two-way ANOVA followed by Tukey’s multiple comparisons test. Fig. S2 Cell type classification of brain samples. (A) UMAP plot showing all cells from the brain samples, colored by their cell types. (B) Heatmap illustrating the Z-scores of average normalized expressions of cell type markers. (C) Violin plots displaying the log-scaled number of detected genes (top), Unique Molecular Identifiers (UMIs) (middle), and the percentage of mitochondrial gene expressions (bottom) per cell for each cell type. (D) UMAP plots showing all cells from the brain samples, colored by their sampled region (left), mouse strain (middle), or drug administration (right) condition. Fig. S3 Detailed subtyping of the microglial population. (A) UMAP plots showing all microglial cells from cortex region. The cells are colored by their celltypes (left). Heatmap showing the Z-scores of average normalized expressions of representative DEGs for each cell type from cortex region (right). (B) UMAP plots showing microglial cells from cortex (left) or hippocampus (right), colored by co [file 13195_2022_1096_MOESM6_ESM.zip › Figure S6 (Total).pdf]

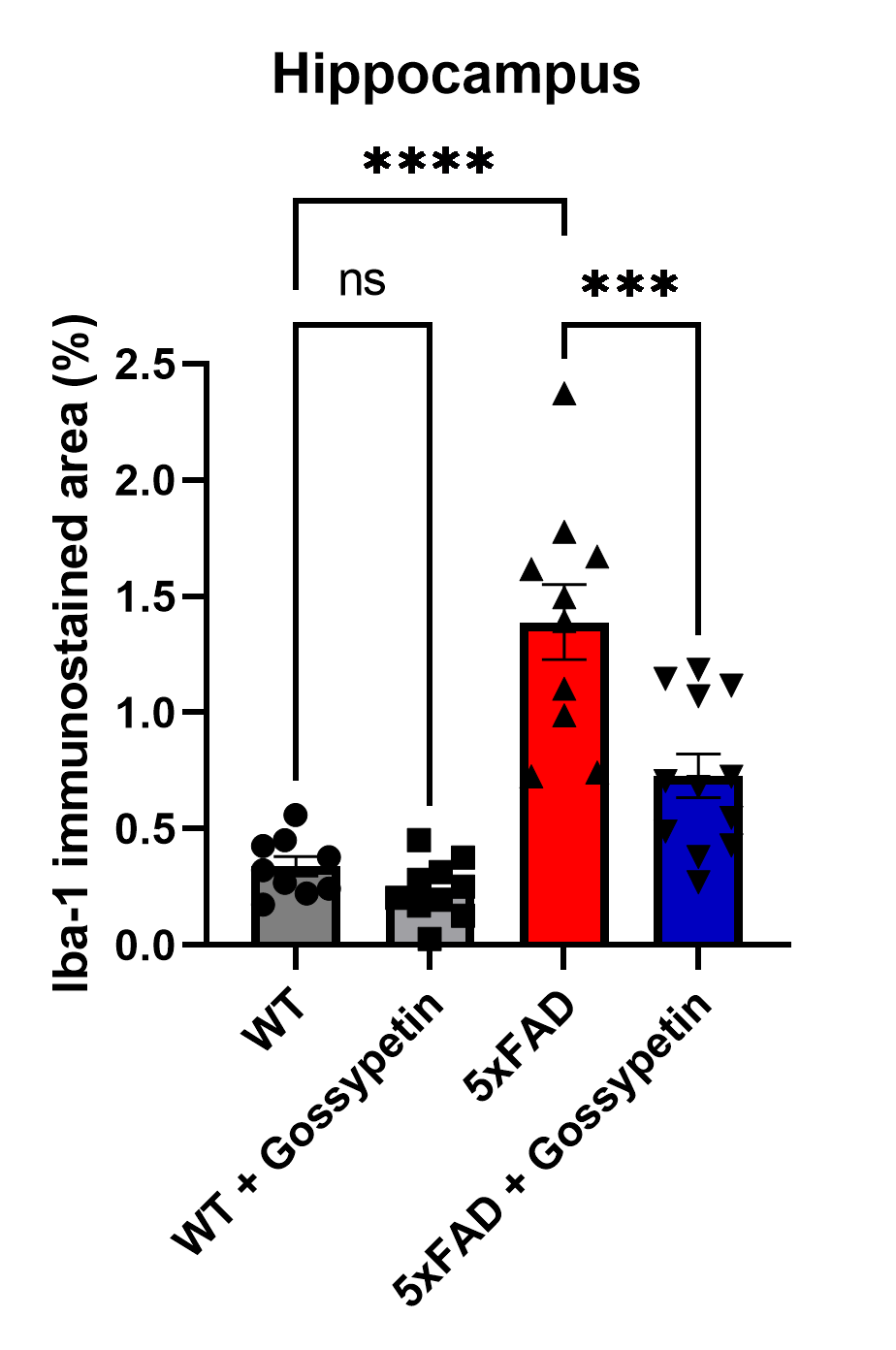

Supplement: Supplementary file 6 — Additional file 6: Fig.S1 Gossypetin does not affect expression of β-, and γ-secretases and activity of β-secretase. (A to G) Time dependent β-secretase activity of mouse hippocampal lysate was measured with Relative Fluorescence Unit (RFU). Fluorescence excitation and emission wavelength was 335 nm and 495 nm respectively (A). Bar graph of RFU at each time point of 10 min (B), 20 min (C), 30 min (D), 40 min (E), 50 min (F), 60 min (G). (n = 10~12 mice per group) (H to L) Representative images of Western blot analysis for β-, γ-secretase subunits, and GAPDH (H). Bar graphs represent relative protein expression levels of BACE1 (I), Nicastrin (J), APH-1 (K), and PEN2 (L). (n = 12~15 mice per group) (M to P) Bar graphs represent relative mRNA expression level of β-, and γ-secretase subunits bace1 (M), ncstn (N), aph1 (O), pen2 (P). (n = 9~10 mice per group) Error bars represent the mean ± SD, *p < 0.05, ns = not significant, two-way ANOVA followed by Tukey’s multiple comparisons test. Fig. S2 Cell type classification of brain samples. (A) UMAP plot showing all cells from the brain samples, colored by their cell types. (B) Heatmap illustrating the Z-scores of average normalized expressions of cell type markers. (C) Violin plots displaying the log-scaled number of detected genes (top), Unique Molecular Identifiers (UMIs) (middle), and the percentage of mitochondrial gene expressions (bottom) per cell for each cell type. (D) UMAP plots showing all cells from the brain samples, colored by their sampled region (left), mouse strain (middle), or drug administration (right) condition. Fig. S3 Detailed subtyping of the microglial population. (A) UMAP plots showing all microglial cells from cortex region. The cells are colored by their celltypes (left). Heatmap showing the Z-scores of average normalized expressions of representative DEGs for each cell type from cortex region (right). (B) UMAP plots showing microglial cells from cortex (left) or hippocampus (right), colored by co [file 13195_2022_1096_MOESM6_ESM.zip › Figure S6B.png]

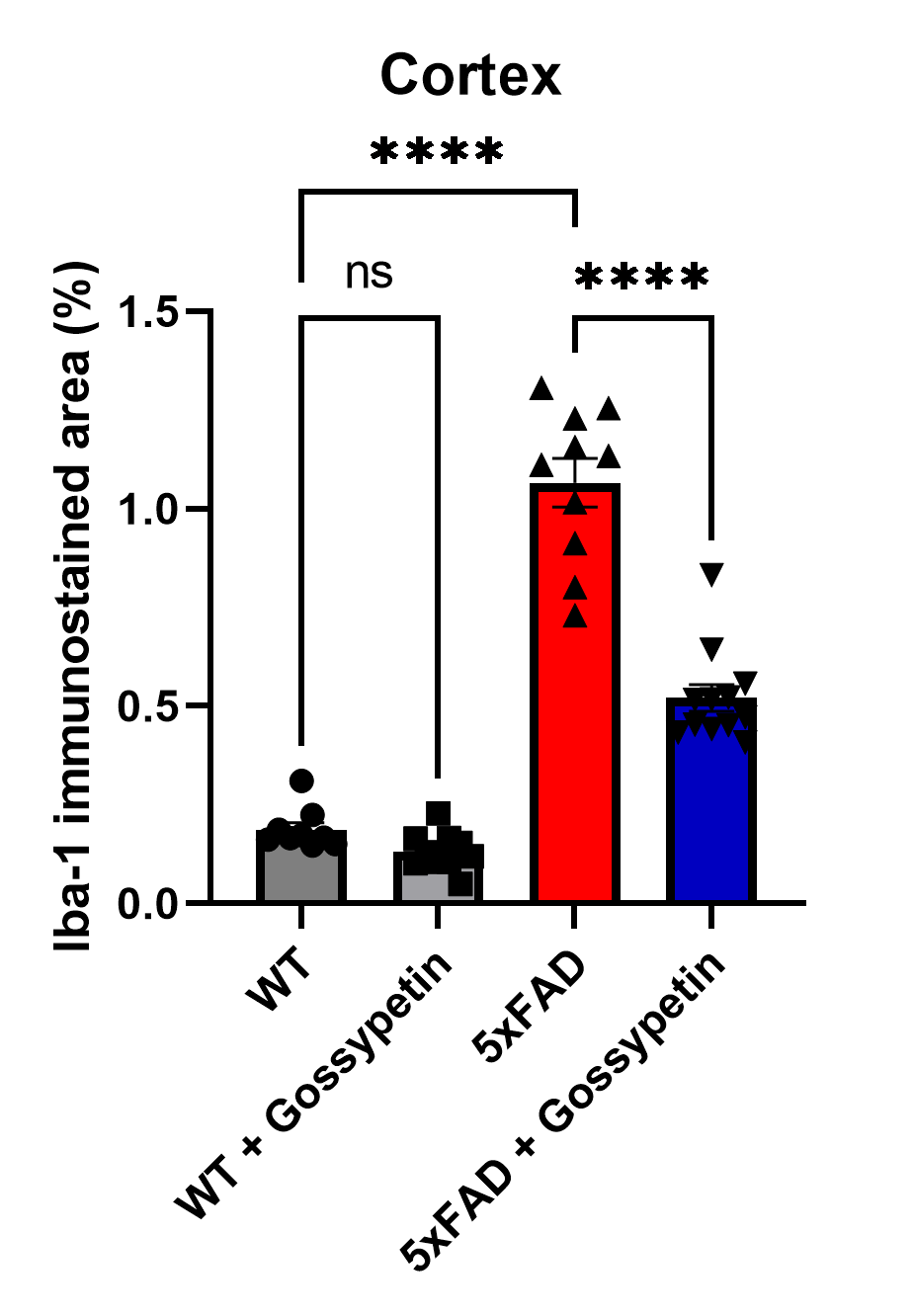

Supplement: Supplementary file 6 — Additional file 6: Fig.S1 Gossypetin does not affect expression of β-, and γ-secretases and activity of β-secretase. (A to G) Time dependent β-secretase activity of mouse hippocampal lysate was measured with Relative Fluorescence Unit (RFU). Fluorescence excitation and emission wavelength was 335 nm and 495 nm respectively (A). Bar graph of RFU at each time point of 10 min (B), 20 min (C), 30 min (D), 40 min (E), 50 min (F), 60 min (G). (n = 10~12 mice per group) (H to L) Representative images of Western blot analysis for β-, γ-secretase subunits, and GAPDH (H). Bar graphs represent relative protein expression levels of BACE1 (I), Nicastrin (J), APH-1 (K), and PEN2 (L). (n = 12~15 mice per group) (M to P) Bar graphs represent relative mRNA expression level of β-, and γ-secretase subunits bace1 (M), ncstn (N), aph1 (O), pen2 (P). (n = 9~10 mice per group) Error bars represent the mean ± SD, *p < 0.05, ns = not significant, two-way ANOVA followed by Tukey’s multiple comparisons test. Fig. S2 Cell type classification of brain samples. (A) UMAP plot showing all cells from the brain samples, colored by their cell types. (B) Heatmap illustrating the Z-scores of average normalized expressions of cell type markers. (C) Violin plots displaying the log-scaled number of detected genes (top), Unique Molecular Identifiers (UMIs) (middle), and the percentage of mitochondrial gene expressions (bottom) per cell for each cell type. (D) UMAP plots showing all cells from the brain samples, colored by their sampled region (left), mouse strain (middle), or drug administration (right) condition. Fig. S3 Detailed subtyping of the microglial population. (A) UMAP plots showing all microglial cells from cortex region. The cells are colored by their celltypes (left). Heatmap showing the Z-scores of average normalized expressions of representative DEGs for each cell type from cortex region (right). (B) UMAP plots showing microglial cells from cortex (left) or hippocampus (right), colored by co [file 13195_2022_1096_MOESM6_ESM.zip › Figure S6D.png]

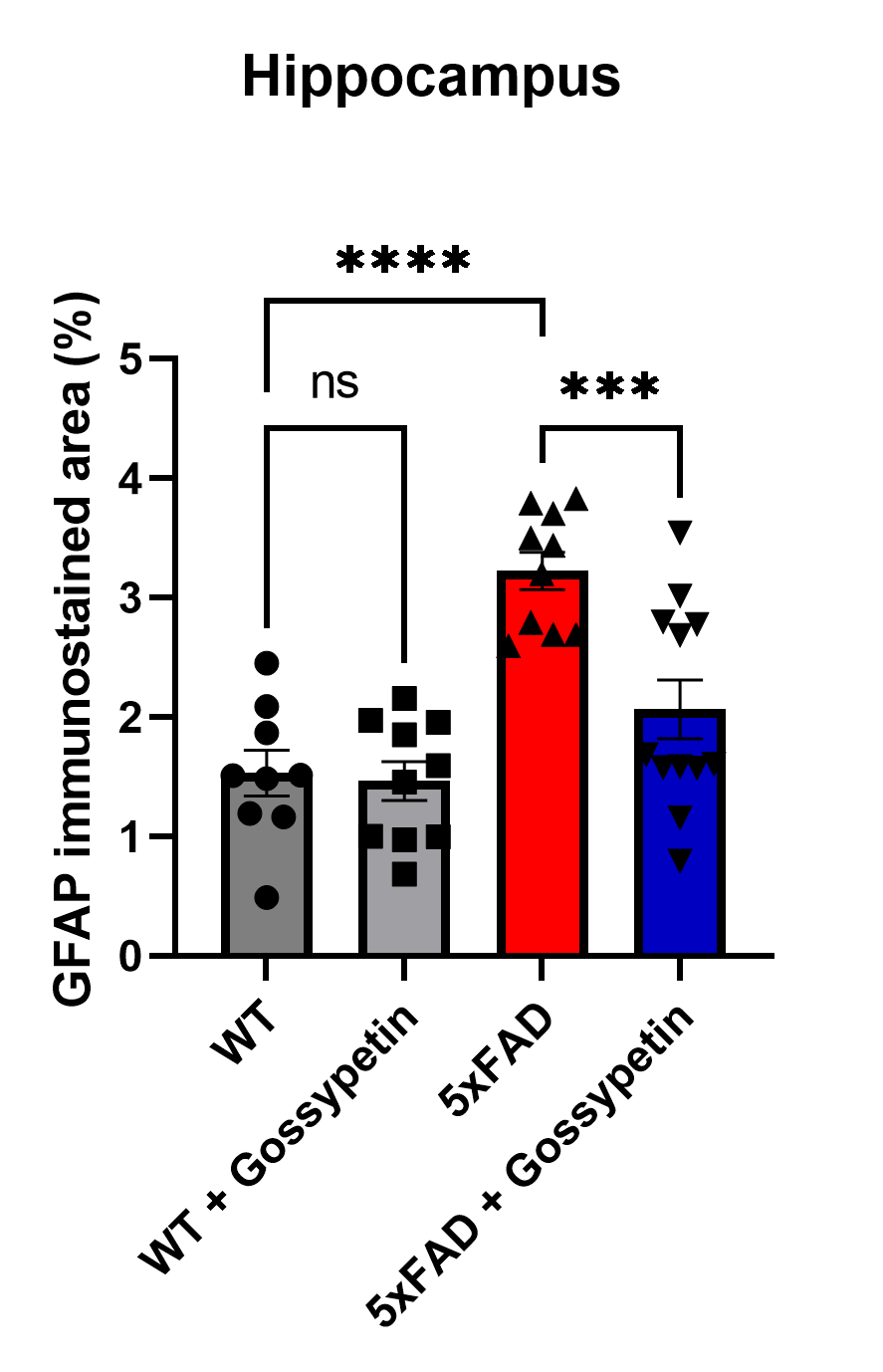

Supplement: Supplementary file 6 — Additional file 6: Fig.S1 Gossypetin does not affect expression of β-, and γ-secretases and activity of β-secretase. (A to G) Time dependent β-secretase activity of mouse hippocampal lysate was measured with Relative Fluorescence Unit (RFU). Fluorescence excitation and emission wavelength was 335 nm and 495 nm respectively (A). Bar graph of RFU at each time point of 10 min (B), 20 min (C), 30 min (D), 40 min (E), 50 min (F), 60 min (G). (n = 10~12 mice per group) (H to L) Representative images of Western blot analysis for β-, γ-secretase subunits, and GAPDH (H). Bar graphs represent relative protein expression levels of BACE1 (I), Nicastrin (J), APH-1 (K), and PEN2 (L). (n = 12~15 mice per group) (M to P) Bar graphs represent relative mRNA expression level of β-, and γ-secretase subunits bace1 (M), ncstn (N), aph1 (O), pen2 (P). (n = 9~10 mice per group) Error bars represent the mean ± SD, *p < 0.05, ns = not significant, two-way ANOVA followed by Tukey’s multiple comparisons test. Fig. S2 Cell type classification of brain samples. (A) UMAP plot showing all cells from the brain samples, colored by their cell types. (B) Heatmap illustrating the Z-scores of average normalized expressions of cell type markers. (C) Violin plots displaying the log-scaled number of detected genes (top), Unique Molecular Identifiers (UMIs) (middle), and the percentage of mitochondrial gene expressions (bottom) per cell for each cell type. (D) UMAP plots showing all cells from the brain samples, colored by their sampled region (left), mouse strain (middle), or drug administration (right) condition. Fig. S3 Detailed subtyping of the microglial population. (A) UMAP plots showing all microglial cells from cortex region. The cells are colored by their celltypes (left). Heatmap showing the Z-scores of average normalized expressions of representative DEGs for each cell type from cortex region (right). (B) UMAP plots showing microglial cells from cortex (left) or hippocampus (right), colored by co [file 13195_2022_1096_MOESM6_ESM.zip › Figure S6F.png]

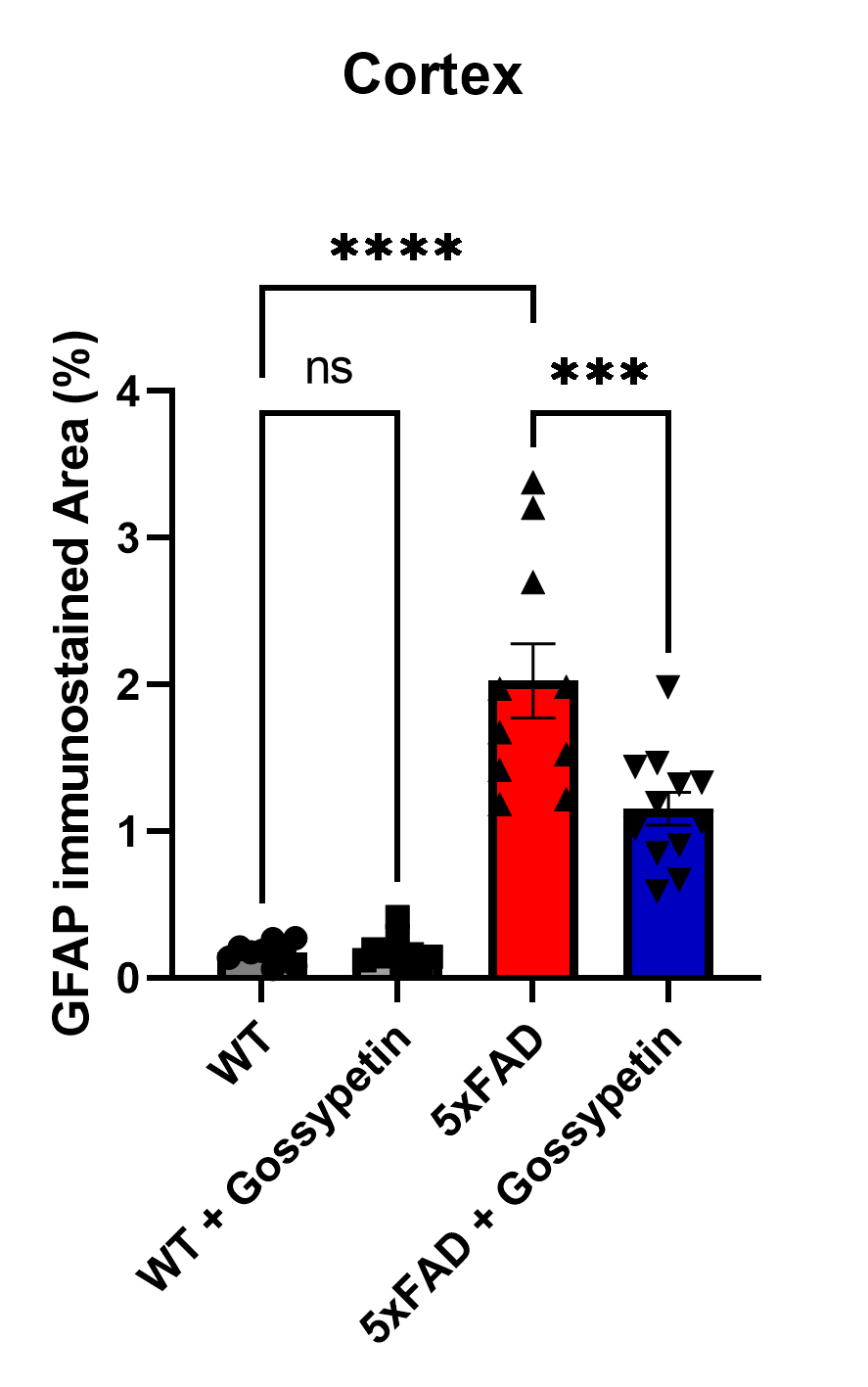

Supplement: Supplementary file 6 — Additional file 6: Fig.S1 Gossypetin does not affect expression of β-, and γ-secretases and activity of β-secretase. (A to G) Time dependent β-secretase activity of mouse hippocampal lysate was measured with Relative Fluorescence Unit (RFU). Fluorescence excitation and emission wavelength was 335 nm and 495 nm respectively (A). Bar graph of RFU at each time point of 10 min (B), 20 min (C), 30 min (D), 40 min (E), 50 min (F), 60 min (G). (n = 10~12 mice per group) (H to L) Representative images of Western blot analysis for β-, γ-secretase subunits, and GAPDH (H). Bar graphs represent relative protein expression levels of BACE1 (I), Nicastrin (J), APH-1 (K), and PEN2 (L). (n = 12~15 mice per group) (M to P) Bar graphs represent relative mRNA expression level of β-, and γ-secretase subunits bace1 (M), ncstn (N), aph1 (O), pen2 (P). (n = 9~10 mice per group) Error bars represent the mean ± SD, *p < 0.05, ns = not significant, two-way ANOVA followed by Tukey’s multiple comparisons test. Fig. S2 Cell type classification of brain samples. (A) UMAP plot showing all cells from the brain samples, colored by their cell types. (B) Heatmap illustrating the Z-scores of average normalized expressions of cell type markers. (C) Violin plots displaying the log-scaled number of detected genes (top), Unique Molecular Identifiers (UMIs) (middle), and the percentage of mitochondrial gene expressions (bottom) per cell for each cell type. (D) UMAP plots showing all cells from the brain samples, colored by their sampled region (left), mouse strain (middle), or drug administration (right) condition. Fig. S3 Detailed subtyping of the microglial population. (A) UMAP plots showing all microglial cells from cortex region. The cells are colored by their celltypes (left). Heatmap showing the Z-scores of average normalized expressions of representative DEGs for each cell type from cortex region (right). (B) UMAP plots showing microglial cells from cortex (left) or hippocampus (right), colored by co [file 13195_2022_1096_MOESM6_ESM.zip › Figure S6H.png]

Figure S7. Gossypetin increases Aβ phagocytic capacity and dynamics of BV2 microglial cell line

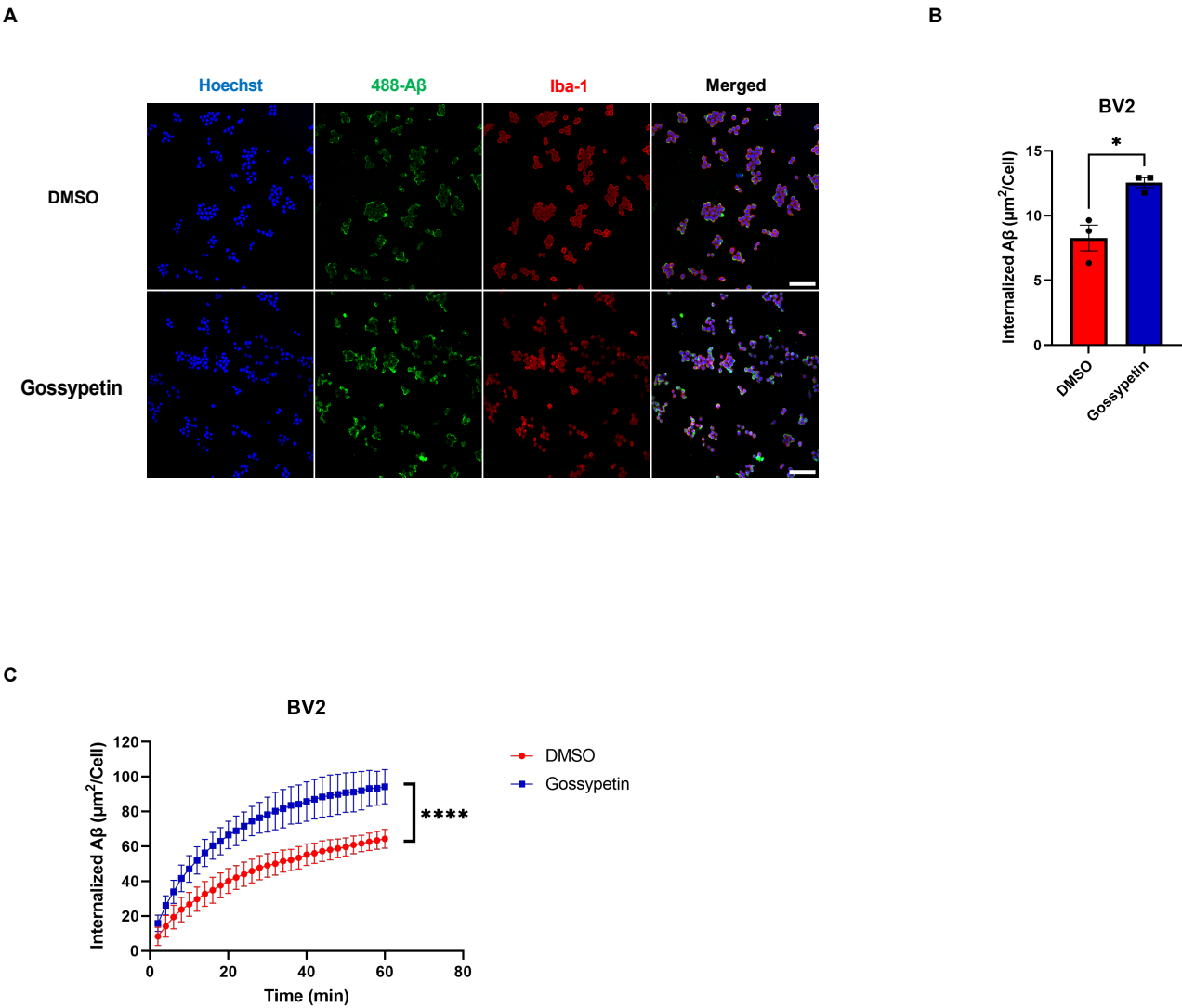

Supplement: Supplementary file 6 — Additional file 6: Fig.S1 Gossypetin does not affect expression of β-, and γ-secretases and activity of β-secretase. (A to G) Time dependent β-secretase activity of mouse hippocampal lysate was measured with Relative Fluorescence Unit (RFU). Fluorescence excitation and emission wavelength was 335 nm and 495 nm respectively (A). Bar graph of RFU at each time point of 10 min (B), 20 min (C), 30 min (D), 40 min (E), 50 min (F), 60 min (G). (n = 10~12 mice per group) (H to L) Representative images of Western blot analysis for β-, γ-secretase subunits, and GAPDH (H). Bar graphs represent relative protein expression levels of BACE1 (I), Nicastrin (J), APH-1 (K), and PEN2 (L). (n = 12~15 mice per group) (M to P) Bar graphs represent relative mRNA expression level of β-, and γ-secretase subunits bace1 (M), ncstn (N), aph1 (O), pen2 (P). (n = 9~10 mice per group) Error bars represent the mean ± SD, *p < 0.05, ns = not significant, two-way ANOVA followed by Tukey’s multiple comparisons test. Fig. S2 Cell type classification of brain samples. (A) UMAP plot showing all cells from the brain samples, colored by their cell types. (B) Heatmap illustrating the Z-scores of average normalized expressions of cell type markers. (C) Violin plots displaying the log-scaled number of detected genes (top), Unique Molecular Identifiers (UMIs) (middle), and the percentage of mitochondrial gene expressions (bottom) per cell for each cell type. (D) UMAP plots showing all cells from the brain samples, colored by their sampled region (left), mouse strain (middle), or drug administration (right) condition. Fig. S3 Detailed subtyping of the microglial population. (A) UMAP plots showing all microglial cells from cortex region. The cells are colored by their celltypes (left). Heatmap showing the Z-scores of average normalized expressions of representative DEGs for each cell type from cortex region (right). (B) UMAP plots showing microglial cells from cortex (left) or hippocampus (right), colored by co [file 13195_2022_1096_MOESM6_ESM.zip › Figure S7 (Total).pdf]

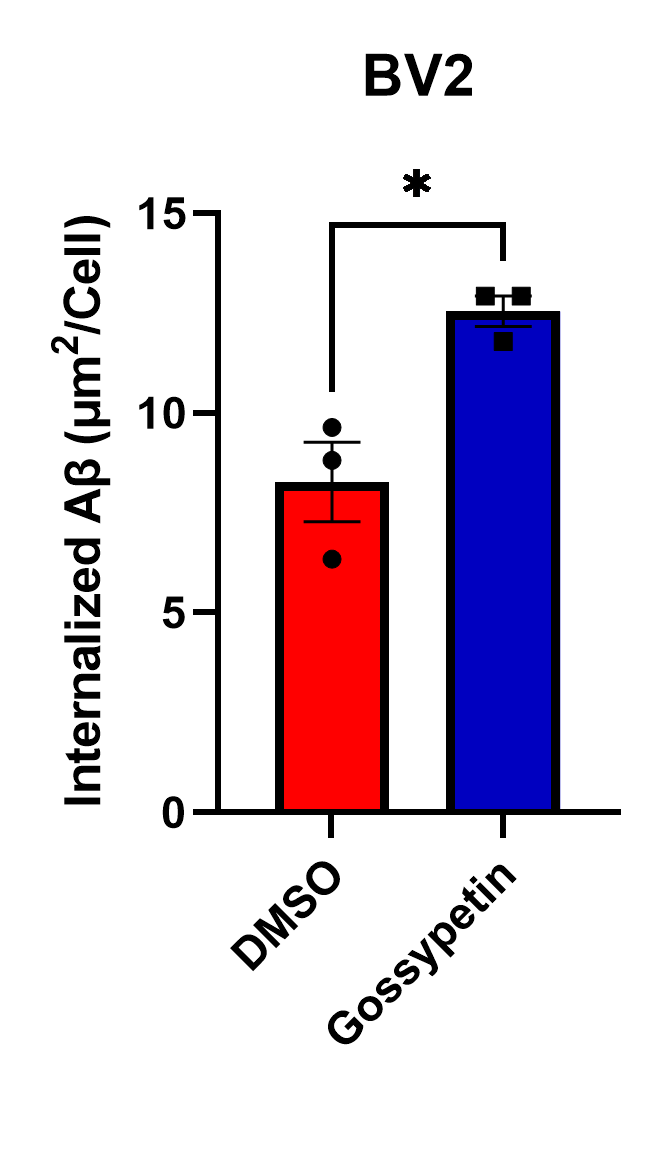

Supplement: Supplementary file 6 — Additional file 6: Fig.S1 Gossypetin does not affect expression of β-, and γ-secretases and activity of β-secretase. (A to G) Time dependent β-secretase activity of mouse hippocampal lysate was measured with Relative Fluorescence Unit (RFU). Fluorescence excitation and emission wavelength was 335 nm and 495 nm respectively (A). Bar graph of RFU at each time point of 10 min (B), 20 min (C), 30 min (D), 40 min (E), 50 min (F), 60 min (G). (n = 10~12 mice per group) (H to L) Representative images of Western blot analysis for β-, γ-secretase subunits, and GAPDH (H). Bar graphs represent relative protein expression levels of BACE1 (I), Nicastrin (J), APH-1 (K), and PEN2 (L). (n = 12~15 mice per group) (M to P) Bar graphs represent relative mRNA expression level of β-, and γ-secretase subunits bace1 (M), ncstn (N), aph1 (O), pen2 (P). (n = 9~10 mice per group) Error bars represent the mean ± SD, *p < 0.05, ns = not significant, two-way ANOVA followed by Tukey’s multiple comparisons test. Fig. S2 Cell type classification of brain samples. (A) UMAP plot showing all cells from the brain samples, colored by their cell types. (B) Heatmap illustrating the Z-scores of average normalized expressions of cell type markers. (C) Violin plots displaying the log-scaled number of detected genes (top), Unique Molecular Identifiers (UMIs) (middle), and the percentage of mitochondrial gene expressions (bottom) per cell for each cell type. (D) UMAP plots showing all cells from the brain samples, colored by their sampled region (left), mouse strain (middle), or drug administration (right) condition. Fig. S3 Detailed subtyping of the microglial population. (A) UMAP plots showing all microglial cells from cortex region. The cells are colored by their celltypes (left). Heatmap showing the Z-scores of average normalized expressions of representative DEGs for each cell type from cortex region (right). (B) UMAP plots showing microglial cells from cortex (left) or hippocampus (right), colored by co [file 13195_2022_1096_MOESM6_ESM.zip › Figure S7B.png]

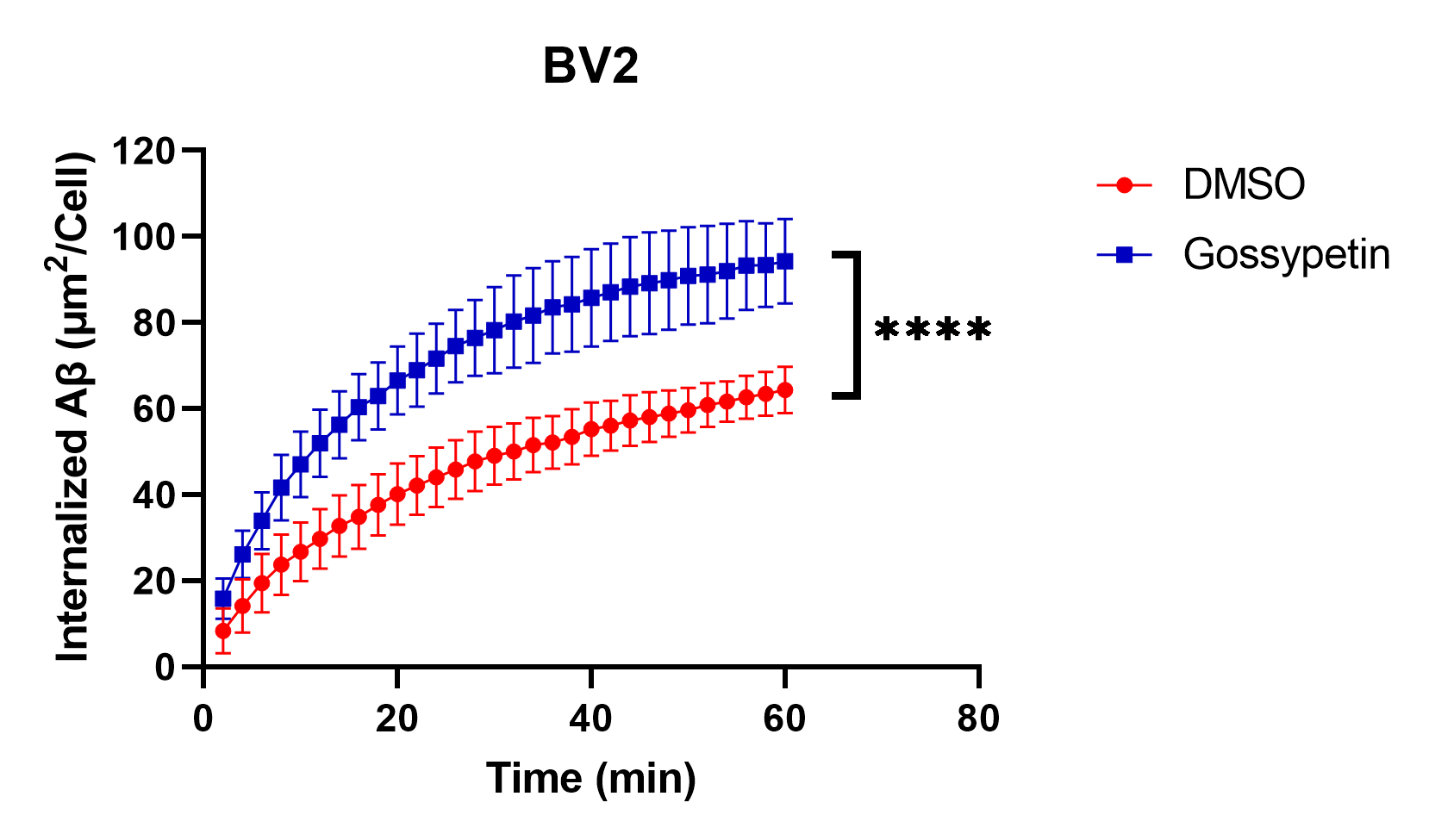

Supplement: Supplementary file 6 — Additional file 6: Fig.S1 Gossypetin does not affect expression of β-, and γ-secretases and activity of β-secretase. (A to G) Time dependent β-secretase activity of mouse hippocampal lysate was measured with Relative Fluorescence Unit (RFU). Fluorescence excitation and emission wavelength was 335 nm and 495 nm respectively (A). Bar graph of RFU at each time point of 10 min (B), 20 min (C), 30 min (D), 40 min (E), 50 min (F), 60 min (G). (n = 10~12 mice per group) (H to L) Representative images of Western blot analysis for β-, γ-secretase subunits, and GAPDH (H). Bar graphs represent relative protein expression levels of BACE1 (I), Nicastrin (J), APH-1 (K), and PEN2 (L). (n = 12~15 mice per group) (M to P) Bar graphs represent relative mRNA expression level of β-, and γ-secretase subunits bace1 (M), ncstn (N), aph1 (O), pen2 (P). (n = 9~10 mice per group) Error bars represent the mean ± SD, *p < 0.05, ns = not significant, two-way ANOVA followed by Tukey’s multiple comparisons test. Fig. S2 Cell type classification of brain samples. (A) UMAP plot showing all cells from the brain samples, colored by their cell types. (B) Heatmap illustrating the Z-scores of average normalized expressions of cell type markers. (C) Violin plots displaying the log-scaled number of detected genes (top), Unique Molecular Identifiers (UMIs) (middle), and the percentage of mitochondrial gene expressions (bottom) per cell for each cell type. (D) UMAP plots showing all cells from the brain samples, colored by their sampled region (left), mouse strain (middle), or drug administration (right) condition. Fig. S3 Detailed subtyping of the microglial population. (A) UMAP plots showing all microglial cells from cortex region. The cells are colored by their celltypes (left). Heatmap showing the Z-scores of average normalized expressions of representative DEGs for each cell type from cortex region (right). (B) UMAP plots showing microglial cells from cortex (left) or hippocampus (right), colored by co [file 13195_2022_1096_MOESM6_ESM.zip › Figure S7C.png]
